# Characterization of the genome of a phylogenetically distinct tospovirus and its interactions with the local lesion-induced host *Chenopodium quinoa* by whole-transcriptome analyses

**DOI:** 10.1371/journal.pone.0182425

**Published:** 2017-08-03

**Authors:** Wan-Chen Chou, Shih-Shun Lin, Shyi-Dong Yeh, Siang-Ling Li, Ying-Che Peng, Ya-Hsu Fan, Tsung-Chi Chen

**Affiliations:** 1 Department of Biotechnology, Asia University, Wufeng, Taichung, Taiwan; 2 Institute of Biotechnology, National Taiwan University, Taipei, Taiwan; 3 Agricultural Biotechnology Research Center, Academia Sinica, Taipei, Taiwan, Taiwan; 4 Department of Plant Pathology, National Chung Hsing University, Taichung, Taiwan; 5 NCHU-UCD Plant and Food Biotechnology Center, National Chung Hsing University, Taichung, Taiwan; 6 Genetech Biotech Co., Ltd., Neihu, Taipei, Taiwan; 7 Department of Medical Research, China Medical University Hospital, China Medical University, Taichung, Taiwan; National University of Singapore, SINGAPORE

## Abstract

*Chenopodium quinoa* is a natural local lesion host of numerous plant viruses, including tospoviruses (family *Bunyaviridae*). Groundnut chlorotic fan-spot tospovirus (GCFSV) has been shown to consistently induce local lesions on the leaves of *C*. *quinoa* 4 days post-inoculation (dpi). To reveal the whole genome of GCFSV and its interactions with *C*. *quinoa*, RNA-seq was performed to determine the transcriptome profiles of *C*. *quinoa* leaves. The high-throughput reads from infected *C*. *quinoa* leaves were used to identify the whole genome sequence of GCFSV and its single nucleotide polymorphisms. Our results indicated that GCFSV is a phylogenetically distinct tospovirus. Moreover, 27,170 coding and 29,563 non-coding sequences of *C*. *quinoa* were identified through *de novo* assembly, mixing reads from mock and infected samples. Several key genes involved in the modulation of hypersensitive response (HR) were identified. The expression levels of 4,893 deduced complete genes annotated using the *Arabidopsis* genome indicated that several HR-related orthologues of pathogenesis-related proteins, transcription factors, mitogen-activated protein kinases, and defense proteins were significantly expressed in leaves that formed local lesions. Here, we also provide new insights into the replication progression of a tospovirus and the molecular regulation of the *C*. *quinoa* response to virus infection.

## Introduction

Tospoviruses are one of the most damaging groups of plant viruses, causing severe losses in economic crops and ornamentals worldwide, and are vectored through thrips in a persistent manner [1]. The genus *Tospovirus* (family *Bunyaviridae*) is named for the type member, *Tomato spotted wilt virus* (TSWV), which has enveloped quasi-spherical particles of 80–120 nm in diameter that possess three genomic single-stranded RNA (ssRNA) segments named large (L), middle (M) and small (S) [2]. The negative sense L RNA contains one large open reading frame (ORF) encoding the RNA-dependent RNA polymerase (RdRp) for replication and transcription [3, 4]. Both the M and S RNA molecules are ambisense, with each comprising two ORFs flanked by an intergenic region (IGR) and encoding proteins in opposite directions. The M RNA encodes a movement protein (NSm) from the viral (v) strand and the surface glycoproteins Gn and Gc from the viral complementary (vc) strand [5, 6]. The v strand of the S RNA encodes the RNA-silencing suppressor (RSS) NSs protein [7, 8], and its vc strand encodes the structural nucleocapsid (N) protein [9]. The 90% amino acid (aa) identity of the N protein is a key threshold for the identification of a tospovirus species. The current tospoviruses can be divided into distinct clades based on the phylogeny of the N protein. The serological relationship of the N proteins is another important criterion for the classification of tospoviruses. Viruses can be classified within a serogroup based on the cross reactivity of their N protein-derived polyclonal antisera [2].

Groundnut chlorotic fan-spot virus (GCFSV, syn. Peanut chlorotic fan-spot virus), collected from central Taiwan in 1993, shares a close phylogenetic relationship with *Groundnut yellow spot virus* (GYSV, syn. *Peanut yellow spot virus*) in terms of the N protein forming a unique evolutionary clade in the genus *Tospovirus* [10]. Additionally, GCFSV and GYSV display similar biological features in terms of host range, specific thrips vector (*Scirtothrips dorsalis* Hood) and serological relatedness [11–13]. The molecular characteristics of GCFSV and GYSV are poorly understood; only the S RNA sequences are available.

Whole genome sequencing is important for the characterization of viruses and is conventionally conducted using Sanger sequencing coupled with the cloning of complementary DNAs (cDNAs) amplified from viral RNAs (v-RNAs). Recently, high-throughput next-generation sequencing (NGS) was applied for the genome sequencing of tospoviruses. During viral infection, small interfering RNAs (siRNAs) corresponding to the viral genome form a proportion of the small RNA population in infected plant tissues. Using NGS for the deep sequencing of siRNAs, tospoviruses such as TSWV, Capsicum chlorosis virus (CaCV) and *Polygonum ringspot virus* (PolRSV) can be diagnosed from diseased plant tissues [14, 15]. The whole-genome sequence of Chrysanthemum stem necrosis virus (CSNV) can be rapidly completed using v-RNA through NGS [16]. In addition to diagnosis, identification and whole-genome sequencing of viruses, NGS presents great advantages in the investigation of the molecular interactions of viruses with their hosts through transcriptome sequencing (RNA-seq) [17].

*Chenopodium* spp., such as *C*. *quinoa* and *C*. *amaranticolor*, are important indicator plants for the isolation of plant viruses, including tospoviruses, through single-lesion transfer [18]. The induction of local lesions on *Chenopodium* spp. leaves localizes and eliminates viruses through programmed cell death (PCD), known as the defensive hypersensitive response (HR) [19, 20]. The HR mechanism of *C*. *amaranticolor* was first described in a previous study [21]. The transcriptomic reads from *C*. *amaranticolor* leaves inoculated with *Tobacco mosaic virus* or *Cucumber mosaic virus* were digitally analyzed to assess the differential expression of genes related to the plant-pathogen interaction pathway, and candidates such as the HR-triggering genes *RIN4* and *RPS5*, the mitogen-activated protein kinase (MAPK) genes *MKK4/5* and *WRKY25/33*, and a non-host resistance gene *NHO1* were found to play important roles during virus-induced HR. However, the HR-related genetic modulation of *C*. *amaranticolor* remains unclear. *C*. *quinoa* is the most used indicator plant in plant virology, but its genetic information in HR-related modulation is poorly understood.

Tospoviruses consistently induce local lesions on *C*. *quinoa* leaves, providing the benefit of virus proliferation for further analyses, such as in viral genome sequencing and protein purification [22]. In the present study, RNA-seq was used to determine the foliar transcriptome of GCFSV-infected *C*. *quinoa* in which local lesions were induced. The transcriptomic reads were used to assemble the complete genomic sequences of GCFSV and the HR-related genes of *C*. *quinoa*. The interactions between GCFSV and *C*. *quinoa* were also discussed.

## Materials and methods

### Virus propagation

The PD-2 isolate of GCFSV collected from groundnut in Changhua, central Taiwan, in 1993, was maintained in the systemic host *N*. *benthamiana* and the local lesion host *C*. *quinoa* through mechanical inoculation as previously described [10]. The inoculated plants were cultivated in a temperature-controlled growth chamber (27°C) with a 12-h light period and a 12-h dark period.

### RNA template preparation

Total RNA was extracted from twelve mock-inoculated or 4-dpi GCFSV-infected leaves of four *C*. *quinoa* plants using the Plant Total RNA Miniprep Purification Kit (GMbiolab, Taichung, Taiwan), following the manufacturer’s instructions. The poly(A)-containing RNA (polyA-RNA) was purified from the total RNA using the TruSeq^®^ RNA Sample Preparation Kit v2 (Illumina, San Diego, USA), following the manufacturer’s instructions. The v-RNAs were isolated from viral nucleocapsids, which were partially purified from 100 g of 4-dpi GCFSV-infected *C*. *quinoa* leaves using a centrifugation method modified from a previous report [23]. Briefly, after centrifugation through a 20% (w/v) sucrose cushion at 25,000 rpm for 2.5 h in a Beckman Type 35Ti rotor, the pellet was resuspended in TBG buffer (10 mM Tris-HCl, pH 8.0, containing 10 mM sodium sulfite, 0.1% cysteine and 10 mM glycine), and 500 μl of the TRIzol^*®*^ reagent (Invitrogen^TM^, Thermo Fisher Scientific, Waltham, MA, USA) was subsequently added to 1 ml of nucleocapsid solution, followed by incubation at room temperature for 5 min. After incubation, 100 μl of chloroform was added, followed by centrifugation at maximum speed using a Legend Micro 17 microcentrifuge (Thermo Scientific, Brookfield, WI) to remove proteins. The aqueous phase was then transferred into a new tube, and 500 μl of pre-cooled isopropanol was added, followed by centrifugation at maximum speed for 10 min. The resulting pellets were washed twice with 1 ml of 70% ethanol, and the air-dried pellets were dissolved in 40 μl of DEPC-treated water and stored at -80°C for further analyses.

### cDNA library preparation and Illumina sequencing

Preparation of cDNA libraries was performed following the Illumina TruSeq^®^ RNA Sample Preparation v2 Guide. Briefly, both polyA-RNAs and v-RNAs were fragmented to sizes of 120 to 210 nt at 94°C for 5 min. The short fragments were used as templates for synthesizing first-strand cDNA using SuperScript II reverse transcriptase (RTase) (Invitrogen^TM^) and random hexamer primers (Illumina) at the condition of 25°C for 10 min, followed by 42°C for 50 min for synthesis and 70°C for 15 min for RTase inactivation. The second strand cDNA was subsequently synthesized using Second Strand Master Mix (Illumina) at 16°C for 1 h. Subsequently, a poly(A) adaptor was added at the 3' end of the resulting short cDNA fragments, followed by ligation of sequencing adaptors using Illumina’s adaptor oligo mix. The 200-bp-long fragments were purified through gel extraction and were further enriched according to the instructions of the Illumina TruSeq^®^ RNA Sample Preparation Kit v2 to prepare the final cDNA library. Sequencing was conducted at Genetech Biotech Co., Ltd. (Taipei, Taiwan) using an Illumina MiSeq system. The polyA-RNA template was employed to synthesize the paired-end reads, and single-end reads were obtained from the v-RNA template.

### Analyses of high-throughput NGS data

The sequences of adaptors and low-quality sequences (p-value was set as 0.01) were preliminarily filtered out from the raw NGS reads. The raw reads were randomly clipped into 23-mers using CLC Genomics Workbench 6.0.2 (CLCbio, Aarhus, Denmark) to facilitate *de novo* sequence assembly. The contigs were then subjected to Basic Local Alignment Search Tool (BLAST) searches against the non-redundant protein database of the National Center for Biotechnology Information (NCBI) (http://www.ncbi.nlm.nih.gov) and annotated to the databases of TAIR and European Molecular Biology Laboratory (EMBL) using ContigViews online software (http://www.contigviews.bioagri.ntu.edu.tw/)) [24]. Functional categorization employing gene ontology (GO) terms [25] and Kyoto Encyclopedia of Genes and Genomes (KEGG) pathways [26] were performed using Blast2GO software [27]. Gene expression levels were compared via the fragments per kilobase of transcript per million mapped reads (FPKM) method [28]. The fold change ratio of log_2_ (FPKM_GCFSV-infected_ / FPKM_Mock-inoculated_) was estimated for analyses of gene expression levels. Ratios > 1 represent gene up-regulation, whereas ratios < -1 represent gene down-regulation.

### Sanger sequencing for GCFSV genomic sequences

Total RNA extracted from the 4-dpi GCFSV-infected *C*. *quinoa* leaves was employed as the template. Reverse transcription (RT) was performed using 4 μg of total RNA mixed with 200 nM individual primers (S1 Table and S1 Fig), 25 U of *Moloney murine leukemia virus* (MMLV) RTase (GMbiolab) and 4 μl of 2.5 mM dNTP mix (Takara, Shiga, Japan). The mixtures were incubated at 42°C for 60 min to synthesize first-strand cDNA, and the reactions were inactivated after heating at 72°C for 15 min. Subsequently, the cDNA was mixed with 2.5 U of Ex *Tag* DNA polymerase (Takara) and heated at 94°C for 2 min. PCR was performed with 35 cycles of strand separation at 94°C for 1 min, annealing at 50–60°C (depending on the Tm values of individual primers) for 2 min and synthesis at 72°C for 3 min, with a final reaction for 7 min at 72°C. All amplicons were cloned using the TOPO TA Cloning Kit (Invitrogen^TM^), according to the manufacturer’s instructions for sequencing. Determination of the DNA sequences was conducted at Mission Biotech Co., Ltd. (Taipei, Taiwan) using an ABI 3730XL DNA Analyzer (Applied Biosystems^TM^, Thermo Fisher Scientific).

Both the 5'- and 3'-ends of the GCFSV L and M RNAs were verified using the rapid amplification of cDNA ends (RACE) method, with minor modifications [29, 30]. Briefly, the first-strand cDNA was synthesized using SuperScriptIII^®^ RTase (200 U) (Invitrogen^TM^) mixed with a 200 nM concentration of specific primers (S1 Table and S1 Fig) at 42°C for 60 min. The template RNA molecules were subsequently removed from the RNA-cDNA hybrids using RNaseH (Invitrogen^TM^). The cDNA fragments were tailed with 200 nM PolyC(3c3t11c) (5'-CCCTTTCCCCCCCCCCC-3') and 15 U of terminal deoxynucleotidyl transferase (TdT) (Takara). The tailed cDNA fragments were employed as templates for PCR amplification after mixing with 2.5 U of Ex *Taq* DNA polymerase (Takara), 200 nM PolyG(11g3a3g) (5'-GGGGGGGGGGGAAAGGG-3') primer complementing to the poly(C) tail and 200 nM of another proper primer (S1 Table and S1 Fig). The amplified DNA fragments were cloned by TOPO TA Cloning Kit (Invitrogen^TM^) for sequencing as described above.

### Analyses of GCFSV genomic sequences

The available genomic sequences of tospoviruses (S2 Table) were obtained from databases at the NCBI website (http://www.ncbi.nlm.nih.gov/). Multiple sequence alignments were performed using the ClustalW program of Biology Workbench from the San Diego Supercomputer Center (SDSC) (http://workbench.sdsc.edu/). The determined sequences were compared with the reference sequences using the Bl2seq program of SDSC. The nt sequences were translated into aa residues using the Sixframe program of SDSC. The homologies of the nt and aa sequences were calculated using the Gap program of SeqWeb, Accelrys (Accelrys Inc., San Diego, CA, USA). The Phylip 3.66 package (Department of Genetics, University of Washington, Seattle) was used for the phylogenetic analyses. Bootstrapping produced 1,000 repeats to generate multiple datasets, and versions of the input datasets were reassembled using the Seqboot program. The distance matrix of the aa sequences was produced using the Protdist program under the PAM matrixes of the Dayhoff model [31]. Phylogenetic branches were set with the Neighbor program using the Neighbor-Joining method [32]. Phylogenetic trees were produced with the Consense program. The putative cleavage sites, transmembrane domains, and *N*- and *O*-linked glycosylation sites of the Gn/Gc precursor of GCFSV were predicted using SignalP 3.0 (http://www.cbs.dtu.dk/services/SignalP/)) [33], SMART (http://smart.embl-heidelberg.de/)) [34], NetNGlyc 1.0 (http://www.cbs.dtu.dk/services/NetNGlyc/) and NetOGlyc 3.1 (http://www.cbs.dtu.dk/services/NetOGlyc/)) [35], respectively.

### Quantitative assay of replication progression during GCFSV infection

Two leaves of *C*. *quinoa* that were post-inoculated with GCFSV for 1–9 days at an interval of 24 h in three independent inoculations were harvested for total RNA extraction as previously described. The total RNA of mock-inoculated *C*. *quinoa* leaves was used as the control. A one-step quantitative real-time reverse transcription-polymerase chain reaction (qRT-PCR) method based on the SYBR Green I system was employed for the assays. All reactions were performed using the StepOnePlus^TM^ instrument (Applied Biosystems^TM^). qRT-PCR was performed with a final volume of 10 μl containing 100 ng of RNA template, 5 μl of KAPA SYBR^®^ FAST qPCR 2X Master mix (KAPA Biosystems Inc., Woburn, MA, USA), 10 U of MMLV RTase (GMbiolab), 0.1 μl of RNase inhibitor (GMbiolab) and the appropriate forward and reverse primers (S3 Table). The qRT-PCR was set as cDNA synthesis at 42°C for 30 min, the inactivation of RTase and activation of hot-start DNA polymerase at 95°C for 5 min, and 35 cycles of three steps: 94°C for 15 sec, 65°C for 30 sec and 75°C for 20 sec for PCR. The C_T_ values from the detection of the *NADH dehydrogenase subunit 5* (*nad5*) [36] and *glyceraldehyde 3-phosphate dehydrogenase* (*GAPDH*) transcripts of *C*. *quinoa* through qRT-PCR were used as the endogenous controls for the relative quantitation (RQ) assays of the v-RNA molecules in the infected plant tissues. Quantitative detection was averaged from three independent runs, with a duplicate for each run.

The RQ formula, ΔΔC_T_ = (C_T_^Target^–C_T_^Endogenous^)_GCFSV-infected_−(C_T_^Target^–C_T_^Endogenous^)_Mock-inoculated_ [37], was described as the change in the expression of the target viral genes relative to plant endogenous genes in the time-course studies. The value of (C_T_^Target^)_Mock-inoculated_ was set as 35 for the calculations. Therefore, the amount of target, normalized to an endogenous reference and relative to a calibrator, is given as the amount of target 2^−ΔΔCT^.

### Quantitative assay of gene expression in *C*. *quinoa*

Total RNAs extracted from the 1-dpi and 4-dpi *C*. *quinoa* leaves infected with GCFSV were used for the analyses. At least 60 leaves of ten GCFSV-inoculated *C*. *quinoa* plants in three different inoculations were harvested for total RNA extraction. Total RNA of mock-inoculated *C*. *quinoa* leaves was used for comparison. The SYBR Green I-based one-step qRT-PCR amplification and RQ calculation were performed using the methods described above. The individual primer pairs employed for qRT-PCR amplification were designed from the sequences of the tested contigs, as shown in S4 Table. The *GAPDH* gene of *C*. *quinoa* was used as a housekeeping control.

## Results

### *De novo* assembly of NGS reads for the analysis of GCFSV genomic sequences

**Fig 1:**
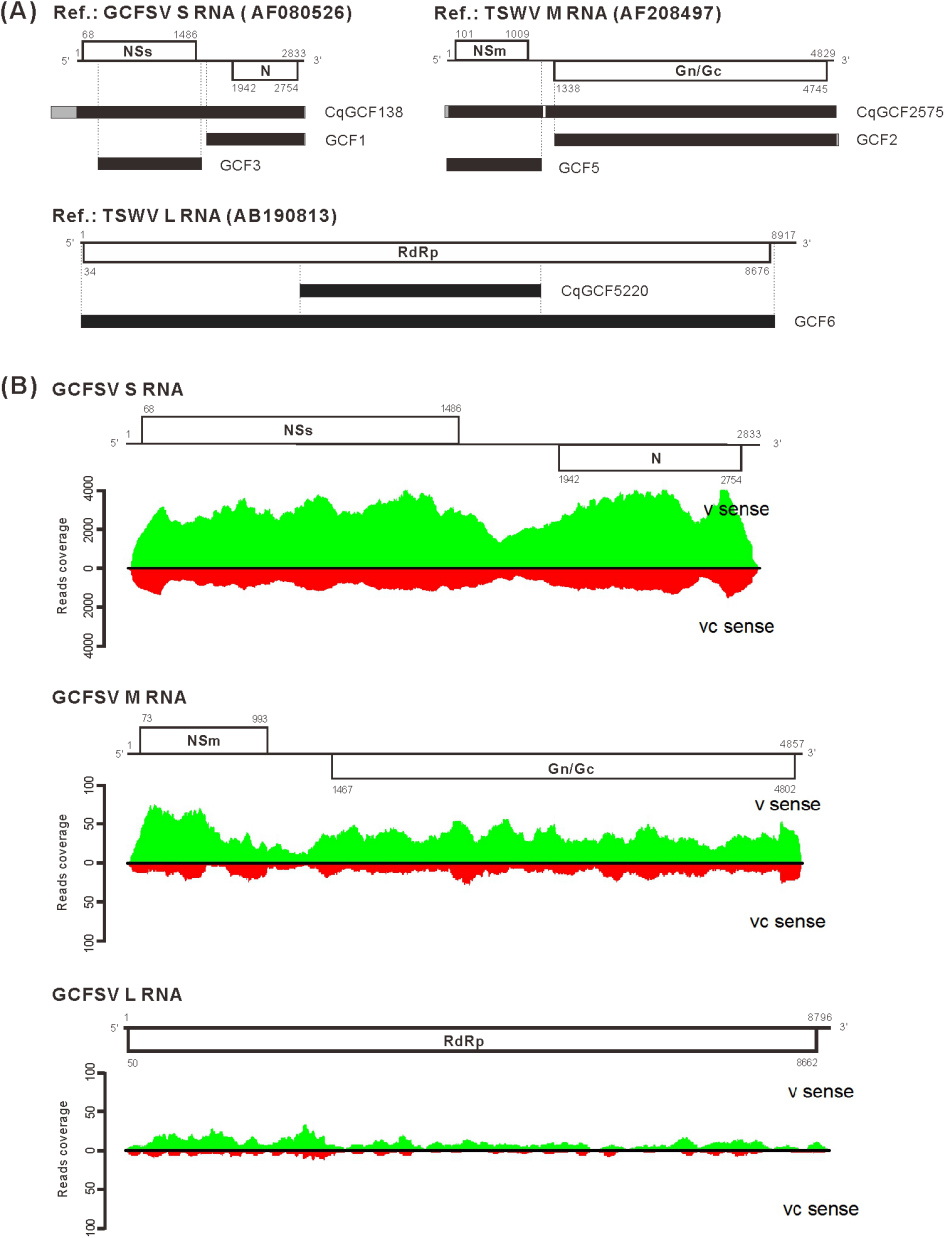
Genome sequencing of Groundnut chlorotic fan-spot virus (GCFSV) via RNA-seq. (**A**) The contigs *de novo* assembled from transcriptomic reads (CqGCF) and viral RNA reads (GCF) were annotated to the reference sequences, for which the accession numbers are indicated. The codes of the contigs are indicated on the right side. The nucleotide positions of the individual open reading frames (ORFs, white boxes) in the reference RNAs are indicated. The ORFs encoded from the viral (v)-sense strand are shown on the upper side. The ORFs encoded from the viral complementary (vc)-sense strand are represented on the lower side. The contigs mapped to the references are shown as black boxes, and the non-mapped sequences are shown as gray boxes. (**B**) Analyses for the read coverage of reads mapping to the S, M and L RNAs of GCFSV. The green and red peaks represent the reads mapping to the v-sense strand and vc-sense strand of RNAs, respectively. The y-axis shows the reads coverage.

The transcriptomic profile of *C*. *quinoa* was obtained from a reads pool from both the mock-inoculated and GCFSV-infected tissues, using polyA-RNAs as templates, to generate 56,733 contigs (designated CqGCF), with an N_50_ of 938 nt, employing a *de novo* assembly method (Table 1). Contig CqGCF138 (3,180 nt), containing the complete GCFSV S RNA sequence, was annotated using the previously determined sequence (acc. AF080526). Reflecting the lack of GCFSV M and L RNA sequences, the M RNA (acc. AF208497) and L RNA (acc. AB190813) sequences of TSWV were used as references to annotate the contigs CqGCF2575 (4,896 nt) and CqGCF5220 (3,030 nt) as GCFSV M and L RNAs, respectively (Fig 1A). Additionally, nucleocapsids purified from the GCFSV-infected *C*. *quinoa* leaves were used to enrich the v-RNA source for deep sequencing, and 10,837,251 reads were *de novo* assembled into 48 contigs, assigned as GCF, with an N_50_ of 2,014 nt. The sequences of CqGCF138, CqGCF2575 and CqGCF5520, corresponding to the S, M and L RNAs of GCFSV, respectively, were used as the references for the BLAST searches with the GCF contigs. GCF1 (1,232 nt) and GCF3 (1,154 nt) mapped to the S RNA; GCF2 (3,549 nt) and GCF5 (1,185 nt) mapped to the M RNA; and GCF6 (8,732 nt) mapped to the L RNA (Fig 1A). Moreover, the full-length sequences of the GCFSV S, M and L RNAs, including the tospovirus-specific 5'-end sequence AGAGCAAU and the 3'-end sequence AUUGCUCU of the GCFSV M and L RNAs, were verified using the Sanger sequencing method with specific primers designed from the NGS-determined sequences for RT-PCR amplification and cloning (S1 Fig).

**Table 1:** Summary of the foliar transcriptome of *Chenopodium quinoa*.

Total number of reads	13,898,870 reads
Mock-inoculated sample	7,474,152 reads
GCFSV-infected sample	6,424,718 reads
Total reads length	1,966,886,610 nt
Paired reads	11,089,180 reads
Paired rate	79.7%
*De novo* assembly	56,733 contigs
N_50_	938 nt
Genome mapping rate	83.13%

Taken together, the GCFSV S RNA sequence obtained through NGS was identical in size (2,833 nt) and shared 99.7% nt identity with the previously reported sequence (acc. AF080526) [10]. The variations within the NSs and N ORFs were confirmed through re-sequencing to demonstrate the correctness of the NGS-generated sequences. The GCFSV M and L RNAs were first resolved. The GCFSV M RNA is 4,857 nt long (acc. KP146141), comprising a 72-nt 5'-untranslatable region (UTR), a 55-nt 3'-UTR and two ORFs in opposite directions, flanked by a 473-nt IGR. The first ORF of the M RNA is 921 nt, encoding a 306 aa (34.3 kDa) NSm protein, and the second ORF is 3,336 nt, encoding a 1,111 aa (126.3 kDa) Gn/Gc precursor protein from the vc strand. The GCFSV L RNA is 8,796 nt in length (acc. KP146140), comprising a 49-nt 5'-UTR, a 134-nt 3'-UTR and an 8,613-nt ORF encoding a 2,870 aa (331.5 kDa) RdRp protein on the vc strand. The GCFSV genome profile is illustrated in S1 Fig.

### Comparison of different NGS approaches for GCFSV genome sequencing

For the S RNA, the contig CqGCF138 covers 100% of the GCFSV S RNA and contains 330 and 17 additional nucleotides at the 5'- and 3'-ends, respectively. However, the contig GCF3 only contains the complete N ORF, and the contig GCF1 contains a 5'-terminal truncated NSs ORF (S2 Fig). For the M RNA, the contig GqGCF2575 covers 99% of the GCFSV M RNA, containing both the complete NSm and Gn/Gc ORFs. The contigs GCF2 and GCF5 contain the complete Gn/Gc and NSm ORFs, respectively (S3 Fig). Unlike the contig CqGCF5220, which covers 34.4% of the GCFSV L RNA, the contig GCF6 covers 99% of the GCFSV L RNA and contains a complete RdRp ORF, but 64 nt of the 5'-UTR and 3 nt of the 3'-UTR were missing (S4 Fig). The transcriptomic reads were analyzed, showing that 72,326 reads (1.1%) mapped to the S RNA, 2,186 reads (0.034%) mapped to the M RNA and 662 reads (0.01%) mapped to the L RNA. Among the GCFSV-corresponding reads, the greatest proportion (96.2%) mapped to the S RNA, whereas 2.9% mapped to the M RNA and 0.9% mapped to the L RNA. These reads showed high coverage of the coding regions on the v strand of the genomic RNAs (Fig 1B).

The reads sequenced from the polyA-RNA and v-RNA were analyzed to detect single nucleotide polymorphisms (SNPs) in the GCFSV genome. The results are summarized in S5 Table. Nucleotide variations were frequently detected at certain positions within the genomic sequences, particularly in the NSs, Gn/Gc and RdRp ORFs. The nucleotides with the highest reads frequencies were selected as the true nucleotides of the viral genome.

### Sequence analyses of the GCFSV L and M RNAs

**Fig 2:**
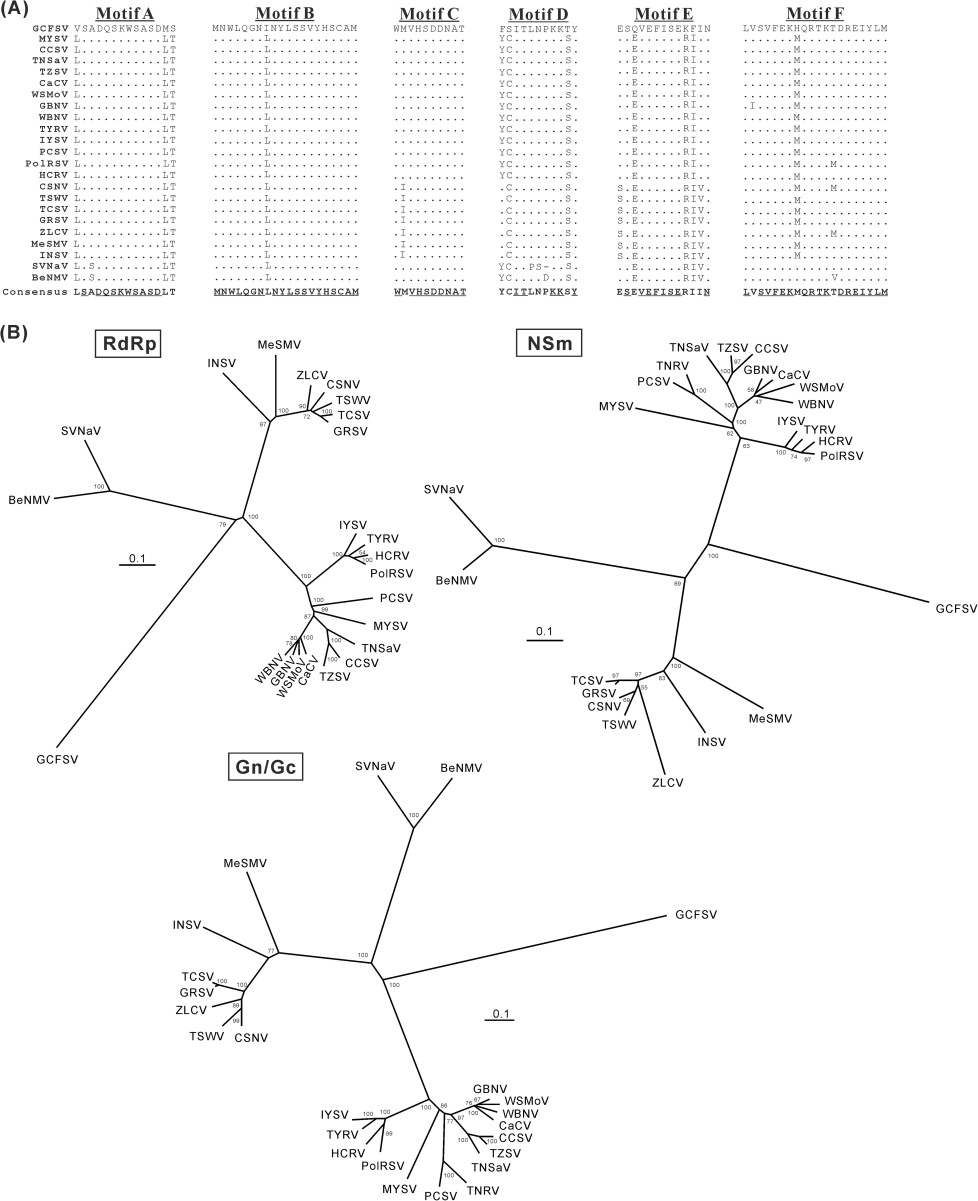
Phylogenetic analyses of the encoded proteins of the L and M RNAs of Groundnut chlorotic fan-spot virus (GCFSV). (**A**) The conserved motifs of the RNA-dependent RNA polymerases (RdRps) of tospoviruses are compared, and the consensus sequences are shown in bold. The identical residues within the same motif are underlined. (**B**) Phylogenetic trees of RdRp, NSm and Gn/Gc precursor. The dendrograms were produced using the Neighbor-Joining algorithm with 1,000 bootstrap replicates. The percentages are shown. See **Table 2**for the virus names.

The RdRp ORF of GCFSV shares low levels of homology, 49.8–52.2% nt identities and 37.7–47.6% aa identities, with those of other tospoviruses (Table 2). Conserved motifs, including motif A (DxxKW, aa 1379–1383), motif B (QGxxxxxSS, aa 1467–1475), motif C (SDD, aa 1505–1507), motif D (K, aa 1552), motif E (ExxS, aa 1562–1565) and motif F (KxQxxxxxR, aa 1300–1308), were identified in the middle portion of GCFSV RdRp (Fig 2A). For the M RNA, the NSm ORF of GCFSV also shares low levels of homology, 47.1–52.2% nt identities and 34.6–44.0% aa identities, with those of other tospoviruses; and the Gn/Gc ORF of GCFSV shares 45.4–48.4% nt identities and 31.1–34.2% aa identities with those of other tospoviruses (Table 2).

**Table 2:** Percentages of nucleotide (nt) and amino acid (aa) identity of the L and M RNA-encoded genes of Groundnut chlorotic fan-spot virus (GCFSV) compared with those of other tospoviruses.

Species	Abbreviation	L RNA		M RNA			
		RdRp		NSm		Gn/Gc	
		nt (%)	aa (%)	nt (%)	aa (%)	nt (%)	aa (%)
Bean necrosis mosaic virus^a^	BeNMV	50.3	37.7	49.3	36.3	46.8	34.2
Calla lily chlorotic spot virus	CCSV	51.1	39.4	49.2	36.7	45.7	32.4
Capsicum chlorosis virus	CaCV	50.9	39.3	49.3	38.6	47.1	31.9
Chrysanthemum stem necrosis virus	CSNV	51.7	39.1	49.8	38.7	46.7	32.1
*Groundnut bud necrosis virus*	GBNV	51.6	39.1	49.9	38.9	47.9	32.5
*Groundnut ringspot virus*	GRSV	51.8	38.9	51.3	36.8	47.2	33.1
Hippeastrum chlorotic ringspot virus	HCRV	50.7	38.7	50.6	44.0	48.0	34.2
*Impatiens necrotic spot virus*	INSV	51.4	39.6	50.5	34.6	46.9	31.2
*Iris yellow spot virus*	IYSV	51.1	38.7	50.2	42.7	46.7	32.5
Melon severe mosaic virus	MeSMV	50.7	47.6	50.1	38.5	46.7	33.7
Melon yellow spot virus	MYSV	49.8	38.4	48.8	40.5	47.8	32.4
Pepper chlorotic spot virus	PCSV	50.9	38.7	51.3	40.9	47.1	32.7
*Polygonum ringspot virus*	PolRSV	49.8	39.1	48.8	42.3	45.4	32.0
Soybean vein necrosis-associated virus	SVNaV	51.1	38.4	50.8	36.5	46.4	32.6
*Tomato chlorotic spot virus*	TCSV	52.2	39.1	52.0	39.0	46.6	32.3
Tomato necrotic ringspot virus	TNRV	-^b^	-	47.1	40.9	46.0	32.0
Tomato necrotic spot associated virus	TNSaV	51.0	39.4	48.3	40.5	47.5	33.4
*Tomato spotted wilt virus*	TSWV	51.3	39.4	52.2	37.0	48.4	31.1
Tomato yellow ring virus	TYRV	51.2	39.1	50.2	42.5	47.6	32.7
Tomato zonate spot virus	TZSV	50.8	39.1	51.0	38.1	47.1	33.1
*Watermelon bud necrosis virus*	WBNV	50.9	38.5	49.5	37.7	46.9	32.1
*Watermelon silver mottle virus*	WSMoV	50.8	38.6	47.8	36.6	47.7	31.9
*Zucchini lethal chlorosis virus*	ZLCV	51.0	47.4	50.2	36.9	46.2	33.3

^a^ Italic typing represents approved species and standard typing represents tentative species.

^b^ “-” represents no genomic sequences are available in GenBank.

The D motif of the 30K movement protein superfamily was found at the aa position 158 of the GCFSV NSm protein. The GCFSV Gn/Gc precursor contains a putative signal cleavage site at the N-terminus between S_39_ and E_40_ and an internal protease cleavage site between A_409_ and L_410_, yielding a 47.1 kDa of Gn protein and a 79.3 kDa of Gc protein. Nine *N*-glycosylation sites and five transmembrane domains, but no *O*-glycosylation site and RGD motif, were observed in the Gn/Gc precursor of GCFSV. Phylogenetic analyses of the RdRp, NSm and Gn/Gc proteins indicated that GCFSV is distantly related from any tospoviruses with sequenced L and M RNAs (Fig 2B).

### Replicative progression of GCFSV genomic RNAs in infected leaves of *C*. *quinoa*

**Fig 3:**
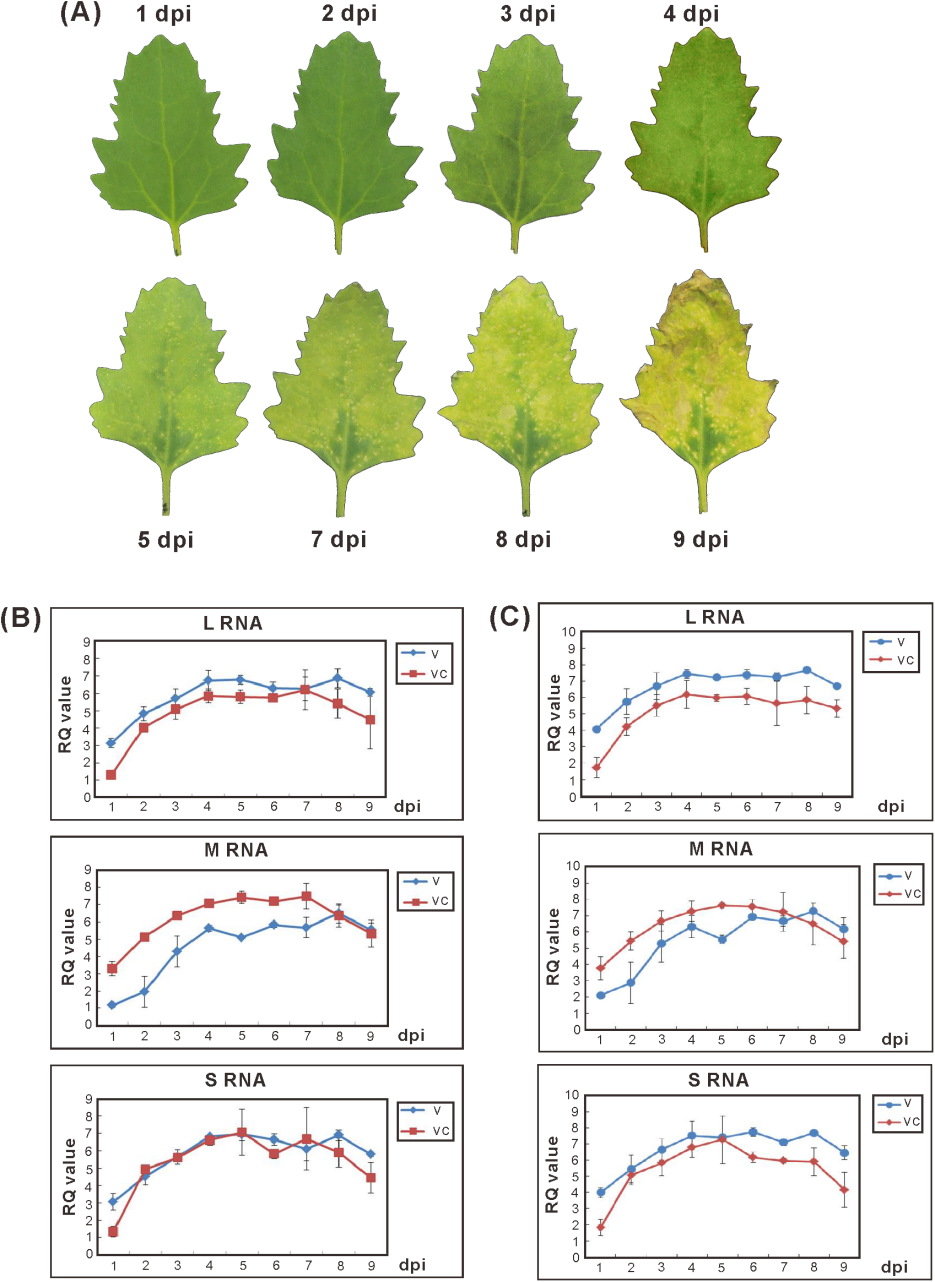
Progression of Groundnut chlorotic fan-spot virus (GCFSV) infection in *Chenopodium quinoa* leaves. (**A**) The development of local lesions on the GCFSV-inoculated *C*. *quinoa* leaves was recorded from the first day post-inoculation (dpi) for 9 days at a 24-h interval. (**B** and **C**) The replication of GCFSV in the leaves of *C*. *quinoa* was analyzed by relative quantitation (RQ) assays. Total RNAs extracted from the leaves described in (**A**) were employed for quantitative real-time reverse transcription-polymerase chain reaction (qRT-PCR) amplification, using the primer pairs corresponding to the viral (v)-sense strand or viral complementary (vc)-sense strand of individual genomic RNAs of GCFSV. The transcripts of the *NADH dehydrogenase subunit 5* (*nad5*) gene (**B**) and the *glyceraldehyde 3-phosphate dehydrogenase* (*GAPDH*) gene (**C**) of *C*. *quinoa* were amplified through qRT-PCR as the endogenous controls for the plants. The averaged C_T_ values from individual amplifications were obtained from three independent runs, with a duplicate in each run. The RQ values were calculated from 2^−ΔΔC^_T_, ΔΔC_T_ = (C_T_^Target^–C_T_^Endogenous^)_GCFSV-infected_−(C_T_^Target^–C_T_^Endogenous^)_Mock-inoculated_. The value of (C_T_^Target^)_Mock-inoculated_ was set as 35 for the calculations.

Symptom development was observed following mechanical inoculation of *C*. *quinoa* leaves. The inoculated leaves showed inconspicuous symptoms at 1–3 dpi. Obvious necrotic local lesions developed on the inoculated leaves at 4–6 dpi, and the leaves gradually developed severe necrosis after 7 dpi (Fig 3A). Both the v and vc strands of the S, M and L RNAs were quantitatively detected in the total RNA of GCFSV-inoculated *C*. *quinoa* leaves from 1–9 dpi. The RQ values for each of the v or vc strands of the GCFSV genomic RNAs were obtained from the average of three independent experiments by comparison with transcripts of both housekeeping genes, *nad5* and *GAPDH*, of *C*. *quinoa* to evaluate the replication of GCFSV. The results showed that the RQ values of all RNA strands, including the v and vc strands, greatly increased at 1–4 dpi during local lesion formation. The RQ values of the v strands of the GCFSV genomic RNAs remained steady at 5–8 dpi and eventually decreased by 9 dpi. Similarly, the RQ values of the vc strands of the GCFSV genomic RNAs remained stable at 5–7 dpi and then decreased at 8 dpi (Fig 3B and 3C).

### The foliar transcriptome of *C*. *quinoa*

**Fig 4:**
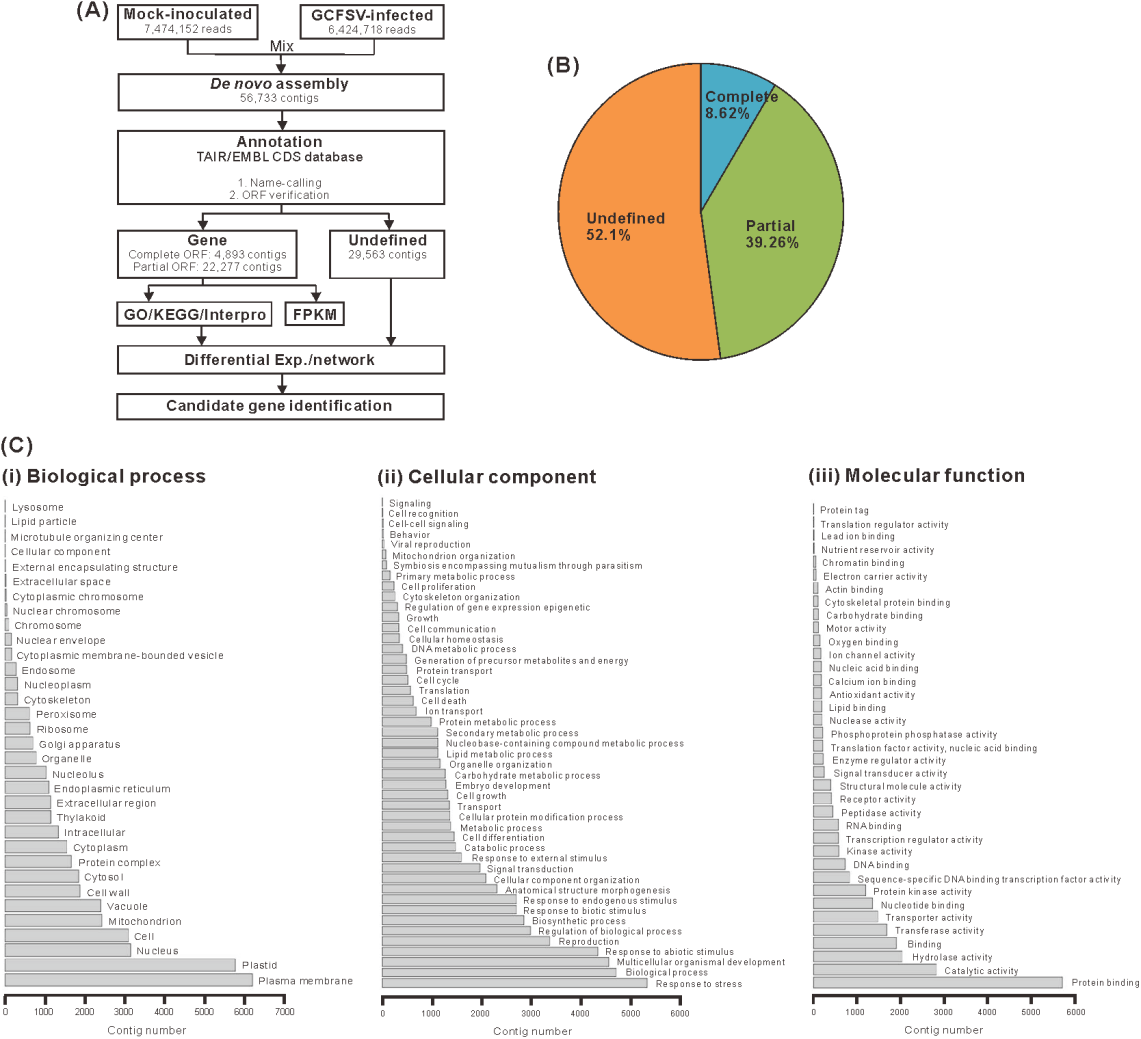
Analyses of the foliar transcriptome of *Chenopodium quinoa*. (**A**) The workflow for the transcriptome analysis. Transcriptomic contigs *de novo* assembled from RNA-seq reads of mock-inoculated and Groundnut chlorotic fan-spot virus (GCFSV)-infected leaves of *C*. *quinoa* were annotated to obtain deduced complete genes. The fragments per kilobase of transcript per million mapped reads (FPKM) method was used to evaluate the differentiation of gene expression between the mock-inoculated and GCFSV-infected leaves of *C*. *quinoa*. The gene functions of the *C*. *quinoa* genes were deduced and classified using the Gene Ontology (GO), Kyoto Encyclopedia of Genes and Genomes (KEGG) and Interpro domain databases. (**B**) The pie chart shows the percentages of complete genes, partial genes and undefined contigs in the whole transcriptome. (**C**) The GO categories of biological processes (i), cellular components (ii) and molecular functions (iii) for the foliar transcriptome of GCFSV-infected *C*. *quinoa* at 4 days post-inoculation (dpi). The x-axis shows the number of contigs in each category. The y-axis indicates the name of a specific category of genes within that main category.

The NGS analysis workflow for the whole transcriptome of *C*. *quinoa* is shown in Fig 4A. The transcriptomic reads for the mock-inoculated and 4-dpi GCFSV-infected leaves of *C*. *quinoa* were analyzed to obtain a comprehensive gene expression profile (Fig 4A). The 56,733 contigs of *C*. *quinoa* were used to construct the transcriptome database employing the ContigViews platform [24]. The raw reads of the transcriptomes reported in this paper are available in the NCBI Short Read Archive under accession numbers SRR4431160 for the mock-inoculated *C*. *quinoa* sample and SRR4431161 for the 4-dpi GCFSV-infected *C*. *quinoa* sample. The transcriptomic contig sequences are available in the ContigViews database (www.contigviews.bioagri.ntu.edu.tw).

**Fig 5:**
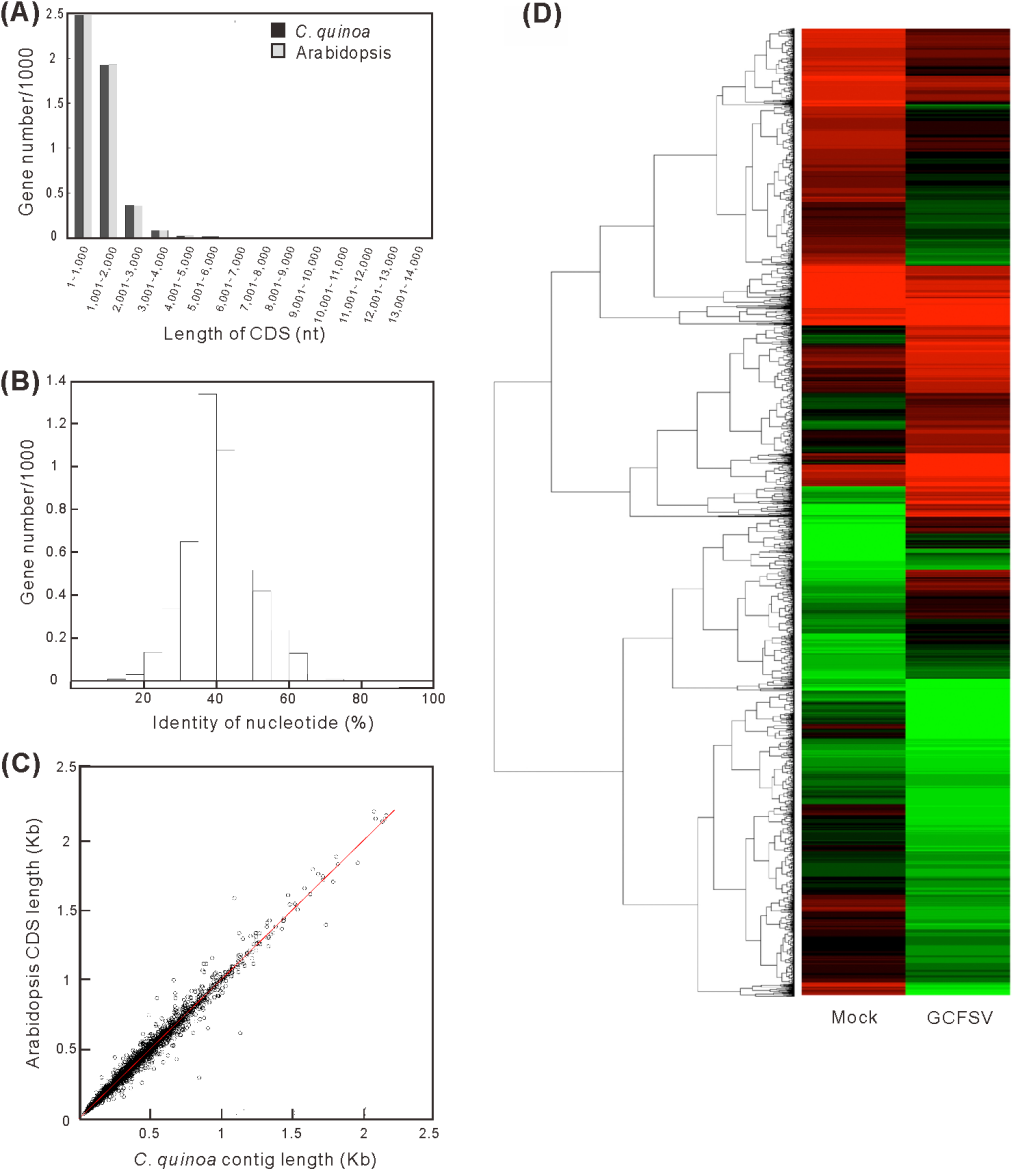
Statistical analysis of gene similarity between *Chenopodium quinoa* and *Arabidopsis*. The length distribution (**A**) and nucleotide identity (**B**) of the coding sequences (CDS) of *C*. *quinoa* and *Arabidopsis* are shown. (**C**) Scatter plot of the CDS length in *C*. *quinoa* compared with that of *Arabidopsis*. (**D**) Relative expression patterns of mock-inoculated and Groundnut chlorotic fan-spot virus (GCFSV)-infected *C*. *quinoa* samples at 4 days post-inoculation (dpi). A relatively lower expression level is indicated in green color; a relatively higher expression level is indicated in red color; and no significant difference is indicated in black color. Genes with similar expression levels are grouped by lines.

The transcriptomic contig sequences were mapped with the genome sequence of *C*. *quinoa* [38] and 83.13% of mapping rate sharing near 100% nt identity was obtained (Table 1), demonstrating the accuracy of the *de novo* assembly for the transcriptome of *C*. *quinoa*. A total of 4,893 complete genes and 22,277 partial genes were predicted in the database and categorized into 117 Gene Ontology (GO) terms (Fig 4B and 4C). A high percentage of the deduced genes were associated with the plasma membrane, protein binding, plastids and stress responses (Fig 4C). The remaining sequences (29,563 contigs) were classified as undefined transcripts. The 4,893 complete genes of *C*. *quinoa* were compared with the coding sequences (CDS) of *Arabidopsis thaliana* Col-0 to reveal the sequence similarities between *C*. *quinoa* and *Arabidopsis*. The length distribution of the major CDS of *C*. *quinoa* as similar to Col-0 was 500 to 1,000 nt (Fig 5A). The average nt identity between *C*. *quinoa* and Col-0 was approximately 40% (Fig 5B). The lengths of the matched CDS between the *C*. *quinoa* and Col-0 plants were highly correlated (R = 0.991) (Fig 5C). In addition, 12,024 of 27,170 genes showed more than 50% coverage in the reference. Among the 4,091 genes, 34% exhibited a two-fold difference in expression during GCFSV infection (Fig 5D). Gene set enrichment analysis (GESA) implicated the involvement of some genes, the false discovery rate (FDR) < 10^−25^, in response to stress (S6 Table).

### Modulation of HR-related genes in GCFSV-infected *C*. *quinoa* leaves

**Fig 6:**
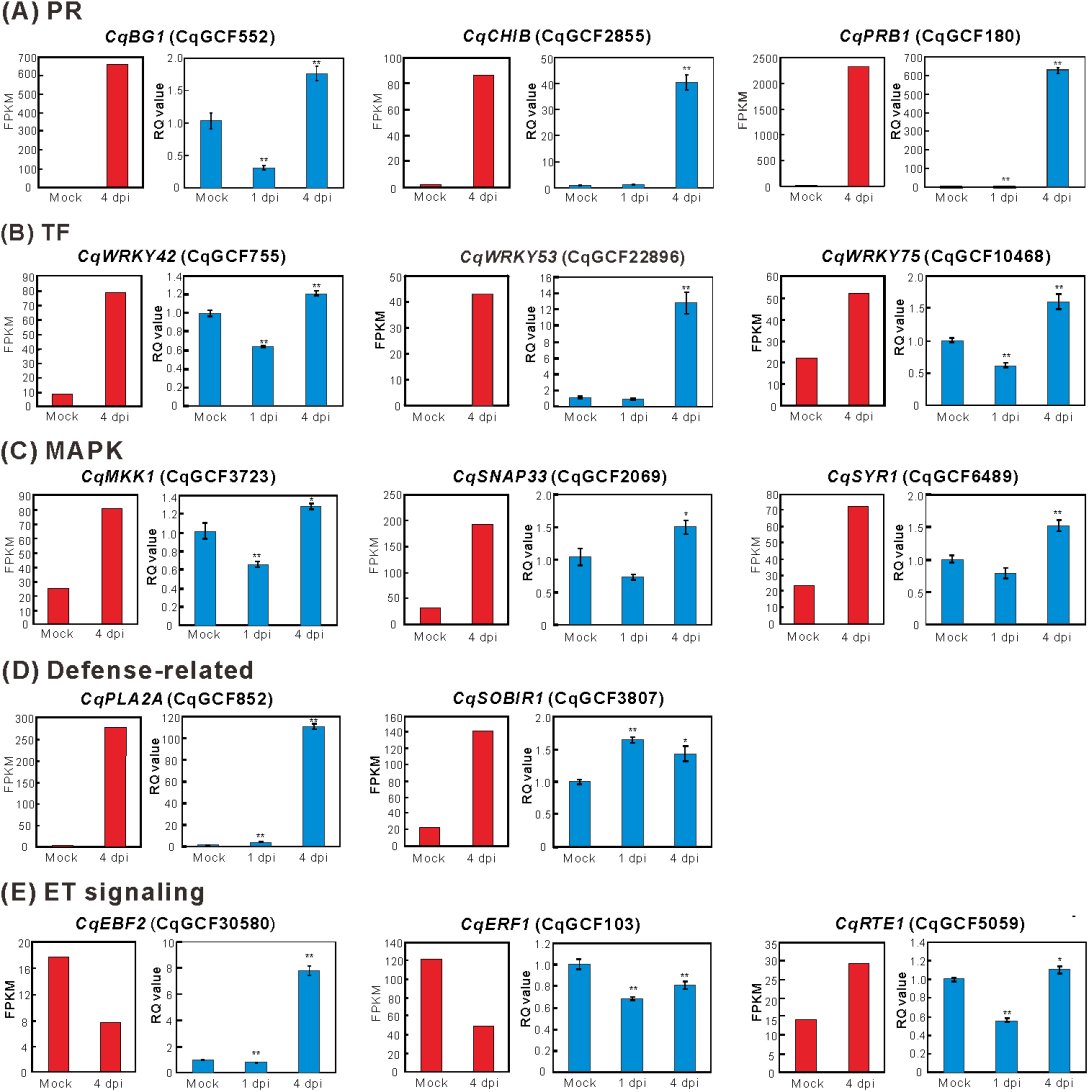
Relative quantitation (RQ) assays of gene modulation in the leaves of *Chenopodium quinoa* inoculated with Groundnut chlorotic fan-spot virus (GCFSV). Hypersensitive response-related genes, including pathogenesis-related (PR) orthologues (**A**), transcription factor (TF) orthologues (**B**), mitogen-activated protein kinase (MAPK) orthologues (**C**), defense-related orthologues (**D**), and ethylene signaling-related orthologues (**E**) were assayed in *C*. *quinoa* using total RNAs extracted from the mock-inoculated and virus-infected leaf tissues at 1 day post-inoculation (dpi) and 4 dpi. The corresponding contig IDs of the assayed genes are indicated in parentheses. The *glyceraldehyde 3-phosphate dehydrogenase* (*GAPDH*) gene of *C*. *quinoa* was used as the comparative reference. The SYBR Green I-based one-step quantitative real-time reverse transcription-polymerase chain reaction (qRT-PCR) amplification and RQ calculation were performed using the 2^−ΔΔCT^ method. Each RQ value was averaged from three independent runs, with a duplicate for each run. The means ± standard errors are presented. The RQ values of the mock-inoculated group were normalized to 1 for comparison in all assays. Significant differences between means compared with mock inoculation were determined using the Student’s *t*-test (*p < 0.05, **p < 0.01). The RQ results are shown in the blue columns (right side). The fragments per kilobase of transcript per million mapped reads (FPKM) results for each assayed gene are shown in the red columns (left side).

The FPKM assay was conducted to evaluate the expression of the deduced complete genes in the mock-inoculated and 4-dpi GCFSV-infected leaves of *C*. *quinoa*. The HR-related orthologues, including pathogenesis-related (PR) proteins, transcription factors (TFs) and MAPKs, were selected for differential gene expression assays using the qRT-PCR method. The RQ values for the mock-inoculations in all assays were normalized to 1 for comparison. The RQ values were calculated from the 4-dpi leaf tissues, in which HR was induced, to represent the gene modulation at the late stage of HR. The 1-dpi symptomless leaf tissues were used to represent the early GCFSV infection. The assayed genes are described in Table 3. The RQ results showed that the PR orthologues *CqBG1*, *CqCHIB* and *CqPRB1*; the TF orthologues *CqWRKY42*, *CqWRKY53* and *CqWRKY75*; the MAPK factors *CqMKK1*, *CqSNAP33* and *CqSYR1*; and the deduced resistance genes *CqPLA2A* and *CqSOBIR1* were significantly up-regulated in *C*. *quinoa* leaves with induced local lesions at 4 dpi with GCFSV, which was in accordance with the predictions of the FPKM assay. Among these genes, *CqPRB1*, *CqPLA2A* and *CqSOBIR1* were significantly up-regulated at the early infection of GCFSV, whereas *CqBG1*, *CqWRKY42*, *CqWRKY75* and *CqMKK1* were down-regulated, and *CqCHIB*, *CqWRKY53*, *CqSNAP33* and *CqSYR1* exhibited no significant differences (Fig 6A–6D).

**Table 3:** Profile of the deduced genes of *Chenopodium quinoa* used for the relative quantitation assay.

Gene name	Contig ID (CqGCF)	Ref. to TAIR	FPKM (Mock-inoculated)	FPKM (GCFSV-infected)	Fold-change (Log_2_)	Description in TAIR
*CqBG1*	552	AT3G57270	2.3332	663.16	8.15	β-1,3-glucanase 1
*CqCHIB*	2855	AT3G12500	2.22743	86.0891	2.40	Basic chitinase, PR3, involved in ET/JA mediated signaling pathway during SAR
*CqEBF2*	30580	AT5G25350	17.73527	7.717624	0.26	EIN3 binding F-box protein, involved in ET signaling pathway, nuclear localized, interacts with EIN3 (ethylene insensitive 3) transcription factor for ubiquitination
*CqERF1*	103	AT4G17500	121.4206	50.06405	0.35	Ethylene responsive element binding factor 1, acts downstream of EIN3 and all other components of ET signaling pathway, defense response
*CqMKK1*	3723	AT4G26070	25.1318	79.8744	0.74	MAPK cascade, HR
*CqPLA2A*	852	AT2G26560	4.20982	276.399	2.59	Phospholipase A 2A, plays a role in cell death and differentially affects the accumulation of oxylipins, contributes to resistance to virus
*CqPRB1*	180	AT2G14580	4.156697	2321.161	9.13	PR protein, encodes a basic PR1-like protein. Expresses in flowers, roots, and not in leaves and responses to ET and methyl JA. SA represses gene expression.
*CqRTE1*	5059	AT2G26070	14.1362	29.3949	0.08	Reversion-to-ethylene sensitivity 1, negative regulation of ET-activated signaling pathway
*CqSNAP33*	2069	AT5G61210	33.3194	192.761	1.34	MAPK cascade
*CqSOBIR1*	3807	AT2G31880	22.7079	142.605	1.41	A putative leucine-rich repeat transmembrane protein, response to fungal and bacterial pathogens
*CqSYR1*	6489	AT3G11820	23.418	72.5435	0.71	MAPK cascade
*CqWRKY42*	755	AT4G04450	7.98835	78.0298	1.72	MAPK cascade
*CqWRKY53*	22896	AT4G23810	10^−8^	42.95444	32.00	Regulation of plant-type HR in SAR by SA-mediated signaling pathway; JA-mediated signaling pathway; leaf senescence; response to H_2_O_2_ and O_3_
*CqWRKY75*	10468	AT5G13080	22.3124	52.057	0.29	MAPK cascade

Additionally, the expressions of the ethylene (ET) signaling-related orthologues, *CqEBF2*, *CqERF1* and *CqRTE1*, in the 1-dpi and 4-dpi *C*. *quinoa* leaves were analyzed. The results indicated that *CqEBF2*, *CqERF1* and *CqRTE1* were all down-regulated in the GCFSV-infected *C*. *quinoa* leaves at 1 dpi. *CqERF1* still exhibited down regulation at 4 dpi, whereas *CqEBF2* and *CqRTE1* became up-regulated (Fig 6E). The 4-dpi RQ results of *CqERF1* and *CqRTE1* were in accordance with the predictions of the FPKM assay, but that of *CqEBF2* was not.

## Discussion

### Genome-wide analyses of the tospovirus GCFSV

Determination of the whole genome sequence of GCFSV helps clarify the integral evolutionary relationships of all known tospoviruses. In this study, the molecular characteristics of the L and M RNAs of GCFSV were first elucidated, including the presence of the conserved motifs of *Tospovirus* RdRp [39], the deduced protease cleavage sites and glycosylation sites of the Gn/Gc precursor [39], the D-motif of the 30K movement protein superfamily in the NSm protein [40], and the expected distinct phylogeny [10]. Determination of the whole genome sequence of GCFSV is important for clarifying the integral evolutionary relationships of all known tospoviruses. Our results indicated that GCFSV is distantly related to any other tospovirus species, as reflected from low nt and aa identities of its genes as compared to those of other tospoviruses (Table 2) and the far genetic distance of the viral genome with those of other tospoviruses (Fig 2B). The approach used in the present study will contribute to solving the problems associated with the whole-genome sequencing of uncharacterized tospoviruses, including GYSV which has been classified in the same clade of GCFSV [10].

Additionally, the SNPs of the tospoviral genome in infected plant tissues were first elucidated. Nucleotide variations are commonly present within the genomic sequences of RNA viruses, reflecting the ineffective proofreading activity of RdRp [41]. We observed more conservation of N and NSm genes in the GCFSV genome; indeed, only one single nucleotide variation was observed in each gene (S5 Table). This finding suggests that both the N and NSm proteins are more conserved for their essential functions in tospovirus infections. The high-read frequency of NGS effectively compensates for the shortcomings of the cloning-based Sanger sequencing method, thereby increasing the fidelity of the genome sequencing of RNA viruses.

Oligo(dT)-primed cDNA libraries have been used in RNA-seq for the whole-genome sequencing of RNA viruses comprising a polyadenylated genome [42]. Here, we report an approach that is also suitable for the genome sequencing of tospovirus. Although no 3' polyadenylated ends were observed in the genomic RNA segments and transcripts of the tospovirus [2], A-rich tandems, such as the AU-rich IGR in the M and S RNAs and the A-rich regions in the L RNA, could be targeted using oligo(dT) primers for cDNA library synthesis. Additional non-viral sequences were observed at the 5' and 3' termini of the S and M RNA sequences in the transcriptomic contigs CqGCF138 and CqGCF2575, suggesting the incorporation of host transcripts through a cap-snatching mechanism [43].

Both v sense- and vc sense-corresponding sequences were obtained from the transcriptomic reads, reflecting the presence of replicative double-stranded RNA (dsRNA) molecules of GCFSV in the infected *C*. *quinoa* tissues (Fig 1B). In addition, the highest and lowest mapping frequencies were observed for the S RNA and L RNA, respectively, based on the analyses of the GCFSV-infected transcriptomic reads (Fig 1B). A similar phenomenon has been reported in previous studies in which deep sequencing of viral siRNAs was conducted for the genome sequencing of TSWV and PolRSV [14,15]. Northern blotting to detect the quantities of S RNA and its corresponding siRNA molecules in a TSWV-infected tomato verified the findings obtained from the siRNA analyses [14]. These results demonstrated that the three tospoviral genomic RNA segments are differentially replicated and/or expressed in plants. The abundant expression of the NSs and N genes of the S RNA is reasonable since the NSs protein suppresses the RNA silencing-mediated resistance of plants and the N protein associates with viral RNA and other components to achieve virus infection [7, 8, 44]. Notably, we cannot exclude the possibility that the relatively lower read mapping frequency of the L RNA might reflect the poor efficiency of RNA isolation and cDNA synthesis because of the lack of AU-rich IGRs in the L RNA.

### Genome-wide analyses of *C*. *quinoa* during GCFSV-triggered HR

The gene modulation that occurs in *C*. *quinoa* in response to GCFSV infection was also investigated. First, the infection progression of GCFSV infection in *C*. *quinoa* leaves was examined. Local lesions developed on the GCFSV-infected *C*. *quinoa* leaves at 4 dpi under constant growth conditions (Fig 3A). Interestingly, we observed numerous local lesions on the GCFSV-inoculated leaves of *C*. *quinoa* plants when 4-dpi leaves were used as the inoculum source, but the number of local lesions significantly reduced when GCFSV-inoculated tissues from 5 dpi or later were used as the inocula, suggesting that the inoculum contains the most active infectious unit with the highest multiplication rate at 4 dpi and that this rate then gradually decreases after 5 dpi, reflecting cell death (Fig 3B). This is the first experimental evidence of the progression of a tospovirus infection in the local lesion-forming host *C*. *quinoa*.

Second, the “down-up” gene modulation represents an organism responds to environmental changes. Our RQ results showed that the PR orthologue *CqBG1*, the TF orthologues *CqWRKY42* and *CqWRKY75*, and the MAPK orthologue *CqMKK1* exhibited significant “down” at 1 dpi, then “up” at 4 dpi in the GCFSV-infected *C*. *quinoa* leaves (Fig 6). Additionally, the significant up-regulation of the PR orthologue *CqPRB1* and the defense-related orthologues *CqPLA2A* and *CqSOBIR1* in the 1-dpi GCFSV-infected *C*. *quinoa* leaves suggests their importance in HR triggering. In *Arabidopsis*, PLA2A plays a role in cell death in response to viruses [45], and SOBIR1 is a putative leucine-rich repeat (LRR) transmembrane protein expressed in response to plant pathogens [46]. Our findings highlight a set of defense genes that could simultaneously be activated in *C*. *quinoa* to overcome virus infections. More defense genes of *C*. *quinoa* should be assayed to explore the essential host factors that are involved in HR.

To our knowledge, β-1,3-glucanases (such as BG1) and chitinases (such as CHIB) are important PR proteins that are abundantly expressed in many plant species after infection with pathogens, particularly fungi and bacteria [47]. The same phenomenon was observed in the present study. According to the descriptions of the Arabidopsis Information Resource (TAIR), CHIB and PRB1 are involved in ET/JA signaling [48], and WRKY53 promotes SA-mediated signaling and suppresses JA responses [49], representing the SA/JA crosstalk in the virus defense responses of *C*. *quinoa*. CqPRB1 is initially expressed at an early stage of HR, indicating its important role in the regulation of SA/JA crosstalk. SA-mediated systemic acquired resistance (SAR) is a non-specific pathogen resistance in plants, but it can be antagonized by other plant hormones, such as JA, ET and abscisic acid (ABA) [50]. Interestingly, we observed that the ET signaling-related orthologues *CqEBF2* and *CqRTE1* were significantly expressed, whereas *CqERF1* was suppressed in the leaves of *C*. *quinoa* with induced local lesions (Fig 6E). In *Arabidopsis*, the binding of ET to receptors, such as ETR1, activates EIN2, which enhances the expression of the TFs EIN3 and EIL1 to initiate the expression of a cascade of downstream genes, such as ERFs and CHIB, in signaling pathways. However, EBFs repress the expression of ERFs [51]. RTE1 promotes the ETR1-mediated repression of ET signaling [52]. The repression of ET responses might reflect the antagonism of the SA signaling pathway. Taken together, our results demonstrate that local lesions formed on the GCFSV-infected leaves of *C*. *quinoa* are the consequence of SAR.

### Interactions between *C*. *quinoa* and viruses

Plants evolve a complex defense network for protection against attacks by RNA viruses for adaptation in nature. Initially, viral replicative dsRNAs trigger RNA silencing, known as pathogen-associated molecular patterns (PAMP)-triggered immunity (PTI), to counteract viruses. However, viruses encode RNA-silencing suppressors to overcome PTI [53]. HR is an effective plant resistance strategy that counteracts virus invasion through the death of infected cells and adjacent cells via signal transduction events [19, 20]. The HR effect involves effector-triggered immunity (ETI) resulting from *R* gene-encoded NB-LRR protein expression in a plant host, which interacts with the corresponding *Avr* gene-encoded effector of a specific pathogen [54]. The Sw-5 is a well-defined R protein that confers specific protection against the tospovirus TSWV in tomato by triggering HR; and the TSWV NSm is an Avr determinant interacting with Sw-5 [55–57].

*Chenopodium* spp. may have a universal receptor to trigger HR for different viruses classified in different genera. We noted that most viruses that induced HR on *C*. *quinoa* can be mechanically transmitted and are not phloem-limited. This effect is probably because the movement process of a virus from the epidermal cells into the parenchyma mesophyll cells of leaf tissues may trigger the localization of the virus to limit its spread. Thus, we hypothesize that the early response of *C*. *quinoa* to virus infections may block plasmodesmata for virus cell-to-cell movement. Localization may be triggered by the movement complex of viruses. Programmed cell death that occurs after the localization of a virus may be due to host RNA silencing triggered by dsRNA formation and siRNA signaling. However, when the silencing effect is antagonized by the virus RSS, *C*. *quinoa* initiates PCD as the defensive reaction to eliminate the invading virus. This hypothesis is well reflected by the finding that mutation of the RSS HC-Pro of a potyvirus reduces its silencing suppression ability and eliminates the HR reaction; however, the localization of the virus still occurred, but the lesion did not form [58, 59].

In addition to HR, SAR can be induced through SA signaling [60]. Our previous study indicated that the loss-of-suppression function of HC-Pro-mutated viruses cannot induce local lesion formation on *C*. *quinoa* plants [58, 59]. The crosstalk of RNA silencing and SA signaling was also demonstrated [59]. Since the RSS NSs of a tospovirus can restore a non-local lesion HC-Pro mutant of ZYMV [61], it is inferred that the GCFSV NSs might also be involved in HR formation on *C*. *quinoa* plants.

Here, we provide new insights into the molecular basis of the progression of a tospovirus infection *in planta* as well as the gene regulation in *C*. *quinoa* leaves for viral defensive HR. In conclusion, these findings suggest that *C*. *quinoa* expresses a signal transduction network primarily modulated through numerous R-related proteins and the SA-mediated pathway to trigger HR in viral resistance. The factors that play a role upstream of ETI in *C*. *quinoa* leaves should be further investigated. Although our data are not on genomic sequences of *C*. *quinoa*, but the almost 100% nt identity between transcriptomic contig sequences and genomic sequences reflecting the accuracy of the transcriptome obtained in this study. The genomic sequences of *C*. *quinoa* can provide an excellent information for further understanding the interaction to HR reaction during virus infection, and also possibly to understand the key host genes for the HR reactions. Our studies of the transcriptome analyses for the HR reaction provide an excellent platform to further understand this important plant-virus interaction. For these concerns, more studies are needed for further studies. The understanding of the regulatory mechanism of HR of *C*. *quinoa* can provide valuable information for the molecular breeding of economic crops against plant viruses.

## Supporting information

S1 Fig Strategy for Sanger sequencing of the L, M and S RNAs of the PD-2 isolate of Groundnut chlorotic fan-spot virus (GCFSV). The nucleotide positions of the individual open reading frames (ORFs) (boxes), including RNA-dependent RNA polymerase (RdRp) in the L RNA **(A)**, NSm and Gn/Gc in the M RNA **(B)**, and NSs and N in the S RNA **(C)** are indicated. The upper boxes represent the ORFs encoded from the viral sense and the lower boxes represent the ORFs encoded from the viral complementary sense. The amplified DNA fragments are shown by bold lines. The primers used for reverse transcription-polymerase chain reaction are indicated by arrows. The sequences of the primers are listed in **S3 Table**.
(PDF)

S2 Fig Comparison of the sequences of the Groundnut chlorotic fan-spot virus (GCFSV) S RNA as determined by next-generation sequencing (NGS) and Sanger sequencing methods. Transcriptome and viral RNA were sequenced by RNA-seq. The previously reported GCFSV S RNA sequence (GenBank: AF080526) determined by Sanger’s method was used for comparison. The underlines indicate the nucleotides that are lost in the obtained sequences. The bold characters represent the nucleotides that are diverse among the three sequences.
(PDF)

S3 Fig Comparison of the sequences of the Groundnut chlorotic fan-spot virus (GCFSV) M RNA as determined by next-generation sequencing (NGS) and Sanger sequencing methods. The nucleotide sequences from the transcriptome and viral RNA sequenced by RNA-seq are aligned with those sequenced by the Sanger method for comparison. The underlines indicate the nucleotides that are lost in the obtained sequences. The bold characters represent the nucleotides that are diverse among the three sequences.
(PDF)

S4 Fig Comparison of the sequences of the Groundnut chlorotic fan-spot virus (GCFSV) L RNA as determined by next-generation sequencing (NGS) and Sanger sequencing methods. The nucleotide sequences from the transcriptome and viral RNA sequenced by RNA-seq are aligned with those sequenced by the Sanger method for comparison. The underlines indicate the nucleotides that are lost in the obtained sequences. The bold characters represent the nucleotides that are diverse among the three sequences.
(PDF)

S1 Table Primers used for the genome sequencing of Groundnut chlorotic fan-spot virus. (PDF)

S2 Table The accession numbers of the L and M RNA sequences of tospoviruses used for the analyses in this study. (PDF)

S3 Table The primers used for the quantitative analyses of replication of Groundnut chlorotic fan-spot virus (GCFSV) in *Chenopodium quinoa* leaves by real-time reverse transcription-polymerase chain reaction. (PDF)

S4 Table The primers used for the relative quantitative assays of hypersensitive response-related gene expression of *Chenopodium quinoa* leaves infected with Groundnut chlorotic fan-spot virus in real-time reverse transcription-polymerase chain reaction. (PDF)

S5 Table Single nucleotide polymorphisms (SNPs) of genomic sequences of Groundnut chlorotic fan-spot virus (GCFSV), as determined by next-generation sequencing (NGS). (PDF)

S6 Table Significant Gene Ontology (GO) terms from the gene set enrichment analysis for the 4,091 Groundnut chlorotic fan-spot virus-responsive genes (false discovery rate, FDR < 10^−25^). (PDF)
