# Supplementary material for: Characterization of the genome of a phylogenetically distinct tospovirus and its interactions with the local lesion-induced host Chenopodium quinoa by whole-transcriptome analyses
Source: PLoS One. 2017 Aug 3;12(8):e0182425. doi: 10.1371/journal.pone.0182425 (PMC5542687; doi:10.1371/journal.pone.0182425)
Supplement: S1 Fig — The nucleotide positions of the individual open reading frames (ORFs) (boxes), including RNA-dependent RNA polymerase (RdRp) in the L RNA (A), NSm and Gn/Gc in the M RNA (B), and NSs and N in the S RNA (C) are indicated. The upper boxes represent the ORFs encoded from the viral sense and the lower boxes represent the ORFs encoded from the viral complementary sense. The amplified DNA fragments are shown by bold lines. The primers used for reverse transcription-polymerase chain reaction are indicated by arrows. The sequences of the primers are listed in S3 Table. (PDF) [file pone.0182425.s001.pdf]

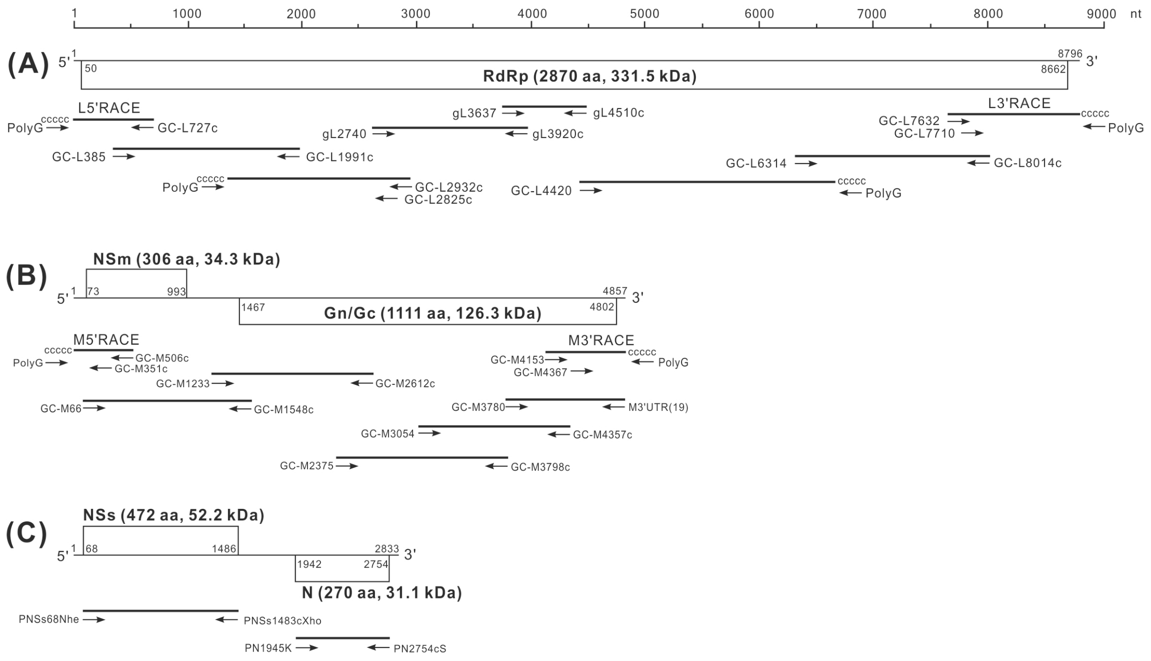

S1 Fig. Strategy for Sanger sequencing of the L, M and S RNAs of the PD-2 isolate of Groundnut chlorotic fan-spot virus (GCFSV). The nucleotide positions of the individual open reading frames (ORFs) (boxes), including RNA-dependent RNA polymerase (RdRp) in the L RNA **(A)**, NSm and Gn/Gc in the M RNA **(B)**, and NSs and N in the S RNA **(C)** are indicated. The upper boxes represent the ORFs encoded from the viral sense and the lower boxes represent the ORFs encoded from the viral complementary sense. The amplified DNA fragments are shown by bold lines. The primers used for reverse transcription-polymerase chain reaction are indicated by arrows. The sequences of the primers are listed in **S3 Table**.
