# Supplementary material for: Characterization of the genome of a phylogenetically distinct tospovirus and its interactions with the local lesion-induced host Chenopodium quinoa by whole-transcriptome analyses
Source: PLoS One. 2017 Aug 3;12(8):e0182425. doi: 10.1371/journal.pone.0182425 (PMC5542687; doi:10.1371/journal.pone.0182425)
Supplement: S2 Fig — Transcriptome and viral RNA were sequenced by RNA-seq. The previously reported GCFSV S RNA sequence (GenBank: AF080526) determined by Sanger’s method was used for comparison. The underlines indicate the nucleotides that are lost in the obtained sequences. The bold characters represent the nucleotides that are diverse among the three sequences. (PDF) [file pone.0182425.s002.pdf]

```
Transcriptome NGS   CCACCACTTTATTACATCCTCTTAAAATTTCGTATATTTTTTTTTTTTGA  50
Viral RNA NGS       --------------------------------------------------
S RNA (AF080526)    --------------------------------------------------
Consensus

Transcriptome NGS   CAAATGGTTCTTTAATTAATTCGATAAATAACAAAAGAGACACACTCAAT  100
Viral RNA NGS       --------------------------------------------------
S RNA (AF080526)    --------------------------------------------------
Consensus

Transcriptome NGS   GACTCTGAACCACCACTTTATTACATCCTCTTAAAATTTCGTATATTTTT  150
Viral RNA NGS       --------------------------------------------------
S RNA (AF080526)    --------------------------------------------------
Consensus

Transcriptome NGS   CCAACATACTATAATAATACGTAAAAAGAGCGGTTTTTTCAATTTAAAAT  200
Viral RNA NGS       --------------------------------------------------
S RNA (AF080526)    --------------------------------------------------
Consensus

Transcriptome NGS   AGAAATTAATACTGCAATAAAACTCGACCGACCTCCCAAAACAAAGAAAA  250
Viral RNA NGS       --------------------------------------------------
S RNA (AF080526)    --------------------------------------------------
Consensus

Transcriptome NGS   TAAAATAAAGATAGGAACAATATGGGTTGAATGCAATGTAATTGATCTTG  300
Viral RNA NGS       --------------------------------------------------
S RNA (AF080526)    --------------------------------------------------
Consensus

Transcriptome NGS   ATCAATTAACCCCAATACATGTGAATTCCAAGAGCAATCAAGGCAAAAAA  350
Viral RNA NGS       --------------------------------------------------
S RNA (AF080526)    ------------------------------AGAGCAATCAAGGCAAAATA
Consensus
                                                                 → NSs
Transcriptome NGS   TAAAAACACATAAACGCTAATCCCAAGACTTAATCCTAATTCCAAAGATG  400
Viral RNA NGS       --------------------------------------------------
S RNA (AF080526)    TAAAAACACATAAACGCTAATCCCAAGACTTAATCCTAATTCCAAAGATG
Consensus

Transcriptome NGS   TCTACCGAGTCGTCCAACCCTGGAAGTACTGAGAATGTAGCACTAATTCC  450
Viral RNA NGS       --------------------------------------------------
S RNA (AF080526)    TCTACCGAGTCGTCCAACCCTGGAAGTACTGAGAATGTAGCACTAATTCC
Consensus

Transcriptome NGS   AGAGCCATTTGATGAGCAGTACTGCGAGGAAGCACTGACATCATATGGTA  500
Viral RNA NGS       --------------------------------------------------
S RNA (AF080526)    AGAGCCATTTGATGAGCAGTACTGCGAGGAAGCACTGACATCATATGGTA
Consensus

Transcriptome NGS   ACAATCTTGACAAATCTGCTCTTGTCGATGTGTACTCGATGTTTGAAGAT  550
Viral RNA NGS       --------------------------------------------------
S RNA (AF080526)    ACAATCTTGACAAATCTGCTCTTGTCGATGTGTACTCGATGTTTGAAGAT
Consensus

Transcriptome NGS   GATAAATTGGTTTTCCAATCTATCCATGCCACAAATGGGAGGTTCAAAGC  600
Viral RNA NGS       -------------------------------------GGAGGTTCAAAGC
S RNA (AF080526)    GATAAATTGGTTTTCCAATCTATCCATGCCACAAATGGGAGGTTCAAAGC
Consensus                                                **************

Transcriptome NGS   TACGTGTGCTTTCGGCCATTCTGACATGGTTCTGGATTCTGAGCAGGAAT  650
Viral RNA NGS       TACGTGTGCTTTCGGCCATTCTGACATGGTTCTGGATTCTGAGCAGGAAT
S RNA (AF080526)    TACGTGTGCTTTCGGCCATTCTGACATGGTTCTGGACTCTGAGCAGGAAT
Consensus           ************************************ *************

Transcriptome NGS   TGAAGACCTCTATTAGCAGCGAGATTTTCCACCAAGAGTTTGATGCCATA  700
Viral RNA NGS       TGAAGACCTCTATTAGCAGCGAGATTTTCCACCAAGAGTTTGATGCCATA
S RNA (AF080526)    TGAAGACCTCTATTAGCAGCGAGATTTTCCACCAAGAGTTTGATGCCATA
Consensus           **************************************************
```

```
Transcriptome NGS    TCAGGAATAAACTCAATAATCAGTCTCCAAGGGGAAAACATCCACGTCAT  750
Viral RNA NGS        TCAGGAATAAACTCAATAATCAGTCTCCAAGGGGAAAACATCCACGTCAT
S RNA (AF080526)     TCAGGAATAAACTCAATAATCAGTCTCCAAGGGGAAAACATCCACGTCAT
Consensus            **************************************************

Transcriptome NGS    TATATCTCATCCTGAACAGAAGGTCAAATCATACAAATATGCATTCCATG  800
Viral RNA NGS        TATATCTCATCCTGAACAGAAGGTCAAATCATACAAATATGCATTCCATG
S RNA (AF080526)     TATATCTCATCCTGAACAGAAGGTCAAATCATACAAATATGCATTCCATG
Consensus            **************************************************

Transcriptome NGS    GAAGAATTGCTTCGAATGATGTCTTTCCGAGAATTTCAGGGTTTGATGAA  850
Viral RNA NGS        GAAGAATTGCTTCGAATGATGTCTTTCCGAGAATTTCAGGGTTTGATGAA
S RNA (AF080526)     GAAGAATTGCTTCGAATGATGTCTTTCCGAGAATTTCAGGGTTTGATGAA
Consensus            **************************************************

Transcriptome NGS    TTTCGATCACAGTATTCATGCAATCCTTACAAGTTTGTGTACAATCCTGG  900
Viral RNA NGS        TTTCGATCACAGTATTCATGCAATCCTTACAAGTTTGTGTACAATCCTGG
S RNA (AF080526)     TTTCGATCACAGTATTCATGCAATCCTTACAAGTTTGTGTACAATCCTGG
Consensus            **************************************************

Transcriptome NGS    CATGTTGGGTCTTGAGTCTGAAACAAACACTGTCATATTTCCTGTTGAAA  950
Viral RNA NGS        CATGTTGGGTCTTGAGTCTGAAACAAACACTGTCATATTTCCTGTTGAAA
S RNA (AF080526)     CATGTTGGGTCTTGAGTCTGAAACAAACACTGTCATATTTCCTGTTGAAA
Consensus            **************************************************

Transcriptome NGS    AGCTAGGCACTCTTCCGGCTCATGCCTCAGCTTTTGGGAACAATTTTGAC  1000
Viral RNA NGS        AGCTAGGCACTCTTCCGGCTCATGCCTCAGCTTTTGGGAACAATTTTGAC
S RNA (AF080526)     AGCTAGGCACTCTTCCGGCTCATGCCTCAGCTTTTGGGAACAATTTTGAC
Consensus            **************************************************

Transcriptome NGS    CGCTGTCACTTCTCAGGGTCATCTGTTCCAGGTTTCATGACAGTCAAAGC  1050
Viral RNA NGS        CGCTGTCACTTCTCAGGGTCATCTGTTCCAGGTTTCATGACAGTCAAAGC
S RNA (AF080526)     CGCTGTCACTTCTCAGGGTCATCTGTTCCAGGTTTCATGACAGTCAAAGC
Consensus            **************************************************

Transcriptome NGS    TGTTTCTGAACCATCCAAGAGGACCCAAGAAAAGGCTCATCGGCAGTTAC  1100
Viral RNA NGS        TGTTTCTGAACCATCCAAGAGGACCCAAGAAAAGGCTCATCGGCAGTTAC
S RNA (AF080526)     TGTTTCTGAATCATCCAAGAGGACCCAAGAAAAGGCTCATCGGCAGTTAC
Consensus            ********** ***************************************

Transcriptome NGS    ACAACAAATCTCTGAAGGCTCTAGAAATTGTTTCAAACTCAGATTTTAGA  1150
Viral RNA NGS        ACAACAAATCTCTGAAGGCTCTAGAAATTGTTTCAAACTCAGATTTTAGA
S RNA (AF080526)     ACAACAAATCTCTGAAGGCTCTAGAAATTGTTTCAAACTCAGATTTTAGA
Consensus            **************************************************

Transcriptome NGS    TCAACATACATCACCAAAACCACTCTCAAAGCCAGGAAAAATGGCATGAT  1200
Viral RNA NGS        TCAACATACATCACCAAAACCACTCTCAAAGCCAGGAAAAATGGCATGAT
S RNA (AF080526)     TCAACATACATCACCAAAACCACTCTCAAAGCCAGGAAAAATGGCATGAT
Consensus            **************************************************

Transcriptome NGS    GACTATCCAAGTCCAGGTAAAAGATCTCCACTGCGAGCCTGAAAAGAGAG  1250
Viral RNA NGS        GACTATCCAAGTCCAGGTAAAAGATCTCCACTGCGAGCCTGAAAAGAGAG
S RNA (AF080526)     GACTATCCAAGTCCAGGTAAA-GATCTCCACTGCGAGCCTGAAAAGAGAG
Consensus            ********************* ****************************

Transcriptome NGS    AGCATCTTATTGCCATCCCAGACGACCCTTTGAAGAGGTTAGTTTATTGC  1300
Viral RNA NGS        AGCATCTTATTGCCATCCCAGACGACCCTTTGAAGAGGTTAGTTTATTGC
S RNA (AF080526)     --CATCTTATTGCCATCCCAGATGACCCTTTGAAGAGGTTAGTTTATTGC
Consensus              ******************** ***************************

Transcriptome NGS    TCCTCTGAGTGCTTGGGACAGACTTCTCCAGGATTATCTACATTTATCTT  1350
Viral RNA NGS        TCCTCTGAGTGCTTGGGACAGACTTCTCCAGGATTATCTACATTTATCTT
S RNA (AF080526)     TCCTCTGAGTGCTTGGGACAGACTTCTCCAGGATTATCTACATTTATCTT
Consensus            **************************************************

Transcriptome NGS    TGAAGTGATCTGTTTGGACTGCTTAGTTGACAGGGTAACACCTGATGTCA  1400
Viral RNA NGS        TGAAGTGATCTGTTTGGACTGCTTAGTTGACAGGGTAACACCTGATGTCA
S RNA (AF080526)     TGAAGTGATGTGTTTGGACTGCTTAGTTGACAGGGTAACACCTGATGTCA
Consensus            ********* ****************************************
```

```
Transcriptome NGS    CTTACTTTGCAGGCGCACTCTCAACCTGTTTCACTGATGAACCACCTCAC  1450
Viral RNA NGS        CTTACTTTGCAGGCGCACTCTCAACCTGTTTCACTGATGAACCACCTCAC
S RNA (AF080526)     CTTACTTTGCAGGCGCACTCTCAACCTGTTTCACTGATGAACCACCTCAC
Consensus            **************************************************

Transcriptome NGS    ATTCTTCTCAAAGCTAGGTCAGGAACACTTTCATCTCTGGCTGACTCAGA  1500
Viral RNA NGS        ATTCTTCTCAAAGCTAGGTCAGGAACACTTTCATCTCTGGCTGACTCAGA
S RNA (AF080526)     ATTCTTCTCAAAGCTAGGTCAGGAATACTTTCATCTCTGGCTGACTCAGA
Consensus            ************************* ************************

Transcriptome NGS    CAGAGATAAGATCAATGAGATAATCTGCAAGAACCTAGTGAGTGTACATC  1550
Viral RNA NGS        CAGAGATAAGATCAATGAGATAATCTGCAAGAACCTAGTGAGTGTACATC
S RNA (AF080526)     CAGAGATAAGATCAATGAGATAATCTGCAAGAACCTAGTGAGTGTACATC
Consensus            **************************************************

Transcriptome NGS    TTGGATTGGCCACAGATTTGGCAAAGAAGCTTAACAAACCGATAAATGTT  1600
Viral RNA NGS        TTGGATTGGCCACAGATTTGGCAAAGAAGCTTAACAAACCGATAAATGTT
S RNA (AF080526)     TTGGATTGGCCACAGATTTGGCAAAGAAGCTTAACAAACCGATAAATGTT
Consensus            **************************************************

Transcriptome NGS    TTCACTATCAAGGACACATCTTCCATGACCACAGACATGGTTGAAGTGGA  1650
Viral RNA NGS        TTCACTATCAAGGACACATCTTCCATGACCACAGACATGGTTGAAGTGGA
S RNA (AF080526)     TTCACTATCAAGGACACATCTTCCATGACCACAGACATGGTTGAAGTGGA
Consensus            **************************************************

Transcriptome NGS    TGGGAAAAAGTATAGAGTCCTGAAAGATTCAAATGGAGACTACTACTTCA  1700
Viral RNA NGS        TGGGAAAAAGTATAGAGTCCTGAAAGATTCAAATGGAGACTACTACTTCA
S RNA (AF080526)     TGGGAAAAAGTATAGAGTCCTGAAAGATTCAAATGGAGACTACTACTTCA
Consensus            **************************************************

Transcriptome NGS    CTTCAGCAACATTCGAGAAGACTCTTATTGGAGCTTATAAGTCTTGTCAA  1750
Viral RNA NGS        CTTCAGCAACATTCGAGAAGACTCTTATTGGAGCTTATAAGNNNNNNNNN
S RNA (AF080526)     CTTCAGCAACATTCGAGAAGACTCTTATTGGAGCTTATAAGTCTTGTCAA
Consensus            *****************************************

Transcriptome NGS    ACTTTTGTCGACTATTGTGACAATAAGAAGCTCAGCATCAACGGCGATAA  1800
Viral RNA NGS        NNNNNNNNNNNNNNNNNNNNNNNNNNNNNNNNNNNNNNNNNNNNNNNNNN
S RNA (AF080526)     ACTTTTGTCGACTATTGTGACAATAAGAAGCTCAGCATCAACGGCGATAA
Consensus

                                   NSs end <--┐
Transcriptome NGS    TGTTTTTATCTTCAATTAGATTTCTGTTTCTTCACAAACCTTGATTATTG  1850
Viral RNA NGS        NNNNNNNNNNNNNNNNNNNNNNNNNNNNNNNNNNNNNNNNNNNNNNNNNN
S RNA (AF080526)     TGTTTTTATCTTCAATTAGATTTCTGTTTCTTCACAAACCTTGATTATTG
Consensus

Transcriptome NGS    TTAGTAGATTTAAAAAACTACAAATGATAATAACCAAGTAAATCTAATAC  1900
Viral RNA NGS        NNNNNNNNNNNNNNNNNNNNNNNNNNNNNNNNNNNNNNNNNNNNNNNNNN
S RNA (AF080526)     TTAGTAGATTTAAAAAACTACAAATGATAATAACCAAGTAAATCTAATAC
Consensus

Transcriptome NGS    CAGATAGATCAGAAGAAATTCAGAAATCAAAATAAATAAGCAAAAATCC  1950
Viral RNA NGS        NNNNNNNNNNNNNNNNNNNNNNNNNNNNNNNNNNNNNNNNNNNNNNNNCC
S RNA (AF080526)     CAGATAGATCAGAAGAAATTCAGAAATCAAAATAAATAAGCAAAAATCC
Consensus                                                            **

Transcriptome NGS    AAAAAAATCAAAAAACAAAAACC--AAAAAATCAAAAAATCAGAAA-CAA  2000
Viral RNA NGS        AAAAAAATCAAAAAACAAAAACC-AAAAAAATCAAAAAATCAGAAA-CAA
S RNA (AF080526)     AAAAAAATCAAAAAACAAAAAACCAAAAAAATCAAAAAATCAGAAAACAA
Consensus            ********************* *  ********************* ***

Transcriptome NGS    AAAAAGAGGACTTCGGTCCCATCAATGGCAAGTATACTTACTGCAACTTC  2050
Viral RNA NGS        AAAAAGAGGACTTCGGTCCCATCAATGGCAAGTATACTTACTGCAACTTC
S RNA (AF080526)     AAAAAGAGGACTTCGGTCCCATCAATGGCAAGTATACTTACTGCAACTTC
Consensus            **************************************************

Transcriptome NGS    ATCTATAAACAGACCAAATTTGGGGATTTCTCCCTCTTTTTTTGTTTTTG  2100
Viral RNA NGS        ATCTATAAACAGACCAAATTTGGGGATTTCTCCCTCTTTTTTTGTTTTTG
S RNA (AF080526)     ATCTATAAACAGACCAAATTTGGGGATTTCTCCCTCTTTTTTTGTTTTTG
Consensus            **************************************************
```

```
Transcriptome NGS    TTTGTTTTATTTATTTTTATTTAAATTTCTTATTTTT-CTCTTATCTGGT  2150
Viral RNA NGS        TTTGTTTTATTTATTTTTATTTAAATTTCTTATTTTT-CTCTTATCTGGT
S RNA (AF080526)     TTTGTTTTATTTATTTTTATTTAAATTTCTTATTTTTTCTCTTATCTGGT
Consensus            ************************************* ************

Transcriptome NGS    TTATTTCAAATACTAGTAAACATCACAATATCACAATATATTGAGGTAGC  2200
Viral RNA NGS        TTATTTCAAATACTAGTAAACATCACAATATCACAATATATTGAGGTAGC
S RNA (AF080526)     TTATTTCAAATACTAGTAAACATCACAATATCACAATATATTGAGGTAGC
Consensus            **************************************************

Transcriptome NGS    TTAAATGCAGACACAGGTACAGTGGAAAAGCATAAAATAGTGAATAACAT  2250
Viral RNA NGS        TTAAATGCAGACACAGGTACAGTGGAAAAGCATAAAATAGTGAATAACAT
S RNA (AF080526)     TTAAATGCAGACACAGGTACAGTGAAAAAGCATAAAATAGTGAATAACAT
Consensus            ************************ *************************

Transcriptome NGS    CGATATATAGAGAACTTAATATATTTAAAACAGAAAAGCTTTAACAATCA  2300
Viral RNA NGS        CGATATATAGAGAACTTAATATATTTAAAACAGAAAAGCTTTAACAATCA
S RNA (AF080526)     CGATATATAGAGAACTTAATATATTTAAAACAGAAAAGCTTTAACAATCA
Consensus            **************************************************

Transcriptome NGS    ATAAATAAATATTAAGTACTTGATATAACATCACCTGTCTCCATGGCACT  2350
Viral RNA NGS        ATAAATAAATATTAAGTACTTGATATAACATCACCTGTCTCCATGGCACT
S RNA (AF080526)     ATAAATAAATATTAAGTACTTGATATAACATCACCTGTCTCCATGGCACT
Consensus            **************************************************

                     → N end
Transcriptome NGS    TCAAAGTTCACCGCTGCCTATGGCATTCTGGAGATCATTGTATTTCGAAG  2400
Viral RNA NGS        TCAAAGTTCACCGCTGCCTATGGCATTCTGGAGATCATTGTATTTCGAAG
S RNA (AF080526)     TCAAAGTTCACCGCTGC-TATGGCATTCTGGAGATCATTGTATTTCGAAG
Consensus            ***************** ********************************

Transcriptome NGS    ACTGATCCTTGCTATCACTCTTGGCTTTTGGCACTGCATTTCTGAAATAA  2450
Viral RNA NGS        ACTGATCCTTGCTATCACTCTTGGCTTTTGGCACTGCATTTCTGAAATAA
S RNA (AF080526)     ACTGATCCTTGCTATCACTCTTGGCTTTTGGCACTGCATTTCTGAAATAA
Consensus            **************************************************

Transcriptome NGS    GACTCAATCAGAGTCTTTGGTGTTGGTGATGGCAAAAGAGAGCACCTTTC  2500
Viral RNA NGS        GACTCAATCAGAGTCTTTGGTGTTGGTGATGGCAAAAGAGAGCACCTTTC
S RNA (AF080526)     GACTCAATCAGAGTCTTTGGTGTTGGTGATGGCAAAAGAGAGCACCTTTC
Consensus            **************************************************

Transcriptome NGS    CCCAATTACAACCATTCTATCCAATTGAGCTTTAGTGTCAAAATTCTTGA  2550
Viral RNA NGS        CCCAATTACAACCATTCTATCCAATTGAGCTTTAGTGTCAAAATTCTTGA
S RNA (AF080526)     CCCAATTACAACCATTCTATCCAATTGAGCTTTAGTGTCAAAATTCTTGA
Consensus            **************************************************

Transcriptome NGS    TTCCCAGCTCTTCTGATCTTTTGTGTTGATAATTTGCAATGGCAAAAGCA  2600
Viral RNA NGS        TTCCCAGCTCTTCTGATCTTTTGTGTTGATAATTTGCAATGGCAAAAGCA
S RNA (AF080526)     TTCCCAGCTCTTCTGATCTTTTGTGTTGATAATTTGCAATGGCAAAAGCA
Consensus            **************************************************

Transcriptome NGS    TGTGCAAATGTCGTCTTAAAACCCATGAGGTGATTCAAGTTGCCTCCAGT  2650
Viral RNA NGS        TGTGCAAATGTCGTCTTAAAACCCATGAGGTGATTCAAGTTGCCTCCAGT
S RNA (AF080526)     TGTGCAAATGTCGTCTTAAAACCCATGAGGTGATTCAAGTTGCCTCCAGT
Consensus            **************************************************

Transcriptome NGS    CACCACTTTGAAGATGACCTCATCTCTTGGAGACATTGCATCGGTATGTA  2700
Viral RNA NGS        CACCACTTTGAAGATGACCTCATCTCTTGGAGACATTGCATCGGTATGTA
S RNA (AF080526)     CACCACTTTGAAGATGACCTCATCTCTTGGAGACATTGCATCGGTATGTA
Consensus            **************************************************

Transcriptome NGS    GATTGAATACCTCAAGTAACCAGTTTGTCAGTTTTGCATTTTCTGCCTTC  2750
Viral RNA NGS        GATTGAATACCTCAAGTAACCAGTTTGTCAGTTTTGCATTTTCTGCCTTC
S RNA (AF080526)     GATTGAATACCTCAAGTAACCAGTTTGTCAGTTTTGCATTTTCTGCCTTC
Consensus            **************************************************

Transcriptome NGS    ATCCTGTCATCACCATAATCCTTAACTCTTTCAATGTACTTCATTCTGAT  2800
Viral RNA NGS        ATCCTGTCATCACCATAATCCTTAACTCTTTCAATGTACTTCATTCTGAT
S RNA (AF080526)     ATCCTGTCATCACCATAATCCTTAACTCTTTCAATGTACTTCATTCTGAT
Consensus            **************************************************
```

```
Transcriptome NGS    CAGAGAGTCCATCCTGCTCCAAGTGAATTCGTTCACACCTGGTGAACTCT  2850
Viral RNA NGS        CAGAGAGTCCATCCTGCTCCAAGTGAATTCGTTCACACCTGGTGAACTCT
S RNA (AF080526)     CAGAGAGTCCATCCTGCTCCAAGTGAATTCGTTCACACCTGGTGAACTCT
Consensus            **************************************************

Transcriptome NGS    TCACAATGCTGTGACCAAGATATTTAGAAACACTACTCTCTCTTCCTATG  2900
Viral RNA NGS        TCACAATGCTGTGACCAAGATATTTAGAAACACTACTCTCTCTTCCTATG
S RNA (AF080526)     TCACAATGCTGTGACCAAGATATTTAGAAACACTACTCTCTCTTCCTATG
Consensus            **************************************************

Transcriptome NGS    GCTTTCATCTTGTTGGAATTGTTGATGAAAAGTATCATGTTTTCATCTGA  2950
Viral RNA NGS        GCTTTCATCTTGTTGGAATTGTTGATGAAAAGTATCATGTTTTCATCTGA
S RNA (AF080526)     GCTTTCATCTTGTTGGAATTGTTGATGAAAAGTATCATGTTTTCATCTGA
Consensus            **************************************************

Transcriptome NGS    GATCTCAACTTTTGAATTATTCTCAGAAAAGAATTTCTTGAATGTGAAGT  3000
Viral RNA NGS        GATCTCAACTTTTGAATTATTCTCAGAAAAGAATTTCTTGAATGTGAAGT
S RNA (AF080526)     GATCTCAACTTTTGAATTATTCTCAGAAAAGAATTTCTTGAATGTGAAGT
Consensus            **************************************************

Transcriptome NGS    TTGCTTGTTCTTGAGATAACTCAATTTTGGCATTCCTTGACAAAGACTCA  3050
Viral RNA NGS        TTGCTTGTTCTTGAGATAACTCAATTTTGGCATTCCTTGACAAAGACTCA
S RNA (AF080526)     TTGCTTGTTCTTGAGATAACTCAATTTTGGCATTCCTTGACAAAGACTCA
Consensus
                                                       N <-┐
Transcriptome NGS    AACAGCTCCTTGTCATTCTTGACTTTGGTTTTAGACATCTTCGAACTGGT  3100
Viral RNA NGS        AACAGCTCCTTGTCATTCTTGACTTTGGTTTTAGACATCTTCGAACTGGT
S RNA (AF080526)     AACAGCTCCTTGTCATTCTTGACTTTGGTTTTAGACATCTTCGAACTGGT
Consensus            **************************************************

Transcriptome NGS    TTAAGATTTTGGGAAAAGTAGTGTTGTAGATTTGGGTTAAATGATTATTT  3150
Viral RNA NGS        TTAAGATTTTGGGAAAAGTAGTGTTGTAGATTTGGGTTAAATGATTATTT
S RNA (AF080526)     TTAAGATTTTGGGAAAAGTAGTGTTGTAGATTTGGGTTAAATGATTATTT
Consensus            **************************************************

Transcriptome NGS    ATTGCCTTGATTGCTCTTATTGGAGCTTATAAGT                  3200
Viral RNA NGS        ATTGCCTTGATTGCTCTTATTGGAGCTTATAAG-
S RNA (AF080526)     ATTGCCTTGATTGCTCT-----------------
Consensus            *****************
```

S2 Fig. Comparison of the sequences of the Groundnut chlorotic fan-spot virus (GCFSV) S RNA as determined by next-generation sequencing (NGS) and Sanger sequencing methods. Transcriptome and viral RNA were sequenced by RNA-seq. The previously reported GCFSV S RNA sequence (GenBank: AF080526) determined by Sanger's method was used for comparison. The underlines indicate the nucleotides that are lost in the obtained sequences. The bold characters represent the nucleotides that are diverse among the three sequences.
