# Supplementary material for: Characterization of the genome of a phylogenetically distinct tospovirus and its interactions with the local lesion-induced host Chenopodium quinoa by whole-transcriptome analyses
Source: PLoS One. 2017 Aug 3;12(8):e0182425. doi: 10.1371/journal.pone.0182425 (PMC5542687; doi:10.1371/journal.pone.0182425)
Supplement: S3 Fig — The nucleotide sequences from the transcriptome and viral RNA sequenced by RNA-seq are aligned with those sequenced by the Sanger method for comparison. The underlines indicate the nucleotides that are lost in the obtained sequences. The bold characters represent the nucleotides that are diverse among the three sequences. (PDF) [file pone.0182425.s003.pdf]

```
Transcriptome NGS   ACCACCGAGATCTACACTCTTTCCCTACACGACGCTCTTCCGATCTCCGG  50
Viral RNA NGS       ----------------------------------CAATAAGAGCAATCGG
Sanger              ---------------------------------------AGAGCAATCGG
Consensus                                                          ***

Transcriptome NGS   CACACAAAAAAGATATTCAAACAAAGACTTCAAACATTTCCAATTATAAC  100
Viral RNA NGS       CACACAAAAAAGATATTCAAACAAAGACTTCAAACATTTCCAATTATAAC
Sanger              CACACAAAAAAGATATTCAAACAAAGACTTCAAACATTTCCAATTATAAC
Consensus           **************************************************

                               → NSm
Transcriptome NGS   CTCGGGTAAATATGGACATGATCAGCAGGATAGGCAGCAGTATCAACTCC  150
Viral RNA NGS       CTCGGGTAAATATGGACATGATCAGCAGGATAGGCAGCAGTATCAACTCC
Sanger              CTCGGGTAAATATGGACATGATCAGCAGGATAGGCAGCAGTATCAACTCC
Consensus           **************************************************

Transcriptome NGS   ACCGTAACAAATGGATTGCAAAATCTTGGTAGAACAAATGAAGACACTGA  200
Viral RNA NGS       ACCGTAACAAATGGATTGCAAAATCTTGGTAGAACAAATGAAGACACTGA
Sanger              ACCGTAACAAATGGATTGCAAAATCTTGGTAGAACAAATGAAGACACTGA
Consensus           **************************************************

Transcriptome NGS   TGTTTTAGGTTCTGATAATAGAACTCTTATGAGGATGCCTACCAAAAGAG  250
Viral RNA NGS       TGTTTTAGGTTCTGATAATAGAACTCTTATGAGGATGCCTACCAAAAGAG
Sanger              TGTTTTAGGTTCTGATAATAGAACTCTTATGAGGATGCCTACCAAAAGAG
Consensus           **************************************************

Transcriptome NGS   ATGCTGAGAAAGCAAGAGAAACCAGACTGCAGGGATTCAAAGAAAGAAAT  300
Viral RNA NGS       ATGCTGAGAAAGCAAGAGAAACCAGACTGCAGGGATTCAAAGAAAGAAAT
Sanger              ATGCTGAGAAAGCAAGAGAAACCAGACTGCAGGGATTCAAAGAAAGAAAT
Consensus           **************************************************

Transcriptome NGS   GTCATAGAATCAGGGCCATCTGAGCTAGGTGAGTATGATGGAAACCCTGT  350
Viral RNA NGS       GTCATAGAATCAGGGCCATCTGAGCTAGGTGAGTATGATGGAAACCCTGT
Sanger              GTCATAGAATCAGGGCCATCTGAGCTAGGTGAGTATGATGGAAACCCTGT
Consensus           **************************************************

Transcriptome NGS   GATCACATCAGATTTGAGCATACTAGAGAGGCTGGAAGTCAATACTTCAA  400
Viral RNA NGS       GATCACATCAGATTTGAGCATACTAGAGAGGCTGGAAGTCAATACTTCAA
Sanger              GATCACATCAGATTTGAGCATACTAGAGAGGCTGGAAGTCAATACTTCAA
Consensus           **************************************************

Transcriptome NGS   ACCATATCAGCAATTGGAAAACTGATGTGTTTCTAGGGAATGGGCAAGAA  450
Viral RNA NGS       ACCATATCAGCAATTGGAAAACTGATGTGTTTCTAGGGAATGGGCAAGAA
Sanger              ACCATATCAGCAATTGGAAAACTGATGTGTTTCTAGGGAATGGGCAAGAA
Consensus           **************************************************

Transcriptome NGS   ATGGTGAAGAAGGACATAAATCTCATACCAACATGGGATTCCAAAAAAGA  500
Viral RNA NGS       ATGGTGAAGAAGGACATAAATCTCATACCAACATGGGATTCCAAAAAAGA
Sanger              ATGGTGAAGAAGGACATAAATCTCATACCAACATGGGATTCCAAAAAAGA
Consensus           **************************************************

Transcriptome NGS   GTACATGCAAATCAGTAGAGTTATAATCTGGATTGTTCCAGTGGCACCTG  550
Viral RNA NGS       GTACATGCAAATCAGTAGAGTTATAATCTGGATTGTTCCAGTGGCACCTG
Sanger              GTACATGCAAATCAGTAGAGTTATAATCTGGATTGTTCCAGTGGCACCTG
Consensus           **************************************************

Transcriptome NGS   GCACCAAAGGGAAAATCAAAGCAGCATTGGTTGATAGGAACAAGGCAGAG  600
Viral RNA NGS       GCACCAAAGGGAAAATCAAAGCAGCATTGGTTGATAGGAACAAGGCAGAG
Sanger              GCACCAAAGGGAAAATCAAAGCAGCATTGGTTGATAGGAACAAGGCAGAG
Consensus           **************************************************

Transcriptome NGS   TCTGAACAGATAATCTTCCAGAAAGAAGGGGTTCTGACTGATCCATTGTG  650
Viral RNA NGS       TCTGAACAGATAATCTTCCAGAAAGAAGGGGTTCTGACTGATCCATTGTG
Sanger              TCTGAACAGATAATCTTCCAGAAAGAAGGGGTTCTGACTGATCCATTGTG
Consensus           **************************************************

Transcriptome NGS   TTTCATCTTTTACATGCACTGGTCTTATTTAAAATCCGTGAATAACAAAG  700
Viral RNA NGS       TTTCATCTTTTACATGCACTGGTCTTATTTAAAATCCGTGAATAACAAAG
Sanger              TTTCATCTTTTACATGCACTGGTCTTATTTAAAATCCGTGAATAACAAAG
Consensus           **************************************************
```

```
Transcriptome NGS    CTCTCTGCCCTCAACTTAAATTTATAAGTAATGAAGTATATAAGAAAAAT  750
Viral RNA NGS        CTCTCTGCCCTCAACTTAAATTTATAAGTAATGAAGTATATAAGAAAAAT
Sanger               CTCTCTGCCCTCAACTTAAATTTATAAGTAATGAAGTATATAAGAAAAAT
Consensus            **************************************************

Transcriptome NGS    GTCCCCTTTGCTGTGGCCAACTTTGCATGGAGAAAGGTTTTTTGCAACTC  800
Viral RNA NGS        GTCCCCTTTGCTGTGGCCAACTTTGCATGGAGAAAGGTTTTTTGCAACTC
Sanger               GTCCCCTTTGCTGTGGCCAACTTTGCATGGAGAAAGGTTTTTTGCAACTC
Consensus            **************************************************

Transcriptome NGS    ACCAATTGCAATGACAGAGGTGCAGCCTGATCTTATTGTATTAAATAGAG  850
Viral RNA NGS        ACCAATTGCAATGACAGAGGTGCAGCCTGATCTTATTGTATTAAATAGAG
Sanger               ACCAATTGCAATGACAGAGGTGCAGCCTGATCTTATTGTATTAAATAGAG
Consensus            **************************************************

Transcriptome NGS    GTATAACTATAAGGAACAAAGCTCTCCTCGAAGCTGTGAAGTGTATTGTA  900
Viral RNA NGS        GTATAACTATAAGGAACAAAGCTCTCCTCGAAGCTGTGAAGTGTATTGTA
Sanger               GTATAACTATAAGGAACAAAGCTCTCCTCGAAGCTGTGAAGTGTATTGTA
Consensus            **************************************************

Transcriptome NGS    CCTAATGGGAATAATGGAAAAACTATAAAGAAGCAAATAGAATCACTATC  950
Viral RNA NGS        CCTAATGGGAATAATGGAAAAACTATAAAGAAGCAAATAGAATCACTATC
Sanger               CCTAATGGGAATAATGGGAAAACTATAAAGAAGCAAATAGAATCACTATC
Consensus            ***************** ********************************

Transcriptome NGS    CAAGTCTTTAGAAGCAGCAGCTCTAGAGGATGAAAGTGAATCCACCGGTG  1000
Viral RNA NGS        CAAGTCTTTAGAAGCAGCAGCTCTAGAGGATGAAAGTGAATCCACCGGTG
Sanger               CAAGTCTTTAGAAGCAGCAGCTCTAGAGGATGAAAGTGAATCCACCGGTG
Consensus            **************************************************
                                          NSm end ←┐
Transcriptome NGS    CTGAAATGAAAGAGTTAACCTTTGATCTTTAGACTGCTGTCATTCTGTTT  1050
Viral RNA NGS        CTGAAATGAAAGAGTTAACCTTTGATCTTTAGACTGCTGTCATTCTGTTT
Sanger               CTGAAATGAAAGAGTTAACCTTTGATCTTTAGACTGCTGTCATTCTGTTT
Consensus            **************************************************

Transcriptome NGS    TTATAAATTTGTTTGTTTTAGTATTTTTAAATCCAATAAATTTATGATTT  1100
Viral RNA NGS        TTATAAATTTGTTTGTTTTAGTATTTTTAAATCCAATAAATTTATGATTT
Sanger               TTATAAATTTGTTTGTTTTAGTATTTTTAAATCCAATAAATTTATGATTT
Consensus            **************************************************

Transcriptome NGS    GTACTTATGTATAAAATCAAAATACAAAATCAGAATTAAAAATCAAGTGC  1150
Viral RNA NGS        GTACTTATGTATAAAATCAAAATACAAAATCAGAATTAAAAATCAAGTGC
Sanger               GTACTTATGTATAAAATCAAAATACAAAATCAGAATTAAAAATCAAGTGC
Consensus            **************************************************

Transcriptome NGS    AACCAAAATTATAAAACAAAAACCTAAAAAACAAATAAACAAAAAACAAA  1200
Viral RNA NGS        AACCAAAATTATAAAACAAAAACCTAAAAAACAAATAAACAAAAAACAAA
Sanger               AACCAAAATTATAAAACAAAAACCTAAAAAACAAATAAACAAAAAACAAA
Consensus            **************************************************

Transcriptome NGS    AAACCAAAAAAANNNNNNNNNNNNNNNNNNNNNNNNNNNNNNNNNNNNNN  1250
Viral RNA NGS        AACCAAAAAAACCCGAANNNNNNNNNNNNNNNNNNNNNNNNNNNNNNNNN
Sanger               AACCAAAAAAACCCGAAAAAACCTAAAAAACCAAAAAACAAAAAACAAAA
Consensus            ** * ******

Transcriptome NGS    NNNNNNNNNNNNNNNNNNNNNAGGGAGAAATCCCCAAATTGGGACCTAATG  1300
Viral RNA NGS        NNNNNNNNNNNNNNNNNNNNNNNNNNNNNNNNNNNNNNNNNNNNNNNNNN
Sanger               AACCCAAAAAACCAAAAAAAGAGGGAGAAATCCCCAAATTGGGACCTAATG
Consensus

Transcriptome NGS    TCCTTTTATCATTTTTATATCTTTTATTTTGATTTTTGATTTTTTTGATT  1350
Viral RNA NGS        NNNNNNNNNNNNNNNNNNNNNNNNNNNNNNNNNNNNNNNNNNNNNNNNNN
Sanger               TCCTTTTATCATTTTTATATCTTTTATTTTGATTTTTGATTTTTTTGATT
Consensus

Transcriptome NGS    TTTTTGATTTGATTTTTGATTTTGATTCTTGGATCTTACATATTAGCATT  1400
Viral RNA NGS        NNNNNNNNNNNNNNNNNNNNNNNNNTTCTTGGATCTTACATATTAGCATT
Sanger               TTTTTGATTTGATTTTTGATTTTGATTCTTGGATCTTACATATTAGCATT
Consensus
```

```
Transcriptome NGS   AGTAACAACTAAATGATTTGATGTTGAAGATCTAGTTAAAAGATGTTTGA 1450
Viral RNA NGS       AGTAACAACTAAATGATTTGATGTTGAAGATCTAGTTAAAAGATGTTTGA
Sanger              AGTAACAACTAAATGATTTGATGTTGAAGATCTAGTTAAAAGATGTTTGA
Consensus           **************************************************

Transcriptome NGS   TTGAACTGTTTTGATCTACTAATTCATAAACCTGCGAGACTAACAATACT 1500
Viral RNA NGS       TTGAACTGTTTTGATCTACTAATTCATAAACCTGCGAGACTAACAATACT
Sanger              TTGAACTGTTTTGATCTACTAATTCATAAACCTGCGAAACTAACAATACT
Consensus           ************************************* ************

                          ┌→ Gn/Gc end
Transcriptome NGS   TGAAATTACAAATCAATGTATTTCAGCCTTCTCACAAGATTCTTTTCACC 1550
Viral RNA NGS       TGAAATTACAAATCAATGTATTTCAGCCTTCTCACAAGATTCTTTTCACC
Sanger              TGAAATTACAAATCTATGTATTTCAGCCTTCTCACAAGATTCTTTTCACC
Consensus           ************** ***********************************

Transcriptome NGS   ATACATTTCAGAATCGTCAACATCACTGTCATTGTCGTCATGTTTCTTCT 1600
Viral RNA NGS       ATACATTTCAGAATCGTCAACATCACTGTCATTGTCGTCATGTTTCTTCT
Sanger              ATACATTTCAGAATCGTCAACATCACTGTCATTGTCGTCATGTTTCTTCT
Consensus           **************************************************

Transcriptome NGS   TTTTGATATATATAGTTGCATGAGAGTAGGCTTCTTTCCCCAGGTTTACA 1650
Viral RNA NGS       TTTTGATATATATAGTTGCATGAGAGTAGGCTTCTTTCCCCAGGTTTACA
Sanger              TTTTGATATATATAGTTGCATGAGAGTAGGCTTCTTTCCCCAGGTTTACA
Consensus           **************************************************

Transcriptome NGS   CACTTTGATAAGACATATAAACCCAAAATTATGAATCCAAACAACAAGGC 1700
Viral RNA NGS       CACTTTGATAAGACATATAAACCCAAAATTATGAATCCAAACAACAAGGC
Sanger              CACTTTGATAAGACATATAAACCCAAAATTATGAATCCAAACAACAAGGC
Consensus           **************************************************

Transcriptome NGS   AAGTTTTAGCAAATCTAGTGCAGAGTTGGTGAAGTTGGCCACCCAATTAA 1750
Viral RNA NGS       AAGTTTTAGCAAATCTAGTGCAGAGTTGGTGAAGTTGGCCACCCAATTAA
Sanger              AAGTTTTAGCAAATCTAGTGCAGAGTTGGTGAAGTTGGCCACCCAATTAA
Consensus           **************************************************

Transcriptome NGS   AAGGGTAAGTCACAGTTTCTAAAAATGTGTGAAAGTCTGTATCTTCTTTA 1800
Viral RNA NGS       AAGGGTAAGTCACAGTTTCTAAAAATGTGTGAAAGTCTGTATCTTCTTTA
Sanger              AAGGGTAAGTCACAGTTTCTAAAAATGTGTGAAAGTCTGTATCTTCTTTA
Consensus           **************************************************

Transcriptome NGS   TGTTGGAAGCTTTTCTGGATCCTGTGATCCTTGAGAGTTTCTACATTCTT 1850
Viral RNA NGS       TGTTGGAAGCTTTTCTGGATCCTGTGATCCTTGAGAGTTTCTACATTCTT
Sanger              TGTTGGAAGCTTTTCTGGATCCTGTGATCCTTGAGAGTTTCTACATTCTT
Consensus           **************************************************

Transcriptome NGS   ATCGTTGATAATTTCTATCTTAGTCAAAGGGATATCTAAAGATATATTTT 1900
Viral RNA NGS       ATCGTTGATAATTTCTATCTTAGTCAAAGGGATATCTAAAGATATATTTT
Sanger              ATCGTTGATAATTTCTATCTTAGTCAAAGGGATATCTAAAGATATATTTT
Consensus           **************************************************

Transcriptome NGS   CATTGTCCTCCACAATCATCTTTAAAACAGCGTTTTCAGGAAGTACACCA 1950
Viral RNA NGS       CATTGTCCTCCACAATCATCTTTAAAACAGCGTTTTCAGGAAGTACACCA
Sanger              CATTGTCCTCCACAATCATCTTTAAAACAGCGTTTTCAGGAAGTACACCA
Consensus           **************************************************

Transcriptome NGS   CAATGCAAATTCATCGAAATCTCATTAGAACCTCTTTCTACAATCAAATA 2000
Viral RNA NGS       CAATGCAAATTCATCGAAATCTCATTAGAACCTCTTTCTACAATCAAATA
Sanger              CAATGCAAATTCATCGAAATCTCATTAGAACCTCTTTCTACAATCAAATA
Consensus           **************************************************

Transcriptome NGS   TTGGTGTTTAGACACACAAAAGTCTGAACTTATTCTAGATGCAAAATGTT 2050
Viral RNA NGS       TTGGTGTTTAGACACACAAAAGTCTGAACTTATTCTAGATGCAAAATGTT
Sanger              TTGGTGTTTAGACACACAAAAGTCTGAACTTATTCTAGATGCAAAATGTT
Consensus           **************************************************

Transcriptome NGS   TGTTACTATTGATTTTGTATTTACAATCTAGGCCTTTAAAACATTCTAAA 2100
Viral RNA NGS       TGTTACTATTGATTTTGTATTTACAATCTAGGCCTTTAAAACATTCTAAA
Sanger              TGTTACTATTGATTTTGTATTTACAATCTAGGCCTTTAAAACATTCTAAA
Consensus           **************************************************
```

```
Transcriptome NGS    CATCCTGAACAGAAATGATCTATGATGTTGACTTTAGGTGCTTCTGTTGT  2150
Viral RNA NGS        CATCCTGAACAGAAATGATCTATGATGTTGACTTTAGGTGCTTCTGTTGT
Sanger               CATCCTGAACAGAAATGATCTATGATGTTGACTTTAGGTGCTTCTGTTGT
Consensus            **************************************************

Transcriptome NGS    CTCAACAAAAAGCTCAATAGGCAGATCCAGAACAGTTTTGATTTTGCCCA  2200
Viral RNA NGS        CTCAACAAAAAGCTCAATAGGCAGATCCAGAACAGTTTTGATTTTGCCCA
Sanger               CTCAACAAAAAGCTCAATAGGCAGATCCAGAACAGTTTTGATTTTGCCCA
Consensus            **************************************************

Transcriptome NGS    AAGAATAATCTTTTAACATGGTTATCTTGTTTAGTCCTGTTATGACAAAG  2250
Viral RNA NGS        AAGAATAATCTTTTAACATGGTTATCTTGTTTAGTCCTGTTATGACAAAG
Sanger               AAGAATAATCTTTTAACATGGTTATCTCGTTTAGTCCTGTTATGACAAAG
Consensus            *************************** **********************

Transcriptome NGS    GATCCTTCATTAAGCTTCTCTAAACCAGAGAAGTGATCAAATGTGTAAAA  2300
Viral RNA NGS        GATCCTTCATTAAGCTTCTCTAGACCAGAGAAGTGATCAAATGTGTAAAA
Sanger               GATCCTTCATTAAGCTTCTCTAGACCAGAGAAGTGATCAAATGTGTAAAA
Consensus            ********************** ***************************

Transcriptome NGS    CCCGCATTTTTTAATATACACTTTCTTGGGTCCAATGAACTCACAGTCCC  2350
Viral RNA NGS        CCCGCATTTTTTAATATACACTTTCTTGGGTCCAATGAACTCACAGTCCC
Sanger               CCCGCATTTTTTAATATACACTTTCTTGGGTCCAATGAACTCACAGTCCC
Consensus            **************************************************

Transcriptome NGS    ATGACAGCTGTTCTTTGCCTATGTATTCTTCTGCAAATAGTAATTCTCCT  2400
Viral RNA NGS        ATGACAGCTGTTCTTTGCCTATGTATTCTTCTGCAAATAGTAATTCTCCT
Sanger               ATGACAGCTGTTCTTTGCCTATGTATTCTTCTGCAAATAGTAATTCTCCT
Consensus            **************************************************

Transcriptome NGS    TGCTTGTTCAGTTGAGGATGACCAAACGTCATGTCTGCACTCATAAGATC  2450
Viral RNA NGS        TGCTTGTTCAGTTGAGGATGACCAAACGTCATGTCTGCACTCATAAGATC
Sanger               TGTTTGTTCAGTTGAGGATGACCAAACGTCATGTCTGCACTCATAAGATC
Consensus            ** ***********************************************

Transcriptome NGS    AGCTATGTTTCCTGTGTAAGCAGAGCCATCTTTAGCTAATGCAAGTTTGT  2500
Viral RNA NGS        AGCTATGTTTCCTGTGTAAGCAGAGCCATCTTTAGCTAATGCAAGTTTGT
Sanger               AGCTATGTTTCCTGTGTAAGCAGAGCCATCTTTAGCTAATGCAAGTTTGT
Consensus            **************************************************

Transcriptome NGS    GGCCAACAGAAATGTAATCATTCTGCAGAGTAGATGTTATTGATACTTGA  2550
Viral RNA NGS        GGCCAACAGAAATGTAATCATTCTGCAGAGTAGATGTTATTGATACTTGA
Sanger               GGCCAACAGAAATGTAATCATTCTGCAGAGTAGATGTTATTGATACTTGA
Consensus            **************************************************

Transcriptome NGS    TAGTTATCATTAGTGTAAGCTTTCCTATCATCATGATTGAAGCAATTATA  2600
Viral RNA NGS        TAGTTATCATTAGTGTAAGCTTTCCTATCATCATGATTGAAGCAATTATA
Sanger               TAGTTATCATTAGTGTAAGCTTTCCTATCATCATGATTGAAGCAATTATA
Consensus            **************************************************

Transcriptome NGS    CCCTTTGCCTGCATTTGCACAGGCTGTTGATGAAACATGGACCTCCTTAA  2650
Viral RNA NGS        CCCTTTGCCTGCATTTACACAGGCTGTTGATGAAACATGGACCTCCTTAA
Sanger               CCCTTTGCCTGCATTTACACAGGCTGTTGATGAAACATGGACCTCCTTAA
Consensus            **************** *********************************

Transcriptome NGS    CCCTGTACACATCTAAAGAATTGCTCATATCATAAACATTATCACAGTGA  2700
Viral RNA NGS        CCCTGTACACATCTAAAGAATTGCTCATATCATAAACATTATCACAGTGA
Sanger               CCCTGTACACATCTAAAGAATTGCTCATATCATAAACATTATCACAGTGA
Consensus            **************************************************

Transcriptome NGS    CCACAAGTTGAGCCTTCATTCATAGCTATGCACATAAGATCTTCACAGCC  2750
Viral RNA NGS        CCACAAGTTGAGCCTTCATTCATAGCTATGCACATAAAATCTTCACAGCC
Sanger               CCACAAGTTGAGCCTTCATTCATAGCTATGCACATAAAATCTTCACAGCC
Consensus            ************************************* ************

Transcriptome NGS    CCATTGGGATGTAGGTTCTAGACAAAATTTATGATATGATGGATTGTCTG  2800
Viral RNA NGS        CCATTGGGATGTAGGTTCTAGACAAAATTTATGATATGATGGATTGTCTG
Sanger               CCATTGGGATGTAGGTTCTAGACAAAATTTATGATATGATGGATTGTCTG
Consensus            **************************************************
```

```
Transcriptome NGS    TGGATCCTAAACCTTCCCAGCATTTTTCACAATTTCCAGTGCAAGAAGAA  2850
Viral RNA NGS        TGGATCCTAAACCTTCCCAGCATTTTTCACAATTTCCAGTGCAAGAAGAA
Sanger               TGGATCCTAAACCTTCCCAGCATTTTTCACAATTTCCAGTGCAAGAAGAA
Consensus            **************************************************

Transcriptome NGS    TAAATAGATGTTTTTGTAGTCACAATGTCAGCAGTTGTGTAGAGATATTC  2900
Viral RNA NGS        TAAATAGATGTTTTTGTAGTCACAATGTCAGCAGTTGTGTAGAGATATTC
Sanger               TAAATAGATGTTTTTGTAGCCACAATGTCAGCAGTTGTGTAGAGATATTC
Consensus            ******************* ******************************

Transcriptome NGS    AATAGTATAAGTGACTCCAACATTTTTGTGTGTGATCATCAATTTTTTTG  2950
Viral RNA NGS        AATAGTATAAGTGACTCCAACATTTTTGTGTGTGATCATCAATTTTTTTG
Sanger               AATAGTATAAGTGACTCCAACATTTTTGTGTGTGATCATCAATTTTTTTG
Consensus            **************************************************

Transcriptome NGS    AATGGCTCATTGTTCCGTCAACCATCTCAAACACAGTCCCTGTGTCTGAT  3000
Viral RNA NGS        AATGGCTCATTGTTCCGTCAACCATCTCAAACACAGTCCCTGTGTCTGAT
Sanger               AATGGCTCATTGTTCCGTCAACCATCTCAAACACAGTCCCTGTGTCTGAT
Consensus            **************************************************

Transcriptome NGS    ATCAAGTCTTGCTCTATCATGTAGCGGTATTTCCCATCTACTTTAGAAGA  3050
Viral RNA NGS        ATCAAGTCTTGCTCTATCATGTAGCGGTATTTCCCATCTACTTTAGAAGA
Sanger               ATCAAGTCTTGCTCTATCATGTAGCGGTATTTCCCATCTACTTTAGAAGA
Consensus            **************************************************

Transcriptome NGS    ATACATGAGAGTAGTTCGAGACAAGGTGTTACTATCTGAAATGCTGGACA  3100
Viral RNA NGS        ATACATGAGAGTAGTTCGAGACAAGGTGTTACTATCTGAAATGCTGGACA
Sanger               ATACATGAGAGTAGTTCGAGACAAGGTGTTACTATCTGAAATGCTGGACA
Consensus            **************************************************

Transcriptome NGS    CATCTAACTTGTCTAGTGAAGCTATAGCCTTCGACATCACAGAATTAGTT  3150
Viral RNA NGS        CATCTAACTTGTCTAGTGAAGCTATAGCCTTCGACATCACAGAATTAGTT
Sanger               CATCTAACTTGTCTAGTGAAGCTATAGCCTTCGACATCACAGAATTAGTT
Consensus            **************************************************

Transcriptome NGS    TTTTCCCAGGTGCTCAAAGATGGCTCTTGTCTTGATAAAATAGTCCTGTT  3200
Viral RNA NGS        TTTTCCCAGGTGCTCAAAGATGGCTCTTGTCTTGATAAAATAGTCCTGTT
Sanger               TTTTCCCAGGTGCTCAAAGATGGCTCTTGTCTTGATAAAATAGTCCTGTT
Consensus            **************************************************

Transcriptome NGS    GTCTATAACCCCTGTTCTGCTCTTCTGGAGGGAATACAGGTCTATAGGTT  3250
Viral RNA NGS        GTCTATAACCCCTGTTCTGCTCTTCTGGAGGGAATACAGGTCTATAGGTT
Sanger               GTCTATAACCCCTGTTCTGCTCTTCTGGAGGGAATACAGGTCTATAGGTT
Consensus            **************************************************

Transcriptome NGS    TCATTTTCCCGTAAGCCTCACCTTTATAGCTTTCATGAACACTAGGCAAT  3300
Viral RNA NGS        TCATTTTCCCGTAAGCCTCACCTTTATAGCTTTCATGAACACTAGGCAAT
Sanger               TCATTTTCCCGTAAGCCTCACCTTTATAGCTTTCATGAACACTAGGCAAT
Consensus            **************************************************

Transcriptome NGS    GGTTCATATATGTACATCTTTAAAGCTCCACAACTATAAGCACAAGCTAA  3350
Viral RNA NGS        GGTTCATATATGTACATCTTTAAAGCTTCACAACTATAAGCACAAGCTAA
Sanger               GGTTCATATATGTACATCTTTAAAGCTTCACAACTATAAGCACAAGCTAA
Consensus            *************************** **********************

Transcriptome NGS    GATTCTCAAAACTTCTGTATCTAAGTGGTCCGCACAATCGTCGTCGAAGT  3400
Viral RNA NGS        GATTCTCAAAACTTCTGTATCTAAGTGGTCCGCACAATCGTCGTCGAAGT
Sanger               GATTCTCAAAACTTCTGTATCTAAGTGGTCCGCACAATCGTCGTCGAAGT
Consensus            **************************************************

Transcriptome NGS    CAGGATAACACTTTTTATTAGCTATATTTGTCTGTGTTTTCCCAGTTATG  3450
Viral RNA NGS        CAGGATAACACTTTTTATTAGCTATATTTGTCTGTGTTTTCCCAGTTATG
Sanger               CAGGATAACACTTTTTATTAGCTATATTTGTCTGTGTTTTCCCAGTTATG
Consensus            **************************************************

Transcriptome NGS    TCTTTATAAGTCTCTACAACTTTGAAGGGGAAATCTAAGCTACCAGAGTC  3500
Viral RNA NGS        TCTTTATAAGTCTCTACAACTTTGAAGGGGAAATCTAAGCTACCAGAGTC
Sanger               TCTTTATAAGTCTCTACAACTTTGAAGGGGAAATCTAAGCTACCAGAGTC
Consensus            **************************************************
```

```
Transcriptome NGS   TTTTGTGATTTTACAATCACATTCTCGGGCAGAAGGCATAGAATGGGATC  3550
Viral RNA NGS       TTTTGTGATTTTACAATCACATTCTCGGGCAGAAGGCATAGAATGGGATC
Sanger              TTTTGTGATTTTACAATCACATTCTCGGGCAGAAGGCATAGAATGGGATC
Consensus           **************************************************

Transcriptome NGS   CAATGTACAAGAATTTTGACTGCTCGAGATCAGCATAACAATTATAGCCA  3600
Viral RNA NGS       CAATGTACAAGAATTTTGACTGCTCGAGATCAGCATAACAATTATAGCCA
Sanger              CAATGTACAAGAATTTTGACTGCTCGAGATCAGCATAACAATTATAGCCA
Consensus           **************************************************

Transcriptome NGS   CAGGATGCTTGCAGAGCTAAAGTGTTTGGTAGAATTGAAATAACAATTGT  3650
Viral RNA NGS       CAGGATGCTTGCAGAGCTAAAGTGTTTGGTAGAATTGAAATAACAATTGT
Sanger              CAGGATGCTTGCAGAGCTAAAGTGTTTGGTAGAATTGAAATAACAATTGT
Consensus           **************************************************

Transcriptome NGS   GCTAAGCATGAATCTCGTTAGATAAAATGTGACATTTCTAGTAATACCCG  3700
Viral RNA NGS       GCTAAGCATGAATCTCGTTAGATAAAATGTGACATTTCTAGTAATACCCG
Sanger              GCTAAGCATGAATCTCGTTAGATAAAATGTGACATTTCTAGTAATACCCG
Consensus           **************************************************

Transcriptome NGS   TATTCATCAAAAATTGAAATTTTTTAGATGGTTTTTTGGGAAATCCGCGG  3750
Viral RNA NGS       TATTCATCAAAAATTGAAATTTTTTAGATGGTTTTTTGGGAAATCCGCGG
Sanger              TATTCATCAAAAATTGAAATTTTTTAGATGGTTTTTTGGGAAATCCGCGG
Consensus           **************************************************

Transcriptome NGS   TTAAAGTAACAATCAGGATTATGCAAAGCTCCAGAATCTGATCTACCACA  3800
Viral RNA NGS       TTAAAGTAACAATCAGGATTATGCAAAGCTCCAGAATCTGATCTACCACA
Sanger              TTAAAGTAACAATCAGGATTATGCAAAGCTCCAGAATCTGATCTACCACA
Consensus           **************************************************

Transcriptome NGS   AACACACTTTGGTGGACAATCATGAGACAGCAGAGACAAATATCCACACA  3850
Viral RNA NGS       AACACACTTTGGTGGACAATCATGAGACAGCAGAGACAAATATCCACACA
Sanger              AACACACTTTGGTGGACAATCATGAGACAGCAGAGACAAATATCCACACA
Consensus           **************************************************

Transcriptome NGS   TATCACATCTATATGGCATTTTAGACCATAAATTGTTTAACAGGTAAATC  3900
Viral RNA NGS       TATCACATCTATATGGCATTTTAGACCATAAATTGTTTAACAGGTAAATC
Sanger              TATCACATCTATATGGCATTTTAGACCATAAATTGTTTAACAGGTAAATC
Consensus           **************************************************

Transcriptome NGS   AGAGGATACAAAACTATAGAAAGCAAATCATACCATAGGGATAGTGGACC  3950
Viral RNA NGS       AGAGGATACAAAACTATAGAAAGCAAATCATACCATAGGGATAGTGGACC
Sanger              AGAGGATACAAGACTATAGAAAGCAAATCATACCATAGGGATAGTGGACC
Consensus           *********** **************************************

Transcriptome NGS   TTTGGTTTTGTTCATGATCCAGGTTATTGGCATCATTATTAGGAAGAATA  4000
Viral RNA NGS       TTTGGTTTTGTTCATGATCCAGGTTATTGGCATCATTATTAGGAAGAATA
Sanger              TTTGGTTTTGTTCATGATCCAGGTTATTGGCATCATTATTAGGAAGAATA
Consensus           **************************************************

Transcriptome NGS   AAAATGCCCATTTCAACCAATAAAAATTCACACAAGTATACAACATTGTC  4050
Viral RNA NGS       AAAATGCCCATTTCAACCAATAAAAATTCACACAAGTATACAACATTGTC
Sanger              AAAATGCCCATTTCAACCAATAAAAATTCACACAAGTATACAACATTGTC
Consensus           **************************************************

Transcriptome NGS   TTATTATCTTGATACTTATGGACACACCTTCGAAGACCATTGTCGACATT  4100
Viral RNA NGS       TTATTATCTTGATACTTATGGACACACCTTCGAAGACCATTGTCGACATT
Sanger              TTATTATCTTGATACTTATGGACACACCTTCGAAGACCATTGTCGACATT
Consensus           **************************************************

Transcriptome NGS   AGGTAAGTGGAAGAACTTATCTCCGCATATTAGGTAATGAAACCCGTCTA  4150
Viral RNA NGS       AGGTAAGTGGAAGAACTTATCTCCGCATATTAGGTAATGAAACCCGTCTA
Sanger              AGGTAAGTGGAAGAACTTATCTCCGCATATTAGGTAATGAAACCCGTCTA
Consensus           **************************************************

Transcriptome NGS   AGGAATCCTCAGACAACTGATATTGGAATGTTCCTGTAAAAGATTGAACC  4200
Viral RNA NGS       AGGAATCCTCAGACAACTGATATTGGAATGTTCCTGTAAAAGATTGAACC
Sanger              AGGAATCCTCAGACAACTGATATTGGAATGTTCCTGTAAAAGATTGAACC
Consensus           **************************************************
```

```
Transcriptome NGS   TTAGAGTCAAGCTGACCCATTTGTTTCACAGTGACTCCAATCAATTTTTC  4250
Viral RNA NGS       TTAGAGTCAAGCTGACCCATTTGTTTCACAGTGACTCCAATCAATTTTTC
Sanger              TTAGAGTCAAGCTGACCCATTTGTTTCACAGTGACTCCAATCAATTTTTC
Consensus           **************************************************

Transcriptome NGS   TGTGGTTTCCAAGTTGACATTAAAAGTCTCATCACTATTCAACTGCGAGA  4300
Viral RNA NGS       TGTGGTTTCCAAGTTGACATTAAAAGTCTCATCACTATTCAACTGCGAGA
Sanger              TGTGGTTTCCAAGTTGACATTAAAAGTCTCATCACTATTCAACTGCGAGA
Consensus           **************************************************

Transcriptome NGS   TATTGCAATCACCTGATAACTTCACAGAGTGTAAAACAATATGCTGCTTT  4350
Viral RNA NGS       TATTGCAATCACCTGATAACTTCACAGAGTGTAAAACAATATGCTGCTTT
Sanger              TATTGCAATCACCTGATAACTTCACAGAGTGTAAAACAATATGCTGCTTT
Consensus           **************************************************

Transcriptome NGS   GAACCGATTTTCTCTTCGAAGTATTTACTACCTACTTCAAGTAGCCTTTT  4400
Viral RNA NGS       GAACCGATTTTCTCTTCGAAGTATTTACTACCTACTTCAAGTAGCCTTTT
Sanger              GAACCGATTTTCTCTTCGAAGTATTTACTACCTACTTCAAGTAGCCTTTT
Consensus           **************************************************

Transcriptome NGS   GTTTTCCACATTCATGACAGGGACCACTGGAATTGTAATTTCAGAATTGA  4450
Viral RNA NGS       GTTTTCCACATTCATGACAGGGACCACTGGAATTGTAATTTCAGAATTGA
Sanger              GTTTTCCACATTCATGACAGGGACCACTGGAATTGTAATTTCAGAATTGA
Consensus           **************************************************

Transcriptome NGS   AATCTGAAAGAAGATTAATGTTTTTGCATTTTTTATAGAATGTAACGTTT  4500
Viral RNA NGS       AATCTGAAAGAAGATTAATGTTTTTGCATTTTTTATAGAATGTAACGTTT
Sanger              AATCTGAAAGAAGATTAATGTTTTTGCATTTTTTATAGAATGTAACGTTT
Consensus           **************************************************

Transcriptome NGS   TCTGAAGGACATGTATAATAATCATCTCCTTCATTCATTGTTGCAAGAAA  4550
Viral RNA NGS       TCTGAAGGACATGTATAATAATCATCTCCTTCATTCATTGTTGCAAGAAA
Sanger              TCTGAAGGACATGTATAATAATCATCTCCTTCATTCATTGTTGCAAGAAA
Consensus           **************************************************

Transcriptome NGS   GTTGAAAGTGGATTTCCCCACTATCATACAGTTAGATGGCAATGGAGGTT  4600
Viral RNA NGS       GTTGAAAGTGGATTTCCCCACTATCATACAGTTAGATGGCAATGGAGGTT
Sanger              GTTGAAAGTGGATTTCCCCACTATCATACAGTTAGATGGCAATGGAGGTT
Consensus           **************************************************

Transcriptome NGS   CATGATTTTGCTCAGTGTACCTGCATTTTTCTTTATCTGTATAACTATCA  4650
Viral RNA NGS       CATGATTTTGCTCAGTGTACCTGCATTTTTCTTTATCTGTATAACTATCA
Sanger              CATGATTTTGCTCAGTGTACCTGCATTTTTCTTTATCTGTATAACTATCA
Consensus           **************************************************

Transcriptome NGS   GACAGCATCACACTCCTATATGTCCCAGAGTATTTCTTCTTCCCAGACTG  4700
Viral RNA NGS       GACAGCATCACACTCCTATATGTCCCAGAGTATTTCTTCTTTCCAGACTG
Sanger              GACAGCATCACACTCCTATATGTCCCAGAGTATTTCTTCTTTCCAAACTG
Consensus           ***************************************** *** ****

Transcriptome NGS   GCTTTCGAGGCTTGGTGACTTCTCTGAACTGGACAATTGGCGGAGATTGA  4750
Viral RNA NGS       GCTTTCGAGGCTTGGTGACTTCTCTGAACTGGACAATTGGCGGAGATTGA
Sanger              GCTTTCGAGGCTTGGTGACTTCTCTGAACTGGACAATTGGCGGAGATTGA
Consensus           **************************************************

Transcriptome NGS   GGATTAGACAAAGCATGAGTATCATTATTACTAGCAATGATGTGACATAA  4800
Viral RNA NGS       GGATTAGACAAAGCATGAGTATCATTATTACTAGCAATGATGTGACATAA
Sanger              GGATTAGACAAAGCATGAGTATCATTATTACTAGCAATGATGTGACATAA
Consensus           **************************************************

Transcriptome NGS   AATATAAAGCCAGATAAGTAAATAGGTAAGCTTGATGACATTTTTTTACG  4850
Viral RNA NGS       AATATAAAGCCAGATAAGTAAATAGGTAAGCTTGATGACATTTTTT-ACG
Sanger              AATATAAAGCCAGATAAGTAAATAGGTAAGCTTGATGACATTTTTT-ACG
Consensus           ********************************************** ***
                          Gn/Gc <-┐
Transcriptome NGS   GTTTAGTAGAGTCATTTCAAGGGTTATTCTCGTGTGCCGATTGCTC----  4900
Viral RNA NGS       GTTTAGTAGAGTCTTTTCAAGGGTTATTCTCGTGTGCCGATTGCTCTATT
Sanger              GTTTAGTAGAGTCATTTCAAGGGTTATTCTCGTGTGCCGATTGCTCT---
Consensus           ************* ********************************
```

```
Transcriptome NGS   --------------------------                     4950
Viral RNA NGS       AAACCGTAAAAAATGTCATCAAGCT
Sanger              --------------------------
Consensus
```

S3 Fig. Comparison of the sequences of the Groundnut chlorotic fan-spot virus (GCFSV) M RNA as determined by next-generation sequencing (NGS) and Sanger sequencing methods. The nucleotide sequences from the transcriptome and viral RNA sequenced by RNA-seq are aligned with those sequenced by the Sanger method for comparison. The underlines indicate the nucleotides that are lost in the obtained sequences. The bold characters represent the nucleotides that are diverse among the three sequences.
