# Supplementary material for: Characterization of the genome of a phylogenetically distinct tospovirus and its interactions with the local lesion-induced host Chenopodium quinoa by whole-transcriptome analyses
Source: PLoS One. 2017 Aug 3;12(8):e0182425. doi: 10.1371/journal.pone.0182425 (PMC5542687; doi:10.1371/journal.pone.0182425)
Supplement: S4 Fig — The nucleotide sequences from the transcriptome and viral RNA sequenced by RNA-seq are aligned with those sequenced by the Sanger method for comparison. The underlines indicate the nucleotides that are lost in the obtained sequences. The bold characters represent the nucleotides that are diverse among the three sequences. (PDF) [file pone.0182425.s004.pdf]

```
Transcriptome NGS   --------------------------------------------------   50
Viral RNA NGS       --------------------------------------------------
Sanger              AGAGCAATCAGGCAACAAGAAAAATTCAAATTGAATATTAAGTAAAACTT
Consensus

Transcriptome NGS   --------------------------------------------------   100
Viral RNA NGS       -----------ACATAATAAACTATCATGTCAAGTAATAACAAGGGTCTC
Sanger              CAAGCTATCATACATAATAAACTATCATGTCAAGTAATAACAAGGGACTC
Consensus

                               → RdRp end
Transcriptome NGS   --------------------------------------------------   150
Viral RNA NGS       TAGCAAATCATCTAACTGATACCTAGCAAATCATCTAACTGATACCTAAG
Sanger              TAGCAAATCATCTAACTGATACCTAGCAAATCATCTAACTGATACCTAAG
Consensus

Transcriptome NGS   --------------------------------------------------   200
Viral RNA NGS       AATTTGGAGATCCTCCTAATAGTTGTTTTTTCTCCCATTTTGATTTCCTT
Sanger              AATTTGGAGATCCTCCTAATAGTTGTTTTTTCTCCCATTTTGATTTCCTT
Consensus

Transcriptome NGS   --------------------------------------------------   250
Viral RNA NGS       CTGCTTGTACATCAGAGTCTTACATGAAGTGATGATAGAATCAATCGTCT
Sanger              CTGCTTGTACATCAGAGTCTTACATGAAGTGATGATAGAATCAATCGTCT
Consensus

Transcriptome NGS   --------------------------------------------------   300
Viral RNA NGS       TATTTCTTGCATCCATGAACATGAAAGAATCTTTAAAAATTGAGCTCTTA
Sanger              TATTTCTTGCATCCATGAACATGAAAGAATCTTTAAAAATTGAGCTCTTA
Consensus

Transcriptome NGS   --------------------------------------------------   350
Viral RNA NGS       TTGCTGATGTAGTCTATAAACTTATTCATCTCTAACACATCTTCTGCTTT
Sanger              TTGCTGATGTAGTCTATAAACTTATTCATCTCTAACACATCTTCTGCTTT
Consensus

Transcriptome NGS   --------------------------------------------------   400
Viral RNA NGS       GAATATTTTAGTCATAATGATATCAATATCTCCTATTTCATCATCATCAT
Sanger              GAATATTTTAGTCATAATGATATCAATATCTCCTATTTCATCATCATCAT
Consensus

Transcriptome NGS   --------------------------------------------------   450
Viral RNA NGS       AATCATAGATTTTATTAGACGAAATTTCAATTTTGAGGATGCCTAGAAGA
Sanger              AATCATAGATTTTATTAGACGAAATTTCAATTTTGAGGATGCCTAGAAGA
Consensus

Transcriptome NGS   --------------------------------------------------   500
Viral RNA NGS       TTGTTTATACTGTTACTCAAATTCAACATGATGGTAGCATCTTTATTCTT
Sanger              TTGTTTATACTGTTACTCAAATTCAACATGATGGTAGCATCTTTATTCTT
Consensus

Transcriptome NGS   --------------------------------------------------   550
Viral RNA NGS       TGACTTTTTAAATAGAATTTTAGACAGAACAGAGCATAACCCAGAAAATG
Sanger              TGACTTTTTAAATAGAATTTTAGACAGAACAGAGCATAACCCAGAAAATG
Consensus

Transcriptome NGS   --------------------------------------------------   600
Viral RNA NGS       ATATTAGGGAAAACAACCTTGGACTAGTTCTGCCTTTCTTTTCAGCATTG
Sanger              ATATTAGGGAAAACAACCTTGGACTAGTTCTGCCTTTCTTTTCAGCATTG
Consensus

Transcriptome NGS   --------------------------------------------------   650
Viral RNA NGS       TCTGAAAACATTCTTCTTATTATTACTTCCATGTCTTCCTCTGTCCCATC
Sanger              TCTGAAAACATTCTTCTTATTATTACTTCCATGTCTTCCTCTGTCCCATC
Consensus

Transcriptome NGS   --------------------------------------------------   700
Viral RNA NGS       AACAGTCATGTATATAGTTTTCAGAATTTCTGACAATAGAAGAGTGTTAG
Sanger              AACAGTCATGTATATAGTTTTCAGAATTTCTGACAATAGGAGAGTGTTAG
Consensus
```

```
Transcriptome NGS   --------------------------------------------------  750
Viral RNA NGS       AAGGTATCAGGTGACCATCTTTCTTTGTTTTAAATTTTGCGTTCCTGCAA
Sanger              AAGGTATCAGGTGACCATCTTTCTTTGTTTTAAATTTTGCGTTCCTGCAA
Consensus

Transcriptome NGS   --------------------------------------------------  800
Viral RNA NGS       ATATAACTATAAACTTCTTTATCATTATATAATTCAGGGTCTTCTTCTAT
Sanger              ATATAACTATAAACTTCTTTATCATTATATAATTCAGGGTCTTCTTCTAT
Consensus

Transcriptome NGS   --------------------------------------------------  850
Viral RNA NGS       CCCAGCTATCATAGGATTTTCTTTAAGAATGCTGTTCGTTTCAACTTTGA
Sanger              CCCAGCTATCATAGGATTTTCTTTAAGAATGCTGTTCGTTTCAACTTTGA
Consensus

Transcriptome NGS   --------------------------------------------------  900
Viral RNA NGS       AATTTTGTTCTCTTAGTAGTTTCTTTGCAACTTCATTCAATTTTATATCA
Sanger              AATTTTGTTCTCTTAGTAGTTTCTTTGCAACTTCATTCAATTTTATATCA
Consensus

Transcriptome NGS   --------------------------------------------------  950
Viral RNA NGS       CCTAGAGATAAAGTTTTGTAATCTGATACACTCTTCATATTGCTATGATT
Sanger              CCTAGAGATAAAGTTTTGTAATCTGATACACTCTTCATATTGCTATGATT
Consensus

Transcriptome NGS   --------------------------------------------------  1000
Viral RNA NGS       TAGATCCATATCATGTTTTATTGACATAGAGTTAGGAGATAAGAACAATT
Sanger              TAGATCCATATCATGTTTTATTGACATAGAGTTAGGAGATAAGAACAATT
Consensus

Transcriptome NGS   --------------------------------------------------  1050
Viral RNA NGS       GGTTTTTTATCTGACTAAAGAAAGACAAAGACAAAACTGCTCCATTATTA
Sanger              GGTTTTTTATCTGACTAAAGAAAGACAAAGACAAAACTGCTCCATTATTA
Consensus

Transcriptome NGS   --------------------------------------------------  1100
Viral RNA NGS       TGACAGATTTCAGCCATTTTCACCAATCTTAGGATGCCTAACACGTCATA
Sanger              TGACAGATTTCAGCCATTTTCACCAATCTTAGGATGCCTAACACGTCATA
Consensus

Transcriptome NGS   --------------------------------------------------  1150
Viral RNA NGS       TTTTCCTATTACGCAATTTTCCCTTTTTATCAACTCATTTATGCTTCTAC
Sanger              TTTTCCTATTACGCAATTTTCCCTTTTTATCAACTCATTTATGCTTCTAC
Consensus

Transcriptome NGS   --------------------------------------------------  1200
Viral RNA NGS       CAGTTGGTCTTGGTGTTCTAAGAGCATGCTCATCATTCTTTGATAGAAAA
Sanger              CAGTTGGTCTTGGTGTTCTAAGAGCATGCTCATCATTCTTTGATAGAAAA
Consensus

Transcriptome NGS   --------------------------------------------------  1250
Viral RNA NGS       GTTAAGACATTCATAGCAGTTTCTAGGTTCAATAATTTTGATGTATAAAG
Sanger              GTTAAGACATTCATAGCAGTTTCTAGGTTCAATAATTTTGATGTATAAAG
Consensus

Transcriptome NGS   --------------------------------------------------  1300
Viral RNA NGS       TTGTCTATAATTCACATCATCTTCGGGTTCTATTATCAAACTTTCATCCA
Sanger              TTGTCTATAATTCACATCATCTTCGGGTTCTATTATCAAACTTTCATCCA
Consensus

Transcriptome NGS   --------------------------------------------------  1350
Viral RNA NGS       TCATGGACGATAACATTGTTAATTCAGACTCAATTTTTCCTACTAGTTCC
Sanger              TCATGGACGATAACATTGTTAATTCAGACTCAATTTTTCCTACTAGTTCC
Consensus

Transcriptome NGS   --------------------------------------------------  1400
Viral RNA NGS       TCTACTTTTGAAATGTCTGGATTACTCATAAATTCATTTACACTAGCTAT
Sanger              TCTACTTTTGAAATGTCTGGATTACTCATAAATTCATTTACACTAGCTAT
Consensus
```

```
Transcriptome NGS    --------------------------------------------------  1450
Viral RNA NGS        TATGCTATCGTTTAAGCTTTCTATCATCATCTCATCATCTTCAGTCAGAT
Sanger               TATGCTATCGTTTAAGCTTTCTATCATCATCTCATCATCTTCAGTCAGAT
Consensus

Transcriptome NGS    --------------------------------------------------  1500
Viral RNA NGS        CAGAGCCATCTTTGACTATTTTAACTAATCCAGTGACATGGTTGATAAAA
Sanger               CAGAGCCATTTTTGACTATTTTAACTAATCCAGTGACATGGTTGATAAAA
Consensus

Transcriptome NGS    --------------------------------------------------  1550
Viral RNA NGS        TTTAGCGTCTCAACAAGCCTTTTCAAAGCCACATCAAAGAGAGTCTTTTC
Sanger               TTTAGCGTCTCAACAAGCCTTTTCAAAGCCACATCAAAGAGAGTCTTTTC
Consensus

Transcriptome NGS    --------------------------------------------------  1600
Viral RNA NGS        AGGTACACTCACATATTTATACAGATTGGTCATAGGTTCTCCAACTTTGG
Sanger               AGGTACACTCACATATTTATACAGATTGGTCATAGGTTCTCCAACTTTGG
Consensus

Transcriptome NGS    --------------------------------------------------  1650
Viral RNA NGS        GATATCGGGGTCTAATATTGTCATTGCTTTCAAAAGTGTCCCACACTTCT
Sanger               GATATCGGGGTCTAATATTGTCATTGCTTTCAAAAGTGTCCCACACTTCT
Consensus

Transcriptome NGS    --------------------------------------------------  1700
Viral RNA NGS        TTTTTTATTTTGTTGTACTCTATTTTGCAACCCATTTTCACATTAGTCAG
Sanger               TTTTTTATTTTGTTGTACTCTATTTTGCAACCCATTTTCACATTAGTCAG
Consensus

Transcriptome NGS    --------------------------------------------------  1750
Viral RNA NGS        ATAGCAAGGGTTATCGACAATTGTTGAGAAAAGGTCATTCCTGGAACTTA
Sanger               ATAGCAAGGGTTATCGACAATTGTTGAGAAAAGGTCATTCCTGGAACTTA
Consensus

Transcriptome NGS    --------------------------------------------------  1800
Viral RNA NGS        GTTTAATGTATGCATGATATGGTGATGCAACAAACTCAGAAGAACTTGCT
Sanger               GTTTAATGTATGCATGATATGGTGATGCAACAAACTCAGAAGAACTTGCT
Consensus

Transcriptome NGS    --------------------------------------------------  1850
Viral RNA NGS        AGTTTTATAACTTCTTTCTTATCTCTTATAAATTTAGACAACAACTTGTT
Sanger               AGTTTTATAACTTCTTTCTTATCTCTTATAAATTTAGACAACAACTTGTT
Consensus

Transcriptome NGS    --------------------------------------------------  1900
Viral RNA NGS        TATGTCTTCTTTAGCAGAGTTGGTTATTTTCTCTTCAAAAATCATGTCTT
Sanger               TATGTCTTCTTTAGCAGAGTTGGTTATTTTCTCTTCAAAAATCATGTCTT
Consensus

Transcriptome NGS    --------------------------------------------------  1950
Viral RNA NGS        TTATGGATTGAACCTCTATCTTCAAGATGTTTCTATTCCCTTGTTTTGAT
Sanger               TTATGGATTGAACCTCTATCTTCAAGATGTTTCTATTCCCTTGTTTTGAT
Consensus

Transcriptome NGS    --------------------------------------------------  2000
Viral RNA NGS        GAGTATCTTAGAGTAGTATAATAATGATCAGATTTGGTTGCAATGCAATA
Sanger               GAGTATCTTAGAGTAGTATAATAATGATCAGATTTGGTTGCAATGCAATA
Consensus

Transcriptome NGS    --------------------------------------------------  2050
Viral RNA NGS        TGTTGAATCATTATCATAGTTTCCTTCCCCATCAACGGATTCAGCTTTAA
Sanger               TGTTGAATCATTATCATAGTTTCCTTCCCCATCAACGGATTCAGCTTTAA
Consensus

Transcriptome NGS    --------------------------------------------------  2100
Viral RNA NGS        TATAGACAACATCTGCTTTAGATCCGTATTTATAGAGAAAACTGAATGTT
Sanger               TATAGACAACATCTGCTTTAGATCCGTATTTATAGAGAAAACTGAATGTT
Consensus
```

```
Transcriptome NGS   --------------------------------------------------   2150
Viral RNA NGS       CTGTAGTCTATGAAATTCATGTTGTAAAGCCATGCAATAGACCTAGATAA
Sanger              CTGTAGTCTATGAAATTCATGTTGTAAAGCCATGCAATAGACCTAGATAA
Consensus

Transcriptome NGS   --------------------------------------------------   2200
Viral RNA NGS       CATCCTTATCTTCATTTCATGTTTACTTCTCAAGAGATGGGTGGTTTTTA
Sanger              CATCCTTATCTTCATTTCATGTTTACTTCTCAAGAGATGGGTGGTTTTTA
Consensus

Transcriptome NGS   --------------------------------------------------   2250
Viral RNA NGS       TTAAAACAAGATCTTTCTGTAACTCATCACATGCAGTTTGTATATATTCA
Sanger              TTAAAACAAGATCTTTCTGTAACTCATCACATGCAGTTTGTATATATTCA
Consensus

Transcriptome NGS   --------------------------------------------------   2300
Viral RNA NGS       TCCAAATTGTCTGAAAAATAATTAAGACATGACAAGATTGATCTCAGTTC
Sanger              TCCAAATTGTCTGAAAAATAATTAAGACATGACAAGATTGATCTCAGTTC
Consensus

Transcriptome NGS   --------------------------------------------------   2350
Viral RNA NGS       CTGGAAAGTAAGGTTAGGGAATATCCCTTGAAGCCTGTCTGTGACATACA
Sanger              CTGGAAAGTAAGGTTAGGGAATATCCCTTGAAGCCTGTCTGTGACATACA
Consensus

Transcriptome NGS   --------------------------------------------------   2400
Viral RNA NGS       TTCCGCAGTCGAGAGCTCTAGATAAAGAGACGTCTTCAGTCTGCTTTTTC
Sanger              TTCCGCAGTCGAGAGCTCTAGATAAAGAGACGTCTTCAGTCTGCTTTTTC
Consensus

Transcriptome NGS   --------------------------------------------------   2450
Viral RNA NGS       AAGGTCCTTGTACTTACGGAGATGCTCCAATCAATAATAGCTATGAAGAA
Sanger              AAGGTCCTTGTACTTACGGAGATGCTCCAATCAATAATAGCTATGAAGAA
Consensus

Transcriptome NGS   --------------------------------------------------   2500
Viral RNA NGS       GACAAACGCTTCTCTGCTATACACTGTGCTCAGAAAACTGGTATAATTTG
Sanger              GACAAACGCTTCTCTGCTATACACTGTGCTCAGAAAACTGGTATAATTTG
Consensus

Transcriptome NGS   --------------------------------------------------   2550
Viral RNA NGS       CTGTGCTTGTTCTACCCATGTTGTAAAGAGAGTAAACTGGGAAAGCTTTT
Sanger              CTGTGCTTGTTCTACCCATGTTGTAAAGAGAGTAAACTGGGAAAGCTTTT
Consensus

Transcriptome NGS   --------------------------------------------------   2600
Viral RNA NGS       CTGTTGCAGCATGTATTCAATGTCATCAGCTTGTTCTGACAACTAATCAT
Sanger              CTGTTGCAGCATGTATTCAATGTCATCAGCTTGTTCTGACAACTAATCAT
Consensus

Transcriptome NGS   --------------------------------------------------   2650
Viral RNA NGS       GTGATTGCTAGCTAAGACCCTTCTCTTCAGATAATTAGAAGCTCTAAACT
Sanger              GTGATTGCTAGCTAAGACCCTTCTCTTCAGATAATTAGAAGCTCTAAACT
Consensus

Transcriptome NGS   --------------------------------------------------   2700
Viral RNA NGS       CATCAGATGAATAGTTATCAGCATCTGACAGCACACATTTTATGTCCATT
Sanger              CATCAGATGAATAGTTATCAGCATCTGACAGCACACATTTTATGTCCATT
Consensus

Transcriptome NGS   --------------------------------------------------   2750
Viral RNA NGS       ATCTGCGAAGAAGATCCGAACATAGCTGCCACCATAGAAACAGCATTTTC
Sanger              ATCTGCGAAGAAGATCCGAACATAGCTGCCACCATAGAAACAGCATTTTC
Consensus

Transcriptome NGS   --------------------------------------------------   2800
Viral RNA NGS       TGTCAAAGGGATATTGGTTAGACCAAGTTTCTCAAGATATTGGACCCCGT
Sanger              TGTCAAAGGGATATTGGTTAGACCAAGTTTCTCAAGATATTGGACCCCGT
Consensus
```

```
Transcriptome NGS   --------------------------------------------------  2850
Viral RNA NGS       AAAACTCTTTCATAACTATTAATCCAGGTGAGCCTTCAAAGATCCTTGTA
Sanger              AAAACTCTTTCATAACTATTAATCCAGGTGAGCCTTCAAAGATCCTTGTA
Consensus

Transcriptome NGS   --------------------------------------------------  2900
Viral RNA NGS       AACCTTTCTGGTATTATCCATCTAAAGTTGTAATTTTGTTTAGGGTTCGA
Sanger              AACCTTTCTGGTATTATCCATCTAAAGTTGTAATTTTGTTTAGGGTTCGA
Consensus

Transcriptome NGS   --------------------------------------------------  2950
Viral RNA NGS       GTTTGTCACAATCATTTTATCTGTGATCTCAACTATCATTTGGAAATTGA
Sanger              GTTTGTCACAATCATTTTATCTGTGATCTCAACTATCATTTGGAAATTGA
Consensus

Transcriptome NGS   --------------------------------------------------  3000
Viral RNA NGS       CATCATTTGATATCAAAGAGACTAAGATATTCAGGATAGCTGGCAAATTC
Sanger              CATCATTTGATATCAAAGAGACTAAGATATTCAGGATAGCTGGCAAATTC
Consensus

Transcriptome NGS   ---------------------TCTAAGTGGGACACAATCAACTCTCGATA  3050
Viral RNA NGS       TCTCTTTCTTTGAAGCATTTCTCTAAGTGGGACACAATCAACTCTCGATA
Sanger              TCTCTTTCTTTGAAGCATTTCTCTAAGTGGGACACAATCAACTCTCGATA
Consensus                                *****************************

Transcriptome NGS   TGATACTTTCTTGTCAAAGTCATCGATAGAAAATGTTTTTGCTTTTGATC  3100
Viral RNA NGS       TGATACTTTCTTGTCAAAGTCATCGATAGAAAATGTTTTTGCTTTTGATC
Sanger              TGATACTTTCTTGTCAAAGTCATCGATAGAAAATGTTTTTGCTTTTGATC
Consensus           **************************************************

Transcriptome NGS   TAAGAATTCTGTCTATAGCCAACTTTTCTGTAGATTGGTTGCATATTTGA  3150
Viral RNA NGS       TAAGAATTCTGTCTATAGCCAACTTTTCTGTAGATTGGTTGCATATTTGA
Sanger              TAAGAATTCTGTCTATAGCCAACTTTTCTGTAGATTGGTTGCATATTTGA
Consensus           **************************************************

Transcriptome NGS   TTTCTTTTAGCAACTGAGGAATACTGCATGATTCTGGATCTCAAATAATC  3200
Viral RNA NGS       TTTCTTTTAGCAACTGAGGAATACTGCATGATTCTGGATCTCAAATAATC
Sanger              TTTCTTTTAGCAACTGAGGAATACTGCATGATTCTGGATCTCAAATAATC
Consensus           **************************************************

Transcriptome NGS   TCTATCATTTAGAGGTGCTATCAAGGTCAATTCTGGATGGCTTAATATCT  3250
Viral RNA NGS       TCTATCATTTAGAGGTGCTATCAAGGTCAATTCTGGATGGCTTAATATCT
Sanger              TCTATCATTTAGAGGTGCTATCAAGGTCAATTCTGGATGGCTTAATATCT
Consensus           **************************************************

Transcriptome NGS   CATCATAGAGTATCATCATTTCTTCTCTAGATCTATAATCCTTTGAATAA  3300
Viral RNA NGS       CATCATAGAGTATCATCATTTCTTCTCTAGATCTATAATCCTTTGAATAA
Sanger              CATCATAGAGTATCATCATTTCTTCTCTAGATCTATAATCCTTTGAATAA
Consensus           **************************************************

Transcriptome NGS   TCATCAAAATAATCTTCTTTTACATTCTTTAAAGCAGCAACCAAATCGTC  3350
Viral RNA NGS       TCATCAAAATAATCTTCTTTTACATTCTTTAAAGCAGCAACCAAATCGTC
Sanger              TCATCAAAATAATCTTCTTTTACATTCTTTAAAGCAGCAACCAAATCGTC
Consensus           **************************************************

Transcriptome NGS   ATTCTCTTTCAAGTTTGGAAACTCTTTAACAAAATCTCTAAAGCTTGGCA  3400
Viral RNA NGS       ATTCTCTTTCAAGTTTGGAAACTCTTTAACAAAATCTCTAAAGCTTGGCA
Sanger              ATTCTCTTTCAAGTTTGGAAACTCTTTAACAAAATCTCTAAAGCTTGGCA
Consensus           **************************************************

Transcriptome NGS   TGGATGCCATCAGGCTTTTGTTTATATAGTTAGGTAAGTTTATTATCTGG  3450
Viral RNA NGS       TGGATGCCATCAGGCTTTTGTTTATATAGTTAGGTAAGTTTATTATCTGG
Sanger              TGGATGCCATCAGGCTTTTGTTTATATAGTTAGGTAAGTTTATTATCTGG
Consensus           **************************************************

Transcriptome NGS   CTAGAGAAAGATTGAAACTTGGTACCATATTCCAATGTTTCTGAGTTATC  3500
Viral RNA NGS       CTAGAGAAAGATTGAAACTTGGTACCATATTCCAATGTTTCTGAGTTATC
Sanger              CTAGAGAAAGATTGAAACTTGGTACCATATTCCAATGTTTCTGAGTTATC
Consensus           **************************************************
```

```
Transcriptome NGS   TGCAGCTGTACTCAATCGAAATAAATTAATTATAGCAATACATGCTTTCA  3550
Viral RNA NGS       TGCAGCTGTACTCAATCGAAATAAATTAATTATAGCAATACATGCTTTCA
Sanger              TGCAGCTGTACTCAATCGAAATAAATTAATTATAGCAATACATGCTTTCA
Consensus           **************************************************

Transcriptome NGS   TCTCAGGATCATTACTGACCTCTAATTCTCTAGCAATGCTTGCACTCTTT  3600
Viral RNA NGS       TCTCAGGATCATTACTGACCTCTAATTCTCTAGCAATGCTTGCACTCTTT
Sanger              TCTCAGGATCATTACTGACCTCTAATTCTCTAGCAATGCTTGCACTCTTT
Consensus           **************************************************

Transcriptome NGS   GATACTGTAGAATTTCTAACTTTGTCTTGGAATTCTGAGAAATTCTTCGT  3650
Viral RNA NGS       GATACTGTAGAATTTCTAACTTTGTCTTGGAATTCTGAGAAATTCTTCGT
Sanger              GATACTGTAGAATTTCTAACTTTGTCTTGGAATTCTGAGAAATTCTTCGT
Consensus           **************************************************

Transcriptome NGS   CCTGAGTACTTTAGAGCAACTCTGGTAATATATGAGCTCATCATTACAAG  3700
Viral RNA NGS       CCTGAGTACTTTAGAGCAACTCTGGTAATATATGAGCTCATCATTACAAG
Sanger              CCTGAGTACTTTAGAGCAACTCTGGTAATATATGAGCTCATCATTACAAG
Consensus           **************************************************

Transcriptome NGS   ATGGTCCTAGCACTGAAAGCATGTCAACGGGGGCAACCATCCATCCACCT  3750
Viral RNA NGS       ATGGTCCTAGCACTGAAAGCATGTCAACGGGGGCAACCATCCATCCACCT
Sanger              ATGGTCCTAGCACTGAAAGCATGTCAACGGGGGCAACCATCCATCCACCT
Consensus           **************************************************

Transcriptome NGS   ATGCAAGCCGGAACATTGTAAGATCCTGGAGAGATCTTTAGATTCATGTT  3800
Viral RNA NGS       ATGCAAGCCGGAACATTGTAAGATCCTGGAGAGATCTTTAGATTCATGTT
Sanger              ATGCAAGCCGGAACATTGTAAGATCCTGGAGAGATCTTTAGATTCATGTT
Consensus           **************************************************

Transcriptome NGS   AGTTTCTTTTAGAATTGCATGTATGTCATTGGTTTCTCCTGGCAGCATAG  3850
Viral RNA NGS       AGTTTCTTTTAGAATTGCATGTATGTCATTGGTTTCTCCTGGCAGCATAG
Sanger              AGTTTCTTTTAGAATTGCATGTATGTCATTGGTTTCTCCTGGCAGCATAG
Consensus           **************************************************

Transcriptome NGS   AATACAAAGATAGTGATTGTATTTGCACGGCAGCATATGCAAATGCTATG  3900
Viral RNA NGS       AATACAAAGATAGTGATTGTATTTGCACGGCAGCATATGCAAATGCTATG
Sanger              AATACAAAGATAGTGATTGTATTTGCACGGCAGCATATGCAAATGCTATG
Consensus           **************************************************

Transcriptome NGS   ATTTCATTGGGACAACCTTTCCTAGCTAACATGGTGATATTGATACTCAG  3950
Viral RNA NGS       ATTTCATTGGGACAACCTTTCCTAGCTAACATGGTGATATTGATACTCAG
Sanger              ATTTCATTGGGACAACCTTTCCTAGCTAACATGGTGATATTGATACTCAG
Consensus           **************************************************

Transcriptome NGS   AGACATCAGATCATCATAATAGTTTTGGTGTGAAGCTTCTGTGCAGCAGT  4000
Viral RNA NGS       AGACATCAGATCATCATAATAGTTTTGGTGTGAAGCTTCTGTGCAGCAGT
Sanger              AGACATCAGATCATCATAATAGTTTTGGTGTGAAGCTTCTGTGCAGCAGT
Consensus           **************************************************

Transcriptome NGS   TTGCAAGGTTCCTGCAGTATGGTGGTATAACTGCACCGTTTATGAATTTT  4050
Viral RNA NGS       TTGCAAGGTTCCTGCAGTATGGTGGTATAACTGCACCGTTTATGAATTTT
Sanger              TTGCAAGGTTCCTGCAGTATGGTGGTATAACTGCACCGTTTATGAATTTT
Consensus           **************************************************

Transcriptome NGS   TCTGAAATGAATTCTACCTGAGATTCAGCAGCATATGTTTTCTTCGGATT  4100
Viral RNA NGS       TCTGAAATGAATTCTACCTGAGATTCAGCAGCATATGTTTTCTTCGGATT
Sanger              TCTGAAATGAATTCTACCTGAGATTCAGCAGCATATGTTTTCTTCGGATT
Consensus           **************************************************

Transcriptome NGS   CAATGTTATGGAGAAGCTAGGGAAATGATGTTTCATGCTCAAAAAATGAA  4150
Viral RNA NGS       CAATGTTATGGAGAAGCTAGGGAAATGATGTTTCATGCTCAAAAAATGAA
Sanger              CAATGTTATGGAGAAGCTAGGGAAATGATGTTTCATGCTCAAAAAATGAA
Consensus           **************************************************

Transcriptome NGS   ATTCGCATAAAGATTCACATTTGAATTTTGAAAGTAGCCTTTTCACATCT  4200
Viral RNA NGS       ATTCGCATAAAGATTCACATTTGAATTTTGAAAGTAGCCTTTTCACATCT
Sanger              ATTCGCATAAAGATTCACATTTGAATTTTGAAAGTAGCCTTTTCACATCT
Consensus           **************************************************
```

```
Transcriptome NGS   CCTGAAGCAATCATGGATGTTGCATTATCATCTGAGTGTACCATCCATTT  4250
Viral RNA NGS       CCTGAAGCAATCATGGATGTTGCATTATCATCTGAGTGTACCATCCATTT
Sanger              CCTGAAGCAATCATGGATGTTGCATTATCATCTGAGTGTACCATCCATTT
Consensus           **************************************************

Transcriptome NGS   TATTTTAAATTCAACACCTTCCATCTGCTTCAATATGTTTTTGTAGCAAA  4300
Viral RNA NGS       TATTTTAAATTCAACACCTTCCATCTGCTTCAATATGTTTTTGTAGCAAA
Sanger              TATTTTAAATTCAACACCTTCCATCTGCTTCAATATGTTTTTGTAGCAAA
Consensus           **************************************************

Transcriptome NGS   GCATGGCACAAGAATGATAGACAGATGACAAATAGTTTATGTTTCCTTGC  4350
Viral RNA NGS       GCATGGCACAAGAATGATAGACAGATGACAAATAGTTTATGTTTCCTTGC
Sanger              GCATGGCACAAGAATGATAGACAGATGACAAATAGTTTATGTTTCCTTGC
Consensus           **************************************************

Transcriptome NGS   AACCAGTTCATGCTTACTGGATACCAGTTTCTACTTAAACCTTTAGTGGC  4400
Viral RNA NGS       AACCAGTTCATGCTTACTGGATACCAGTTTCTACTTAAACCTTTAGTGGC
Sanger              AACCAGTTCATGCTTACTGGATACCAGTTTCTACTTAAACCTTTAGTGGC
Consensus           **************************************************

Transcriptome NGS   CTCAAAAAGTGAATTCATGACATCACCATATTCTTCTTTACCCATGACCA  4450
Viral RNA NGS       CTCAAAAAGTGAATTCATGACATCACCATATTCTTCTTTACCCATGACCA
Sanger              CTCAAAAAGTGAATTCGTGACATCACCATATTCTTCTTTACCCATGACCA
Consensus           **************** *********************************

Transcriptome NGS   TGCCTAAGAAGATGTCTGTCGGTATCACAACTCGCTTGTGCTTGATGTAC  4500
Viral RNA NGS       TGCCTAAGAAGATGTCTGTCGGTATCACAACTCGCTTGTGCTTGATGTAC
Sanger              TGCCTAAGAAGATGTCTGTCGGTATCACAACTCGCTTGTGCTTGATGTAC
Consensus           **************************************************

Transcriptome NGS   ATCAGGAAGCACTCAATCATTAGTGTTCTTTCACCAGGTGTGAATATGGG  4550
Viral RNA NGS       ATCAGGAAGCACTCAATCATTAGTGTTCTTTCACCAGGTGTGAATATGGG
Sanger              ATCAGGAAGCACTCAATCATTAGTGTTCTTTCACCAGGTGTGAATATGGG
Consensus           **************************************************

Transcriptome NGS   ACACATCAATATAGCTAGGATATATTTATACGACATATCAGAAGCAGACC  4600
Viral RNA NGS       ACACATCAATATAGCTAGGATATATTTATACGACATATCAGAAGCAGACC
Sanger              ACACATCAATATAGCTAGGATATATTTATACGACATATCAGAAGCAGACC
Consensus           **************************************************

Transcriptome NGS   ACTTGGATTGATCAGCTGAAACAAAAGACAATTTAGCGCCTTGATCTCTT  4650
Viral RNA NGS       ACTTGGATTGATCAGCTGAAACAAAAGACAATTTAGCGCCTTGATCTCTT
Sanger              ACTTGGATTGATCAGCTGAAACAAAAGACAATTTAGCGCCTTGATCTCTT
Consensus           **************************************************

Transcriptome NGS   GACAGGATCTCATTGTAAGATGAAATTGCTGTTAAAGATAAATTAGACAA  4700
Viral RNA NGS       GACAGGATCTCATTGTAAGATGAAATTGCTGTTAAAGATAAATTAGACAA
Sanger              GACAGGATCTCATTGTAAGATGAAATTGCTGTTAAAGATAAATTAGACAA
Consensus           **************************************************

Transcriptome NGS   TGTTTTTATTTTGTAATCACCAGATATAGATATGGCTTCACTGGGATCAG  4750
Viral RNA NGS       TGTTTTTATTTTGTAATCACCAGATATAGATATGGCTTCACTGGGATCAG
Sanger              TGTTTTTATTTTGTAATCACCAGATATAGATATGGCTTCACTGGGATCAG
Consensus           **************************************************

Transcriptome NGS   ATTGAGCTACATGCTTAAAAGAATGCTCAATAAAATATAACATCAATTTG  4800
Viral RNA NGS       ATTGAGCTACATGCTTAAAAGAATGCTCAATAAAATATAACATCAATTTG
Sanger              ATTGAGCCACATGCTTAAAAGAATGCTCAATAAAATATAACATCAATTTG
Consensus           ******* ******************************************

Transcriptome NGS   CAGCGAAAGCCCATTAGATAGATTTCTCTATCTGTTTTAGTCCTCTGATG  4850
Viral RNA NGS       CAGCGAAAGCCCATTAGATAGATTTCTCTATCTGTTTTAGTCCTCTGATG
Sanger              CAGCGAAAGCCCATTAGATAGATTTCTCTATCTGTTTTAGTCCTCTGATG
Consensus           **************************************************

Transcriptome NGS   CTTTTCAAACACGGATACAAGATACAATTGATATTTCTCAAGTTGATCAA  4900
Viral RNA NGS       CTTTTCAAACACGGATACAAGATACAATTGATATTTCTCAAGTTGATCAA
Sanger              CTTTTCAAACACGGATACAAGATACAATTGATATTTCTCAAGTTGATCAA
Consensus           **************************************************
```

```
Transcriptome NGS    TGCTCGATATAACGTATTCAAGCAGCTCCATAAAAGTGTGTTTCTTCCCA  4950
Viral RNA NGS        TGCTCGATATAACGTATTCAAGCAGCTCCATAAAAGTGTGTTTCTTCCCA
Sanger               TGCTCGATATAACGTATTCAAGCAGCTCCATAAAAGTGTGTTTCTTCCCA
Consensus            **************************************************

Transcriptome NGS    GAAATGCCATTATTGGGGTCTATCTGCATCAAGCTATTGAAAGAAGTACT  5000
Viral RNA NGS        GAAATGCCATTATTGGGGTCTATCTGCATCAAGCTATTGAAAGAAGTACT
Sanger               GAAATGCCATTATTGGGGTCTATCTGCATCAAGCTATTGAAAGAAGTACT
Consensus            **************************************************

Transcriptome NGS    TACAATGCCATGATATTCTTTTATAATTTGATAAAGACACTCAGACACTT  5050
Viral RNA NGS        TACAATGCCATGATATTCTTTTATAATTTGATAAAGACACTCAGACACTT
Sanger               TACAATGCCATGATATTCTTTTATAATTTGATAAAGACACTCAGACACTT
Consensus            **************************************************

Transcriptome NGS    CTTCTGATTTTAGCGTTTGTGATTCCTTAGTGTTAGTTATCAAGCACAAG  5100
Viral RNA NGS        CTTCTGATTTTAGCGTTTGTGATTCCTTAGTGTTAGTTATCAAGCACAAG
Sanger               TTTCTGATTTTAGCGTTTGTGATTCCTTAGTGTTAGTTATCAAGCACAAG
Consensus             *************************************************

Transcriptome NGS    AACACTTCCACTGATATAGGGTGTTGGGTCCTAGTCATCTTTCTTGGGTT  5150
Viral RNA NGS        AACACTTCCACTGATATAGGGTGTTGGGTCCTAGTCATCTTTCTTGGGTT
Sanger               AACACTTCCACTGATATAGGGTGTTGGGTCCTAGTCATCTTTCTTGGGTT
Consensus            **************************************************

Transcriptome NGS    AAAGTTCCAGAGATGAGAAAATCTTTTGGTATTCAACTCTTCGAGAATTT  5200
Viral RNA NGS        AAAGTTCCAGAGATGAGAAAATCTTTTGGTATTCAACTCTTCGAGAATTT
Sanger               AAAGTTCCAGAGATGAGAAAATCTTTTGGTATTCAACTCTTCGAGAATTT
Consensus            **************************************************

Transcriptome NGS    TTATGATCTCGTCATCGAAGCCCTCAACAAAAGACTTGCCAAACCTATAG  5250
Viral RNA NGS        TTATGATCTCGTCATCGAAGCCCTCAACAAAAGACTTGCCAAACCTATAG
Sanger               TTATGATCTCGTCATCGAAGCCCTCAACAAAAGACTTGCCAAACCTATAG
Consensus            **************************************************

Transcriptome NGS    CTGAACATTACCCCTTTAATATAATGAAAATTTACTAATGATGTCATTTT  5300
Viral RNA NGS        CTGAACATTACCCCTTTAATATAATGAAAATTTACTAATGATGTCATTTT
Sanger               CTGAACATTACCCCTTTAATATAATGAAAATTTACTAATGATGTCATTTT
Consensus            **************************************************

Transcriptome NGS    GTTAATTAATGTTTCAAAATTTGACACCTCGGCTTTCAATGCTTTAAAAT  5350
Viral RNA NGS        GTTAATTAATGTTTCAAAATTTGACACCTCGGCTTTCAATGCTTTAAAAT
Sanger               GTTAATTAATGTTTCAAAATTTGACACCTCGGCTTTCAATGCTTTAAAAT
Consensus            **************************************************

Transcriptome NGS    CAGAGATGTAGATCCTCACATGCTTTTTTATGTCGTCTAGTTTCATTAAC  5400
Viral RNA NGS        CAGAGATGTAGATCCTCACATGCTTTTTTATGTCGTCTAGTTTCATTAAC
Sanger               CAGAGATGTAGATCCTCACATGCTTTTTTATGTCGTCTAATTTCATTAAC
Consensus            *************************************** **********

Transcriptome NGS    TTAGGATTAGAGCACTTTTTAGAAGAGTTTAAAGTGTTTGTTTTGTAACA  5450
Viral RNA NGS        TTAGGATTAGAGCACTTTTTAGAAGAGTTTAAAGTGTTTGTTTTGTAACA
Sanger               TTAGGATTAGAGCACTTTTTAGAAGAGTTTAAAGTGTTTGTTTTGTAACA
Consensus            **************************************************

Transcriptome NGS    GGGTTGGAGAAAATTCTCTTTAGTTTCAATATTGTTTCTAACTACATTTA  5500
Viral RNA NGS        GGGTTGGAGAAAATTCTCTTTAGTTTCAATATTGTTTCTAACTACATTTA
Sanger               GGGTTGGAGAAAATTCTCTTTAGTTTCAATATTGTTTCTAACTACATTTA
Consensus            **************************************************

Transcriptome NGS    CATTGGGCACATGCAATTTGTAATATTCTTTCAAAGATAGAACTGAGGCT  5550
Viral RNA NGS        CATTGGGCACATGCAATTTGTAATATTCTTTCAAAGATAGAACTGAGGCT
Sanger               CATTGGGCACATGCAATTTGTAATATTCTTTCAAAGATAGAACTGAGGCT
Consensus            **************************************************

Transcriptome NGS    ACTCCATCTATAGAAAAAGGTCCTTTGCCTTTAAACATTGCTTCTTTTGG  5600
Viral RNA NGS        ACTCCATCTATAGAAAAAGGTCCTTTGCCTTTAAACATTGCTTCTTTTGG
Sanger               ACTCCATCTATAGAAAAAGGTCCTTTGCCTTTAAACATTGCTTCTTTTGG
Consensus            **************************************************
```

```
Transcriptome NGS   CTTAATCTCATCATCCAGGCTAAAATTAAAATGCTTCCTCATTTTTAATT  5650
Viral RNA NGS       CTTAATCTCATCATCCAGGCTAAAATTAAAATGCTTCCTCATTTTTAATT
Sanger              CTTAATCTCATCATCCAGGCTAAAATTAAAATGCTTCCTCATTTTTAATT
Consensus           **************************************************

Transcriptome NGS   CCCATTCAGCTGAAACTGACAGTAATGATTTCATATTATGTTCATGATTA  5700
Viral RNA NGS       CCCATTCAGCTGAAACTGACAGTAATGATTTCATATTATGTTCATGATTA
Sanger              CCCATTCAGCTGAAACTGACAGTAATGATTTCATATTATGTTCATGATTA
Consensus           **************************************************

Transcriptome NGS   TGCATACTTTTTGGTAGCATGTAGATCCCCAAACTTATATTGTTATGCAA  5750
Viral RNA NGS       TGCATACTTTTTGGTAGCATGTAGATCCCCAAACTTATATTGTTATGCAA
Sanger              TGCATACTTTTTGGTAGCATGTAGATCCCCAAACTTATATTGTTATGCAA
Consensus           **************************************************

Transcriptome NGS   GTCTTCATATTTACTCAAAGGTATATTTGTGACAGGACAAATAACGTCCA  5800
Viral RNA NGS       GTCTTCATATTTACTCAAAGGTATATTTGTGACAGGACAAATAACGTCCA
Sanger              GTCTTCATATTTACTCAAAGGTATATTTGTGACAGGACAAATAACGTCCA
Consensus           **************************************************

Transcriptome NGS   AATCTTTTATTCCTCCGGATATATCCATTTCTTGATCTATAACAATAGTT  5850
Viral RNA NGS       AGTCTTTTATTCCTCCGGATATATCCATTTCTTGATCTATAACAATAGTT
Sanger              AGTCTTTTATTCCTCCGGATATATCCATTTCTTGATCTATAACAATGGTT
Consensus           * ******************************************** ***

Transcriptome NGS   CTAGCCTTTTCAGAAAGGCTCATTCCCATCATCTTATTCAAAAACCTTTT  5900
Viral RNA NGS       CTAGCCTTTTCAGAAAGGCTCATTCCCATCATCTTATTCAAAAACCTTTT
Sanger              CTAGCCTTTTCAGAAAGGCTCATTCCCATCATCTTATTCAAAAACCTTTT
Consensus           **************************************************

Transcriptome NGS   TATGCCTATCATAAGATGGAAATCTACACAATTTGTAATTTCTGGATCAA  5950
Viral RNA NGS       TATGCCTATCATAAGATGGAAATCTACACAATTTGTAATTTCTGGATCAA
Sanger              TATGCCTATCATAAGATGGAAATCTACACAATTTGTAATTTCTGGATCAA
Consensus           **************************************************

Transcriptome NGS   ATTTCTCAGAAATATACTCTTTGACATTTGAGTAGTCAGCTAATGGTAAA  6000
Viral RNA NGS       ATTTCTCAGAAATATACTCTTTGACATTTGAGTAATCAGCTAATGGTAAA
Sanger              ATTTCTCAGAAATATACTCTTTGACGTTTGAGTAATCAGCTAATGGTAAA
Consensus           ************************* ******** ***************

Transcriptome NGS   AATCCTGCATATCTCATGAAATCAAACATTATCATTCCTGATAGATTAGT  6050
Viral RNA NGS       AATCCTGCATATCTCATGAAATCAAACATTATCATTCCTGATAGATTAGT
Sanger              AATCCTGCATATCTCATGAAATCAAACATTATCATTCCTGATAGATTAGT
Consensus           **************************************************

Transcriptome NGS   T-------------------------------------------------  6100
Viral RNA NGS       TATAGAGCTTATGGCTTTACACGGAAACCACACATCTCTTATAATGGATT
Sanger              TATAGAGCTTATGGCTTTACACGGAAACCACACATCTCTTATAATGGATT
Consensus           *

Transcriptome NGS   --------------------------------------------------  6150
Viral RNA NGS       TTATTTCCTCGTCAAAAAGCACAGTCTTATCATAACCTTTTTTTACCATG
Sanger              TTATTTCCTCGTCAAAAAGCACAGTCTTATCATAACCTTTTTTTACCATG
Consensus

Transcriptome NGS   --------------------------------------------------  6200
Viral RNA NGS       TTTTTTATGATCTCAGACCGTGACAAGTACGTTGCAAAACAAGGAGGCAT
Sanger              TTTTTTATGATCTCAGACCGTGACAAGTTCGCTGCAAAACAAGGAGGCAT
Consensus

Transcriptome NGS   --------------------------------------------------  6250
Viral RNA NGS       TTTGCTAGGACTTTTAAAAAGGCTGAGCAATCTCACTTGGTTTAGCCTTT
Sanger              TTTGCTAGGACTTTTAAAAAGGCTGAGCAATCTCACTTGGTTTAGCCTTT
Consensus

Transcriptome NGS   --------------------------------------------------  6300
Viral RNA NGS       GAGGCCTCATCAAGAAGTATGTATTCCTTTTATAATTGAAAAATCCGTAT
Sanger              GAGGCCTCATCAAGAAGTATGTATTCCTTTTATAATTGAAAAATCCGTAT
Consensus
```

Transcriptome NGS	--	6350
Viral RNA NGS	ATTTCCTTTGTATATCCGATTCTGAAGTTTTCTGACAATGCACCTGTCTC	
Sanger	ATTTCCTTTGTATATCCGATTCTGAAGTTTTCTGACAATGCACCTGTCTC	
Consensus		
Transcriptome NGS	--	6400
Viral RNA NGS	TACTTTGTGAAGCATGACAAATGGCACACCAGCTGTACCTGTATTTATAC	
Sanger	TACTTTGTGAAGCATGACAAATGGCACACCAGCTGTACCTGTATTTATAC	
Consensus		
Transcriptome NGS	--	6450
Viral RNA NGS	CATCACCTTTGAAAGCTATGGCCATCAAAGAACTGTTGGAACAATTTAGA	
Sanger	CATCACCTTTGAAAGCTATGGCCATCAAAGAACTGTTGGAACAATTTAGA	
Consensus		
Transcriptome NGS	--	6500
Viral RNA NGS	AATTTGAAATCTGTGCCACTCATAAACCTATCTGCAGTCATCAAACCCTT	
Sanger	AATTTGAAATCTGTGCCACTCATAAACCTATCTGCAGTCATCAAACCCTT	
Consensus		
Transcriptome NGS	--	6550
Viral RNA NGS	TGATATCTGCATTTGATCCTCTAAGAAGGAATAGATTCCAATGTCTTTCA	
Sanger	TGATATCTGCATTTGATCCTCTAAGAAGGAATAGATTCCAATGTCTTTCA	
Consensus		
Transcriptome NGS	--	6600
Viral RNA NGS	TTATAGAGTTCATGGATCTACAAAGGTTCA**T**CTCATCAGGAATACAACCA	
Sanger	TTATAGAGTTCATGGATCTACAAAGGTTCA**C**CTCATCAGGAATACAACCA	
Consensus		
Transcriptome NGS	--	6650
Viral RNA NGS	GATTTCATGAAAAGGCATTCAGGATTATCTGCATAGAAGTCATATACAGT	
Sanger	GATTTCATGAAAAGGCATTCAGGATTATCTGCATAGAAGTCATATACAGT	
Consensus		
Transcriptome NGS	--	6700
Viral RNA NGS	TGAGATCTTCCCTTTCTCTCTTTTATCTTTTAATTGGAGCTCAAAGAGGT	
Sanger	TGAGATCTTCCCTTTCTCTCTTTTATCTTTTAATTGGAGCTCAAAGAGGT	
Consensus		
Transcriptome NGS	--	6750
Viral RNA NGS	TTGCTTTGATTTGATCTACCCAGGCTTCTACTGTTGTGTTATTGTGCCGT	
Sanger	TTGCTTTGATTTGATCTACCCAGGCTTCTACTGTTGTGTTATTGTGCCGT	
Consensus		
Transcriptome NGS	--	6800
Viral RNA NGS	ATCAGCTTTTTCCTGTGAACAGTTTTGTTGATGTCTTCTTGATTGAAGTA	
Sanger	ATCAGCTTTTTCCTGTGAACAGTTTTGTTGATGTCTTCTTGATTGAAGTA	
Consensus		
Transcriptome NGS	--	6850
Viral RNA NGS	ATGTTTGCTTATGTCCTGCTCTCCATCACTTTCTTCACTGCTTGTTTGAT	
Sanger	ATGTTTGCTTATGTCCTGCTCTCCATCACTTTCTTCACTGCTTGTTTGAT	
Consensus		
Transcriptome NGS	--	6900
Viral RNA NGS	CGTCTTGCTTTTTAGAGTTCTCTTCTTCTCTCTCTTTTATCTTCATTATT	
Sanger	CGTCTTGCTTTTTAGAGTTCTCTTCTTCTCTCTCTTTTATCTTCATTATT	
Consensus		
Transcriptome NGS	--	6950
Viral RNA NGS	TTATCTTTCAACCTAGCTCTGTTTCTATTCTTGGCAGTCTTGGACATATT	
Sanger	TTATCTTTCAACCTAGCTCTGTTTCTATTCTTGGCAGTCTTGGACATATT	
Consensus		
Transcriptome NGS	--	7000
Viral RNA NGS	TACTTTCATTTCCCTCAGGCCTTCATAAAAAAACCTCCTCTCTTCTTCTT	
Sanger	TACTTTCATTTCCCTCAGGCCTTCATAAAAAAACCTCCTCTCTTCTTCTT	
Consensus		

```
Transcriptome NGS   --------------------------------------------------   7050
Viral RNA NGS       TACCTTTGATGTTGACATTAAATTCATTCTTGTTTCTAGTCAAAAATCCA
Sanger              TACCTTTGATGTTGACATTAAATTCATTCTTGTTTCTAGTCAAAAATCCA
Consensus

Transcriptome NGS   --------------------------------------------------   7100
Viral RNA NGS       TTATAATCATGCCACTTTTTACCCTCACTAGTCCTTTCTAAATCTATCCC
Sanger              TTATAATCATGCCACTTTTTACCCTCACTAGTCCTTTCTAAATCTATCCC
Consensus

Transcriptome NGS   --------------------------------------------------   7150
Viral RNA NGS       TCCAGTATTCAGATTAGCCACGTCCTCCATTTCTGATTTATCACACTTTG
Sanger              TCCAGTATTCAGATTAGCCACGTCCTCCATTTCTGATTTATCACACTTTG
Consensus

Transcriptome NGS   --------------------------------------------------   7200
Viral RNA NGS       TTGTATTAGTAGAAAAGTCGTTCAAACAGCATCTTACTAGATTGTAAATC
Sanger              TTGTATTAGTAGAAAAGTCGTTCAAACAGCATCTTACTAGATTGTAAATC
Consensus

Transcriptome NGS   --------------------------------------------------   7250
Viral RNA NGS       CTCATGACTTTGGTTCTATTTAAAGCTGCAGAGTGGTATGGGTTATTTAT
Sanger              CTCATGACTTTGGTTCTATTTAAAGCTGCAGAGTGGTATGGGTTATTTAT
Consensus

Transcriptome NGS   --------------------------------------------------   7300
Viral RNA NGS       GACTATGCTGGGAGTCCTGTCAAATGCAACTCTAAAATCATTAATCACAT
Sanger              GACTATGCTGGGAGTCCTGTCAAATGCAACTCTAAAATCATTAATCACAT
Consensus

Transcriptome NGS   --------------------------------------------------   7350
Viral RNA NGS       AGTTGTTGTAATCCATCTCATCGGAGACAACGACATCTTCTCTGTGAGGA
Sanger              AGTTGTTGTAATCCATCTCATCGGAGACAACGACATCTTCTCTGTGAGGA
Consensus

Transcriptome NGS   --------------------------------------------------   7400
Viral RNA NGS       GACCAAGAAAAAGCAAAGTTACATTTAGTATTAGCCAAGTCTTCTTGGAA
Sanger              GACCAAGAAAAAGCAAAGTTACATTTAGTATTAGCCAAGTCTTCTTGGAA
Consensus

Transcriptome NGS   --------------------------------------------------   7450
Viral RNA NGS       ACAACCTGGATTCTTGTTTTCATAATCTTTTGCATTCATTTCAAAAGCTT
Sanger              ACAACCTGGATTCTTGTTTTCATAATCTTTTGCATTCATTTCAAAAGCTT
Consensus

Transcriptome NGS   --------------------------------------------------   7500
Viral RNA NGS       CTTCTATATCTTTGATGTCTTTCCGAGTGTTGATTTCACGAACTACATTT
Sanger              CTTCTATATCTTTGATGTCTTTCCGAGTGTTGATTTCACGAACTACATTT
Consensus

Transcriptome NGS   --------------------------------------------------   7550
Viral RNA NGS       TCTTGCAATGTATCTAACATGTTCTTATATTTTAGGTCCTTTTTTAGGTA
Sanger              TCTTGCAATGTATCTAACATGTTCTTATATTTTAGGTCCTTTTTTAGGTA
Consensus

Transcriptome NGS   --------------------------------------------------   7600
Viral RNA NGS       AAGTTTGTCAATGTATTCTTTTAGCTTGATCACATCTGATTTAGGTTTGT
Sanger              AAGTTTGTCAATGTATTCTTTTAGCTTGATCACATCTGATTTAGGTTTGT
Consensus

Transcriptome NGS   --------------------------------------------------   7650
Viral RNA NGS       AACCCAATGATTCCATGTTATGTTTGTAAGATTCAAAAAGACTAGATATT
Sanger              AACCCAATGATTCCATGTTATGTTTGTAAGATTCAAAAAGACTAGATATT
Consensus

Transcriptome NGS   --------------------------------------------------   7700
Viral RNA NGS       TGATTCACAGTCATAGTTGACAAATGGTCTGTAAAATTCCCAAACATTAG
Sanger              TGATTCACAGTCATAGTTGACAAATGGTCTGTAAAATTCCCAAACATTAG
Consensus
```

```
Transcriptome NGS   --------------------------------------------------   7750
Viral RNA NGS       ATGGCAACATTCTTTGTTGGTGTACACATCATCACTAATGCTCAAAAGGA
Sanger              ATGGCAACATTCTTTGTTGGTGTACACATCATCACTAATGCTCAAAAGGA
Consensus

Transcriptome NGS   --------------------------------------------------   7800
Viral RNA NGS       TTGATCTCATATCTTCTGATTCTGTTAGAGTGTCAAAATCTTTACTTTTC
Sanger              TTGATCTCATATCTTCTGATTCTGTTAGAGTGTCAAAATCTTTACTTTTC
Consensus

Transcriptome NGS   --------------------------------------------------   7850
Viral RNA NGS       CACTGTTTAACCCACTCCTGAAAGTAATTCTTATCAGATTGTTCAATTAG
Sanger              CACTGTTTAACCCACTCCTGAAAGTAATTCTTATCAGATTGTTCAATTAG
Consensus

Transcriptome NGS   --------------------------------------------------   7900
Viral RNA NGS       CTCAGGGAAATTGTAAAGCATAAAATCCTTAAGCATCTGTGTACTAGCAT
Sanger              CTCAGGGAAATTGTAAAGCATAAAATCCTTAAGCATCTGTGTACTAGCAT
Consensus

Transcriptome NGS   --------------------------------------------------   7950
Viral RNA NGS       AAAAATCTATCAAGCCTTTGATATCTAATTGTGCAATCCTTTTTATGCTT
Sanger              AAAAATCTATCAAGCCTTTGATATCTAATTGTGCAATCCTTTTTATGCTT
Consensus

Transcriptome NGS   --------------------------------------------------   8000
Viral RNA NGS       AGAACATTGGTATTTTCTGCTGATTTCAACCTTGCATGAGTGGTTTTGTA
Sanger              AGAACATTGGTATTTTCTGCTGATTTCAACCTTGCATGAGTGGTTTTGTA
Consensus

Transcriptome NGS   --------------------------------------------------   8050
Viral RNA NGS       CAGACTTGTTTCAAGAGGGCCTTGAACTCTGCATTTTGTGCAAACTATTC
Sanger              CAGACTTGTTTCAAGAGGGCCTTGAACTCTGCATTTTGTGCAAACTATTC
Consensus

Transcriptome NGS   --------------------------------------------------   8100
Viral RNA NGS       TGTAATTTTCACTAGGAAGCAGAATGCAAAGGACCACATAGATAGGATTA
Sanger              TGTAATTTTCACTAGGAAGCAGAATGCAAAGGACCACATAGATAGGATTA
Consensus

Transcriptome NGS   --------------------------------------------------   8150
Viral RNA NGS       TCTTTAAGAAAGTTTCCACCATTGACTTTCTCTAGATACATGTATATACT
Sanger              TCTTTAAGAAAGTTTCCACCATTGACTTTCTCTAGATACATGTATATACT
Consensus

Transcriptome NGS   --------------------------------------------------   8200
Viral RNA NGS       ATTGTAGTACTTTTCTGCTGTGTGTATCTCTGAAGTGAAACTCACACTGA
Sanger              ATTGTAGTACTTTTCTGCTGTGTGTATCTCTGAAGTGAAACTCACACTGA
Consensus

Transcriptome NGS   --------------------------------------------------   8250
Viral RNA NGS       CTTTCCAATCATAGAAGATCAAAGATTTGCGTCCATCTTTTTGGTCTGAA
Sanger              CTTTCCAATCATAGAAGATCAAAGATTTGCGTCCATCTTTTTGGTCTGAA
Consensus

Transcriptome NGS   --------------------------------------------------   8300
Viral RNA NGS       TACAAGACATAATTGTCTGGTGTCAAGCCGGGCATAGTTCTGATTGCAGT
Sanger              TACAAGACATAATTGTCTGGTGTCAAGCCGGGCATAGTTCTGATTGCAGT
Consensus

Transcriptome NGS   --------------------------------------------------   8350
Viral RNA NGS       GTTTAATTCATCTTTATATTTATCAAAGCCTGGTGTTGATTCAAGTGATT
Sanger              GTTTAATTCATCTTTATATTTATCAAAGCCTGGTGTTGATTCAAGTGATT
Consensus

Transcriptome NGS   --------------------------------------------------   8400
Viral RNA NGS       TCATCAAGTCAAGCAAACCGTCTTGTATGGTACAGAAGTCTCTCTGTTTT
Sanger              TCATCAAGTCAAGCAAACCGTCTTGTATGGTACAGAAGTCTCTCTGTTTT
Consensus
```

```
Transcriptome NGS   --------------------------------------------------   8450
Viral RNA NGS       GGTGTCACACTCAATTTATTTGTTATCAGGCAACCAAACAAATCGTGCCT
Sanger              GGTGTCACACTCAATTTATTTGTTATCAGGCAACCAAACAAATCGTGCCT
Consensus

Transcriptome NGS   --------------------------------------------------   8500
Viral RNA NGS       TTTCAGTTCCAGATCTTTGTATAACTGGCCCAATTCTAGAGCTAGTTTCT
Sanger              TTTCAGTTCCAGATCTTTGTATAACTGGCCCAATTCTAGAGCCAGTTTCT
Consensus

Transcriptome NGS   --------------------------------------------------   8550
Viral RNA NGS       CAATCTTATCAAAGTCAACGTTGTAATTGTCACGATAGTAATTTTTGGCA
Sanger              CAATCTTATCAAAGTCAACGTTGTAATTGTCACGATAGTAATTTTTGGCA
Consensus

Transcriptome NGS   --------------------------------------------------   8600
Viral RNA NGS       TGTTTATCAACTTCTTGCATGATGGCTTGCATTTTGTAGACCAAGCCATG
Sanger              TGTTTATCAACTTCTTGCATGATGGTTTGCATTTTGTAGACCAAGCCATG
Consensus

Transcriptome NGS   --------------------------------------------------   8650
Viral RNA NGS       TATTTGAGAGAATATGTGCACTGCATGCTTGTGTCCATAATATTCAAAAC
Sanger              TATTTGAGAGAATATGTGCACTGCATGCTTGTTTCCATAATATTCAAAAC
Consensus

Transcriptome NGS   --------------------------------------------------   8700
Viral RNA NGS       TTTTCATTAAATCATTCACGGTTAATTCCAGTGTTTTTATTTCCCTAAGC
Sanger              TTTTCATTAAATCATTCGCGGTTAATTCCAGTGTTTTTATTTCCCTAAGC
Consensus
                                                         RdRp <-┐
Transcriptome NGS   --------------------------------------------------   8750
Viral RNA NGS       GTTTCAATGTTCTTATCAACAAAATTTGATAACACATTATGATTCATTTT
Sanger              GTTTCAATGTTCTTATCAACAAAATTTGATAACACATTATGATTCATTTT
Consensus

Transcriptome NGS   ----------------------------------------------      8800
Viral RNA NGS       GGTTCAAGTATTTGGTTAAGGTTATATTTTGTTGCGTGATTGC---
Sanger              GGTTCAAGTATTTGGTTAAGGTTATATTTTGTTGCGTGATTGCTCT
Consensus
```

S4 Fig. Comparison of the sequences of the Groundnut chlorotic fan-spot virus (GCFSV) L RNA as determined by next-generation sequencing (NGS) and Sanger sequencing methods. The nucleotide sequences from the transcriptome and viral RNA sequenced by RNA-seq are aligned with those sequenced by the Sanger method for comparison. The underlines indicate the nucleotides that are lost in the obtained sequences. The bold characters represent the nucleotides that are diverse among the three sequences.
