# Supplementary material for: Characterization of the genome of a phylogenetically distinct tospovirus and its interactions with the local lesion-induced host Chenopodium quinoa by whole-transcriptome analyses
Source: PLoS One. 2017 Aug 3;12(8):e0182425. doi: 10.1371/journal.pone.0182425 (PMC5542687; doi:10.1371/journal.pone.0182425)
Supplement: S1 Table — (PDF) [file pone.0182425.s005.pdf]

S1 Table. Primers used for the genome sequencing of Groundnut chlorotic fan-spot virus.

Primer name	Direction[a]	Sequence (5′→3′)	Annealing temp. (°C)
L RNA			
gL3637	F	CCTTTAACAGT(A/T/G)GAAACAT	52
gL4510c	R	CAT(A/T/G)GC(A/G)CAAGA(A/G)TG(A/G)TA(A/G)ACAGA	52
gL2740	F	ATGGG(A/G/T)AT(A/T/G/C)TTTGATTTCATG(A/G)TATGC	52
gL3920c	R	TCATGCTCAT(C/G)AG(A/G)TAAAT(T/C)TCTCT	52
GC-L385	F	GAGTGTGACACCAAAACAGAGAGAC	58
GC-L727c	R	CAGAATGCAAAGGACCACATAGATAGG	58
GC-L1991c	R	TGCTTATGTCCTGCTCTCCATCAC	58
GC-L2825c	R	GACGTTTGAGTAATCAGCTAATGG	58
GC-L2932c	R	AAGGCTCATTCCCATCATCTTATTC	58
GC-L4420	F	CTGGTATCCAGTAAGCATGAACTGG	58
GC-L6314	F	CTGAGCACAGTGTATAGCAGAGAAGC	58
GC-L7632	F	CACCAAGACCAACTGGTAGAAGCAT	60
GC-L7710	F	GCATCCTAAGATTGGTGAAAATGGCTG	60
GC-L8014c	R	TTCAGGGTCTTCTTCTATCCCAGC	58
M RNA			
GC-M66	F	GGTAAATATGGACATGATCAGCAGGATAGG	55
GC-M351c	R	GACTTCCAGCCTCTCTAGTATGCTCAAATCTG	55
GC-M506c	R	GCCACTGGAACAATCCAGATTATAACTC	55
GC-M1233	F	GGGAGAAATCCCCAAATTGGGACCTAATG	55
GC-M1548c	R	CGACAATGACAGTGATGTTGACGATTCTG	55
GC-M2375	F	GAGGATGACCAAACGTCATGTCTGCACT	55
GC-M2612c	R	GTTAAGGAGGTCCATGTTTCATCAACAGC	55
GC-M3054	F	GCTGGACACATCTAACTTGTCTAGTGAAGC	55
GC-M3780	F	GGACAATCATGAGACAGCAGAGACAA	55
GC-M3798c	R	GTCTCTGCTGTCTCATGATTGTCCA	55
GC-M4153	F	GATTGAACCTTAGAGTCAAGCTGACC	55
GC-M4357c	R	GGCTACTTGAAGTAGGTAGTAAATACTTCG	55
GC-M4367	F	CCACATTCATGACAGGGACCACTGGAATTGT	55
M3′UTR(19)	R	AGAGCAATCRGTGCAACAA	55
S RNA			
PNSs68Nhe	F	GGCTAGCATGTCTACCGAGTCGTCC	50
PNSs1483cXho	R	GCTCGAGATTGAAGATAAAAACATTAT	50
PN1945K	F	AAGGTACCAAACAGAAAAGCTTTAACAATCAA	58
PN2754cS	R	GGGCATGCATGTCTAAAACCAAAGTCAAGAAT	58

[a] “F” and “R” represent the primers were designed in forward and reverse directions, respectively.
