# Supplementary material for: Characterization of the genome of a phylogenetically distinct tospovirus and its interactions with the local lesion-induced host Chenopodium quinoa by whole-transcriptome analyses
Source: PLoS One. 2017 Aug 3;12(8):e0182425. doi: 10.1371/journal.pone.0182425 (PMC5542687; doi:10.1371/journal.pone.0182425)
Supplement: S2 Table — (PDF) [file pone.0182425.s006.pdf]

**S2 Table.** The accession numbers of the L and M RNA sequences of tospoviruses used for the analyses in this study.

| Species | Abbreviation | L RNA | M RNA |
|---|---|---|---|
| Bean necrosis mosaic virus | BeNMV | JF417980 | JN587269 |
| Calla lily chlorotic spot virus | CCSV | FJ822962 | FJ822961 |
| Capsicum chlorosis virus | CaCV | NC_008302 | NC_008303 |
| Chrysanthemum stem necrosis virus | CSNV | KF493773 | KF493772 |
| *Groundnut bud necrosis virus* | GBNV | AF025538 | NC_003620 |
| Groundnut chlorotic fan-spot virus | GCFSV | KP146140 | KP146141 |
| *Groundnut ringspot virus* | GRSV | KT972590 | NSm: AF513220<br>GP: AY574055 |
| Hippeastrum chlorotic ringspot virus | HCRV | HG763861 | JX833565 |
| *Impatiens necrotic spot virus* | INSV | NC_003625 | NC_003616 |
| *Iris yellow spot virus* | IYSV | FJ623474 | AF214014 |
| Melon severe mosaic virus | MeSMV | NC_033834 | NC_033833 |
| Melon yellow spot virus | MYSV | NC_008306 | NC_008307 |
| Pepper chlorotic spot virus | PCSV | NC_033774 | NC_033773 |
| *Polygonum ringspot virus* | PolRSV | KJ541746 | KJ541745 |
| Soybean vein necrosis-associated virus | SVNaV | HQ728385 | HQ728386 |
| *Tomato chlorotic spot virus* | TCSV | HQ700667 | NSm: AF213674<br>GP: AY574054 |
| Tomato necrotic ringspot virus | TNRV | - | FJ947152 |
| Tomato necrotic spot associated virus | TNSaV | KT984754 | KT984753 |
| *Tomato spotted wilt virus* | TSWV | AB190813 | AF208497 |
| Tomato yellow ring virus | TYRV | JN560178 | JN560177 |
| Tomato zonate spot virus | TZSV | EF552435 | EF552434 |
| *Watermelon bud necrosiClustalW_s virus* | WBNV | GU735408 | GU584185 |
| *Watermelon silver mottle virus* | WSMoV | NC_003832 | NC_003841 |
| *Zucchini lethal chlorosis virus* | ZLCV | NC_031762 | NSm: AF213676<br>GP: AB274027 |

* Italic typing represents official species and standard typing represents tentative species.

** "-" represents no genomic sequences are available in GenBank.
