# Supplementary material for: Characterization of the genome of a phylogenetically distinct tospovirus and its interactions with the local lesion-induced host Chenopodium quinoa by whole-transcriptome analyses
Source: PLoS One. 2017 Aug 3;12(8):e0182425. doi: 10.1371/journal.pone.0182425 (PMC5542687; doi:10.1371/journal.pone.0182425)
Supplement: S3 Table — (PDF) [file pone.0182425.s007.pdf]

S3 Table. The primers used for the quantitative analyses of replication of Groundnut chlorotic fan-spot virus (GCFSV) in *Chenopodium quinoa* leaves by real-time reverse transcription-polymerase chain reaction.

Primer name	Sequence (5'→3')	Target gene
GC-NSm656	ACAAAGCTCTCTGCCCTCAACTTAA	GCFSV *NSm*
GC-NSm855c	ACACTTCACAGCTTCGAGGAGAGCT	
GC-Gn2123	GCTCAATAGGCAGATCCAGAACAGT	GCFSV *Gn/Gc*
GC-Gn2324c	GAACAGCTGTCATGGGACTGTGAGT	
GC-NSs735	CAAGAGGACCCAAGAAAAGGCTCAT	GCFSV *NSs*
GC-NSs938c	CTGGGATGGCAATAAGATGCTCTCT	
GC-N2295	GAGGTGATTCAAGTTGCCTCCAGTC	GCFSV *N*
GC-N2496c	ATTCACTTGGAGCAGGATGGACTCT	
GC-L7061	GCCACTTTTTACCCTCACTAGTCC	GCFSV *RdRp*
GC-L7353c	GTCTCCTCACAGAGAAGATGTCGT	
nad5-s	GATGCTTCTTGGGGCTTCTTGTT	*Cqnad5*
nad5-as	CTCCAGTCACCAACATTGGCATAA	
Cq-GAPDH-458f	GTTGTACCACCAACTGTCTTGCTC	*CqGAPDH*
Cq-GAPDH-602r	CCACCTCTCCAATCCTTAGCTGAT	
