# Supplementary material for: Characterization of the genome of a phylogenetically distinct tospovirus and its interactions with the local lesion-induced host Chenopodium quinoa by whole-transcriptome analyses
Source: PLoS One. 2017 Aug 3;12(8):e0182425. doi: 10.1371/journal.pone.0182425 (PMC5542687; doi:10.1371/journal.pone.0182425)
Supplement: S4 Table — (PDF) [file pone.0182425.s008.pdf]

**S4 Table.** The primers used for the relative quantitative assays of hypersensitive response-related gene expression of *Chenopodium quinoa* leaves infected with Groundnut chlorotic fan-spot virus in real-time reverse transcription-polymerase chain reaction.

| Primer name | Sequence (5′→3′) | Target gene | Primer name | Sequence (5′→3′) | Target gene |
|---|---|---|---|---|---|
| CqBG1.2-76f | GTATGTTATGGGGGCAATGGAG | *CqBG1* | CqRTE1.2-20f | CTGGAATGGGTTTAGAAGCAGG | *CqRTE1* |
| CqBG1.2-271r | CTGATTGATCCGACCCTAGAGACT | | CqRTE1.2-158r | CATGAGACAACTGGAAGTGGAGTC | |
| CqCHIB-802f | GGTGGGATCGAATGTGGTAAAG | *CqCHIB* | CqSNAP33.1-797f | GCAATCTTCTAGGGGAGCTGAAG | *CqSNAP33* |
| CqCHIB-927r | GGGTTGCTGTGAGTAGCAGTCTAA | | CqSNAP33.1-932r | CCACGTTGTGTAGCACCTCTAACT | |
| CqEBF2.1-1158f | GGGTAAGATGTGTCCAAGCCTAAG | *CqEBF2* | CqSOBIR1-75f | GATATACCTCGACCCATCCGACTA | *CqSOBIR1* |
| CqEBF2.1-1327r | GGAGTCTAGCAACAGCAAGAACAG | | CqSOBIR1-196r | GCTCACAAGAAACAGCAGGAGAG | |
| CqERF1.1-575f | ACTTCCCCCTAAGAGTAAACTCGG | *CqERF1* | CqSYR1.1-282f | AGTAAGGTTGGAAGCGTTGGAC | *CqSYR1* |
| CqERF1.1-749r | GCTTCCACTCCTTTTCCCACTT | | CqSYR1.1-469r | CAACAGTCTCCCTATATTCCCCAC | |
| CqMKK1-664f | CAGGCTAACACTAGAATCGGGAC | *CqMKK1* | CqWRKY42-207f | TGCTACTGCTACTGCTACTCCTGC | *CqWRKY42* |
| CqMKK1-816r | ATCTTCATCTGGTAGTGGGTAGGG | | CqWRKY42-309r | GCTTCCCGTATTAGTGGTGAGAAG | |
| CqPLA2A.1-887f | CTAGTGGTGACATGGTGGATCTTC | *CqPLA2A* | CqWRKY53-625f | CCTACACCCAAATCCGAACCTATTG | *CqWRKY53* |
| CqPLA2A.1-1051r | ACTCTTCCCCAGCTTTCACTAGGT | | CqWRKY53-839r | ATGGATTAGAGGATGAAGGGAAGGA | |
| CqPRB1-128f | CGTGGGTGTTGGTAGCATACAATG | *CqPRB1* | CqWRKY75.1-207f | GTCGGAGAAGAAGGCAAAGAAG | *CqWRKY75* |
| CqPRB1-410r | ACCCTAGCACATCCAAGACGAATC | | CqWRKY75.1-356r | TGATACGTGCACCTGTAGTAGCTC | |
