# Supplementary material for: Characterization of the genome of a phylogenetically distinct tospovirus and its interactions with the local lesion-induced host Chenopodium quinoa by whole-transcriptome analyses
Source: PLoS One. 2017 Aug 3;12(8):e0182425. doi: 10.1371/journal.pone.0182425 (PMC5542687; doi:10.1371/journal.pone.0182425)
Supplement: S5 Table — (PDF) [file pone.0182425.s009.pdf]

S5 Table. Single nucleotide polymorphisms (SNPs) of genomic sequences of Groundnut chlorotic fan-spot virus (GCFSV), as determined by next-generation sequencing (NGS).

RNA region	Nucleotide position	Sequence obtained from	
		Poly(A)-containing RNA	Viral RNA
S RNA			
5'-UTR	19	A(469)/T(69)/C(12)/G(8)	-
NSs	307	T(2959)/C(39)/G(8)/A(4)	T(176879)/C(139)/G(48)/A(17)
	731	C(3186)/T(1422)/A(34)/G(11)	C(155732)/T(1422)/A(34)/G(11)
	892	A(3822)/C(3)/T(1)	A(153670)/C(14)/G(130)
	921	A(3096)/G(14)/T(8)	A(75512)/G(281)/T(12)/C(14)
	922	G(3108)/A(7)/C(1)/T(1)	G(75527)/T(24)/A(4)/C(1)
	940	C(3037)/T(18)/A(16)/G(8)	C(112135)/T(87)/A(15)/G(3)
	1027	C(3176)/G(22/)T(8)/A(3)	C(80869)/T(41)/A(21)/G(10)
	1143	C(3660)/T(60)/G(24)/A(10)	C(112781)/T(53)/A(25)/G(5)
IGR	1892	G(2757)/A(129)/T(26)/C(15)	G (118749)/A(203)/T(49)/C(15)
N	2035	C(3618)/G(3)/A(1)	C(131292)/T(105)/A(35)
M RNA			
5'-UTR	8	-	T(4775)/G(1)
NSm	879	A(46)	A(28794)/G(121)/C(28)/T(10)
IGR	1164	-	C(34581)/A(392)/T(133/)G(3)
	1166	-	A(35633)/C(169)/T(23)/G(5)
	1449	G(49)	G(34904)/A(29)/T(10)/C(4)
Gn/Gc	1476	A(50)	A(36047)/T(10)/G(8)/C(4)
	2189	T(41)/C(1)	T(42303)/C(12)/G(5)/A(1)
	2234	A(28)/G(6)	G(42966)/A(380)/T(19)/C(6)
	2364	C(61)	C(49929)/T(19)/G(5)/A(3)
	2578	G(29)/A(13)/T(1)	A(23460)/G(96)/T(15)/C(7)
	2699	G(41)/A(17)	A(50753)/C(15)/G(10)/T(6)
	2831	T(46)	T(40308)/C(92)/A(13)/G(5)
	3289	C(31)T(2)A(1)	T(48446)/C(255)/G(21)/A(7)
	3873	A(46)G(1)	A(32592)/G(211)/C(13)/T(2)
	4653	C(37)T(1)	T(49183)/C(33)/G(15)/A(4)
	4657	G(34)T(1)	G(47721)/A(32)/C(6)/T(6)
3'-UTR	4824	A(36)G(1)	T(30272)/A(1845)/G(11)/C(8)
L RNA			
RdRp	690	-	A(9401)/T(4)/C(4)/G(3)

1460	-	C(9046)/T(1576)/G(1)
4417	A(8)	A(3482)/G(3)/T(1)
4758	T(7)	T(4270)/C(2)
5051	C(1)	C(2564)/T(574)
5390	G(11)	G(16776)/A(11)/T(9)
5802	A(5)	G(6574)/A(4949)/C(4)/T(4)
5847	A(9)	A(13734)/C(5)/G(3)
5976	A(4)	A(12313)/G(6)/T(1)
5985	G(3)/A(1)	A(12168)/C(5)/G(4)
6179	A(8)	A(4834)/G(9)/T(1)/C(1)
6182	T(8)	T(4839)/C(4)/A(1)/G(1)
6581	T(25)	T(5058)/C(4)/A(2)/G(2)
8493	T(15)	T(4023)/C(935)/A(2)
8576	C(5)	C(6522)/A(3)/T(2)/G(1)
8633	G(8)	G(4116)/T(1297)/C(3)/A(1)
8668	A(10)/C(1)	A(5904)/G(14)/C(4)/T(2)

* The reads frequencies of individual nucleotides are represented in parentheses. No nucleotides obtained in NGS are indicated as "-".
