# Supplementary material for: Characterization of the genome of a phylogenetically distinct tospovirus and its interactions with the local lesion-induced host Chenopodium quinoa by whole-transcriptome analyses
Source: PLoS One. 2017 Aug 3;12(8):e0182425. doi: 10.1371/journal.pone.0182425 (PMC5542687; doi:10.1371/journal.pone.0182425)
Supplement: S6 Table — (PDF) [file pone.0182425.s010.pdf]

**S6 Table.** Significant Gene Ontology (GO) terms from the gene set enrichment analysis for the 4,091 Groundnut chlorotic fan-spot virus-responsive genes (false discovery rate, FDR < $10^{-25}$).

| GO term | Ontology | Description | FDR |
|---|---|---|---|
| GO:0044281 | P | Small molecule metabolic process | 5e-49 |
| GO:0042180 | P | Cellular ketone metabolic process | 2.7e-37 |
| GO:0005975 | P | Carbohydrate metabolic process | 4.2e-37 |
| GO:0043436 | P | Oxoacid metabolic process | 4.2e-37 |
| GO:0019752 | P | Carboxylic acid metabolic process | 4.2e-37 |
| GO:0006082 | P | Organic acid metabolic process | 4.3e-37 |
| GO:0050896 | P | Response to stimulus | 1e-35 |
| GO:0008152 | P | Metabolic process | 8.5e-35 |
| GO:0034641 | P | Cellular nitrogen compound metabolic process | 1.4e-33 |
| GO:0044262 | P | Cellular carbohydrate metabolic process | 4.3e-33 |
| GO:0006066 | P | Alcohol metabolic process | 6e-33 |
| GO:0044283 | P | Small molecule biosynthetic process | 9.2e-31 |
| GO:0032787 | P | Monocarboxylic acid metabolic process | 1.4e-29 |
| GO:0019748 | P | Secondary metabolic process | 4.8e-29 |
| GO:0005996 | P | Monosaccharide metabolic process | 7.8e-29 |
| GO:0042221 | P | Response to chemical stimulus | 8.8e-29 |
| GO:0044237 | P | Cellular metabolic process | 3.9e-28 |
| GO:0051186 | P | Cofactor metabolic process | 1.1e-26 |
| GO:0006950 | P | Response to stress | 1.3e-26 |
